# Design, synthesis, and biological evaluation of novel triazoloquinazolinone derivatives as SHP2 protein inhibitors

**DOI:** 10.1080/14756366.2021.1986491

**Published:** 2021-11-08

**Authors:** Rongshuang Luo, Zhongyuan Wang, Dali Luo, Yumei Qin, Chunshen Zhao, Di Yang, Tian Lu, Zhixu Zhou, Zhuyan Huang

**Affiliations:** a School of Pharmaceutical Sciences, Guizhou University, Guiyang, China; b Guizhou Engineering Laboratory for Synthetic Drugs, Guiyang, China; c Department of Pharmacy, Guizhou Provincial People’s Hospital, Guiyang, China; d School of Chinese Materia Medica, Nanjing University of Chinese Medicine, Nanjing, China; e Department of Dermatology, Affiliated Hospital of Guizhou Medical University, Guiyang, China

**Keywords:** Triazoloquinazolinone derivatives, SHP2 inhibitors, A375 cell line, antitumor activity

## Abstract

A novel series of triazoloquinazolinone derivatives were designed, synthesised, and evaluated for their *in vitro* biological activities against the SHP2 protein. Moreover, some compounds were evaluated against A375 cells. The results revealed that target compounds possessed moderate to excellent inhibitory activity against SHP2 protein, whereas compounds **12f**, **12l**, **12j**, **17e**, and **17f** have strong antiproliferative activity on A375 cells. The compound **12l** showed remarkable cytotoxicity against A375 cells and a strong inhibitory effect against SHP2 protein when compared with **SHP244**. The structure-activity relationships (SARs) indicated that electron-donating groups (EDGs) on phenyl rings are beneficial for improving the antitumor activity; compounds with a hydroxyl substituent at the 2-position of phenyl ring exhibited superior activities than compounds with a substituent at the 4-position. In addition, compound **12l** displayed improved physicochemical properties as well as metabolic stability compared to **SHP244**. Our efforts identified **12l** as a promising SHP2 protein inhibitor, warranting its further investigation.

**ARTICLE HISTORY**
Received 20 April 2021
Revised 30 August 2021
Accepted 22 September 2021

## 1. Introduction

Malignant tumours pose a serious threat to human health. However, traditional chemotherapeutic agents are extremely limited in clinical application, given their poor selectivity and immense potential for side effects. Therefore, it is critical to identify and develop novel targeted anti-tumour drugs based on unique mechanisms of action, new targets, higher selectivity, and fewer toxic side effects[1–7].

The regulation of protein reversible phosphorylation is the most extensive and universal regulatory process during cell signal transduction, modulated by protein kinases and phosphatases. Moreover, mechanisms underlying reversible phosphorylation deregulation can result in several diseases, including inflammation and tumors[8–10]. Among them, protein tyrosine kinases (PTKs) and protein tyrosine phosphatases (PTPs) are involved in the regulation of tyrosine phosphorylation and dephosphorylation, it was once considered to play a positive and negative regulation of tumour cell proliferation[11–14]. SHP2 is an important member of the PTPs family and is encoded by the protein tyrosine phosphatase non-receptor type 11 (PTPN11) gene. X-ray crystallographic studies have revealed that SHP2 is self-inhibited in an inactive state. However, when it was stimulated by growth factors, cytokines, or inflammatory factors, it will be activated and participates in diverse signal transduction cascades, leading to the occurrence of a variety of diseases including tumors[15–17]. Reportedly, activation of SHP2 mutations can be closely associated with the onset of Noonan syndrome, leopard spot syndrome, immature myelomonocytic leukaemia, melanoma, and solid cancer, et al[18–20]. Accordingly, SHP2 could be used as an important target for antitumour drug therapy, and it is significant to control the activity of SHP2 for tumour treatment.

Based on distinct sites of action, SHP2 small molecule inhibitors can be divided into two categories: catalytic site inhibitors and allosteric site inhibitors[21]. Allosteric site inhibitors have attracted widespread attention owing to their superior selectivity and bioavailability. Three potential small molecule binding sites have been identified in the coordination pocket of SHP2 allosteric inhibitors, including the reported "tunnel" allosteric site A, the predicted "latch-up" allosteric site B at the PTP domain interface on both sides of *N*-SH2, and the "groove" allosteric site C on the other side of the protein, respectively. Allosteric site B is the latest allosteric site of SHP2, and few studies on its inhibitors at home and abroad. Hence, it is crucial to develop the targeted inhibitors of allosteric site B. Recently, the development of allosteric site inhibitors, including SHP836(**1**), SHP099(**2**), SHP389(**3**), SHP244(**4**), SHP844(**5**), and SHP504(**6**), has gained considerable momentum (Figure 1). Compounds **4**, **5,** and **6** were geared towards targeted inhibitors of allosteric site B[11,12,22–25], and compound **4** was developed by Novartis Pharmaceuticals in 2018. Given that the structure could be modified with a large space and few reports available on compound **4**, it affords great potential for in-depth research. As an extension of our previous research to develop novel potent SHP2 inhibitors, we aimed to establish a novel

**CONTACT** Zhixu Zhou zhixuzhou@126.com; Zhuyan Huang 785596957@qq.com Guizhou Engineering Laboratory for Synthetic Drugs, School of Pharmaceutical Sciences, Guizhou University, Guiyang, 550025, China

Supplemental data for this article can be accessed here.

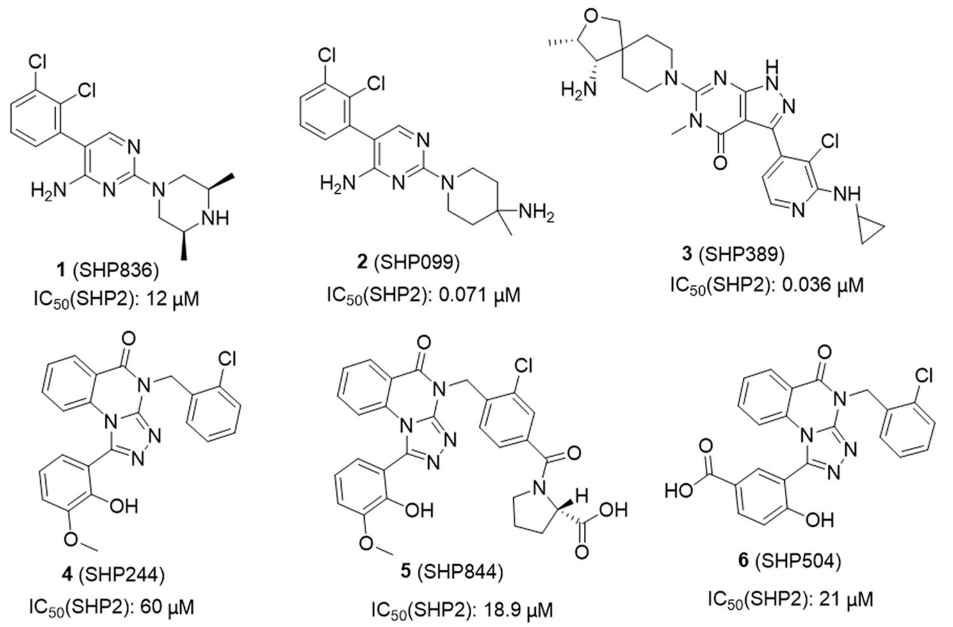

**Figure 1.** Structures of some targeted SHP2 inhibitors.

scaffold for further exploration. X-ray crystal structure of compound **4** in complexes with SHP2 demonstrated that the interaction between compound **4** and SHP2 protein occurred through hydrogen bonding (N…H, O…H), $\pi$-stacking, and water-mediated, etc[12,26]. These results confirmed its anti-tumour activity. However, the highly conjugated tricyclic structure of compound **4** resulted in poor water solubility.

Based on the aforementioned facts, a novel series of SHP2 inhibitors were designed and synthesised. Polar functional groups containing heteroatoms were introduced into the phenyl ring to enhance hydrogen bonding between target compounds and the amino acid residue LYS266 as well as to improve the water solubility. In addition, we adjusted the positions of phenolic hydroxyl groups and explored their effects on the inhibitory activity of SHP2 protein. Finally, various substituents were introduced to the terminal phenyl ring to determine the influence of electron density on the anti-cancer activity (Figure 2). In the present study, we synthesised these derivatives and evaluated their inhibitory activity against SHP2 protein kinase. Furthermore, some compounds were evaluated antiproliferative activity *in vitro* against A375 cells. Compound **12l** was identified as a potent SHP2 inhibitor with excellent *in vitro* efficacy, thus presenting a promising drug candidate for further evaluation.

## 2. Docking studies

Detailed docking analysis was performed to further elucidate the binding mode of compounds. The three-dimensional protein crystal structure of the SHP2 target protein (PDB ID: 6BMR) was downloaded from the RCSB PDB protein database (https://www.rcsb.org/), and the protein was prepared as a receptor model using SYBYL-X 2.0. In addition, the target compound was directly mapped to SYBYL-X 2.0 as the corresponding ligand. The docking simulation was performed using Surflex-Dock (SFXC). The binding models were exemplified by the interaction of compounds **4** and **12d** with the SHP2 protein. As shown in Figure 1, the nitrogen atoms of triazole at **4** and **12d** formed hydrogen-bonding interactions with ARG265, GLN269, and the NH of the main chain. Moreover, the models further suggested that the phenolic hydroxyl groups at **4** and **12d** formed H-bonds with GLN79, LEU262, and ARG265, and the methoxy group was combined with GLN79 through H-bonds mediated by a water molecule (H2O899). Besides, the $\pi$-stacking interactions were formed between chlorophenyl and GLN269, quinazolinone benzene ring, and GLN79. In the meantime, it is worth noting that the distance between the para-position of the phenolic hydroxyl group of compound **4** (the 5-position of phenyl ring) and LYS266 was 3.333 Å, which is not conducive for intermolecular hydrogen bond formation. However, oxygen and nitrogen atoms of the morpholine ring in compound **12d** formed three H-bond interactions with LYS266, indicating that the distance between the target compounds and LYS266 was shortened, thus promoting the formation of intermolecular hydrogen bonds Figure 3.

## 3. Experimental

### 3.1. Chemistry

The melting point of target compounds was determined using a BUCHI Melting Point B-545 apparatus and was uncorrected. Mass spectra were recorded on a Varian QFT-ESI and Bruker micro-TOFQ-Q mass spectrometer (for HR-ESIMS). Using TMS as the internal standard, $^{1}$H NMR and $^{13}$C NMR were collected on Bruker Avance 400 spectrometer (400 and 100 MHz, respectively) in $CDCl_3$ and DMSO-$d_6$. The reaction time and purity of products were monitored by TLC on FLUKA silica gel aluminium cards (0.2 mm thickness) with an Ultraviolet indicator of 254 nm. Unless otherwise indicated, all materials were obtained from commercially available sources and used without further purification.

### 3.2. General procedures

#### 3.2.1. 2-Amino-N-(2-chlorobenzyl)benzamide (8)

A mixture of isatinic anhydride (20.00 g, 122.60 mmol), *o*-chlorobenzylamine (17.36 g, 122.60 mmol), and ethyl acetate (200 ml) was heated to 40 °C for 2 h. After the completion of the reaction detected by TLC, the reaction solution was obtained, dried, and evaporated to afford compound **8** as a brown solid. The solid was

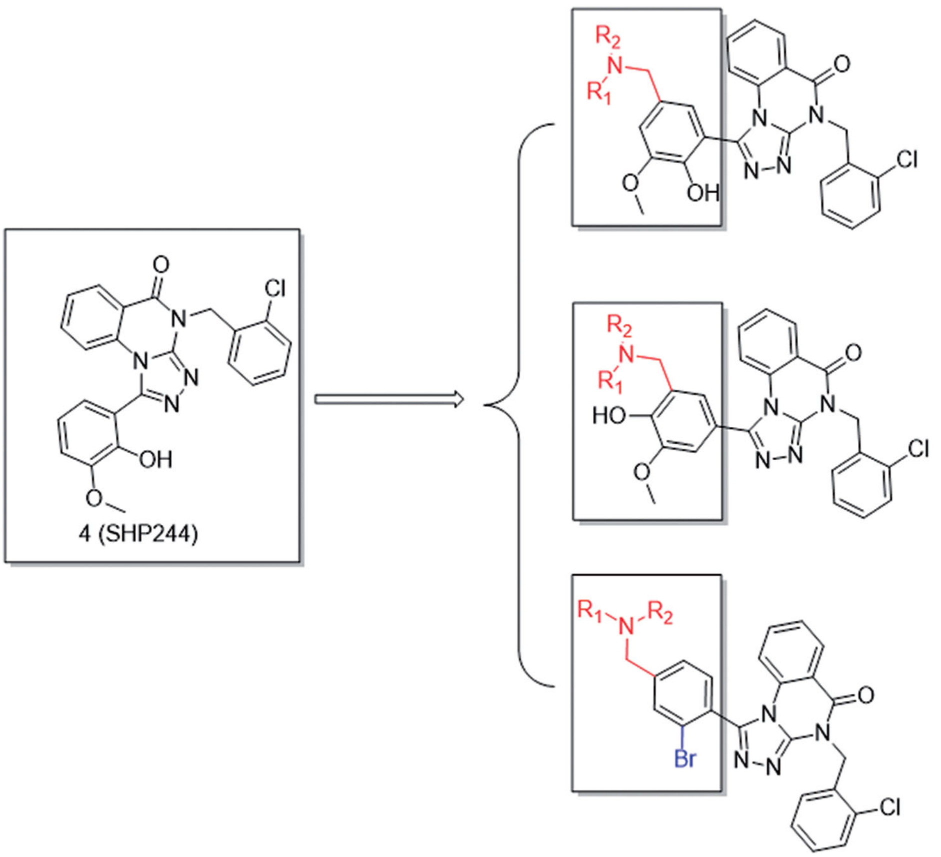

Figure 2. Structure of target compounds.

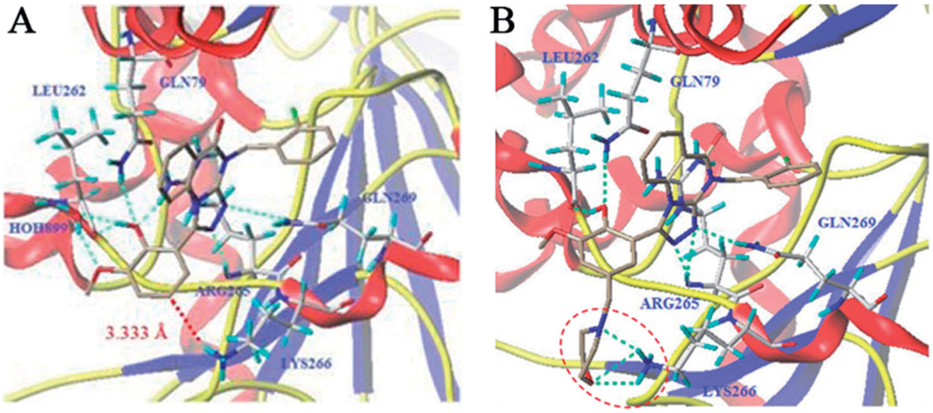

Figure 3. The docking results of compounds **4** (**A**) and **12d** (**B**) with SHP2 protein. Compound 4 was shown in coloured sticks (gray: carbon atom, blue: nitrogen atom, red: oxygen atom, green: Chlorine atom, light blue: hydrogen atom). Compound 12d was shown in coloured sticks (gray: carbon atom, blue: nitrogen atom, red: oxygen atom, light blue: a hydrogen atom, green: Chlorine atom). The H-bond interaction was shown in a light blue dotted line.

washed with water, methanol, and *n*-hexane twice in sequence. 98.2% yield; $^1$H NMR (400 MHz, $CDCl_3$) $\delta$ (ppm): 7.47–7.44 (m, 1H), 7.41–7.37 (m, 1H), 7.34 (d, $J=7.9$ Hz, 1H), 7.26–7.18 (m, 3H), 6.69–6.63 (m, 2H), 6.50 (s, 1H), 5.53 (s, 2H), 4.69 (d, $J=6.0$ Hz, 2H); HRMS (ESI-MS) *m/z*: 261.0786 $[M+H]^+$.

### *3.2.2. 3–(2-Chlorobenzyl)-2-thioxo-2,3-dihydroquinazolin-4(1H)-one (9)*

To a solution of compound **8** (30.00 g, 115.07 mmol) in ethanol (150 ml) was added potassium hydroxide (14.20 g, 253.15 mmol) aqueous solution and carbon disulphide (87.61 g, 1150.70 mmol), The reaction mixture was heated with stirring for 6 h at 55 °C. the reaction solution was cooled to room temperature when the reaction was completed, whereby a white precipitate was formed. The precipitant was filtered, washed with water and acetone, dried to afford the compound **9** as a white solid, 90.7% yield; $^1$H NMR (400 MHz, DMSO-$d_6$) $\delta$ (ppm): 13.16 (s, 1H), 7.97 (d, $J=7.5$ Hz, 1H), 7.80 (t, $J=7.6$ Hz, 1H), 7.48 (dd, $J=10.8$, 8.5 Hz, 2H), 7.38 (t, $J=7.6$ Hz, 1H), 7.28 (t, $J=7.1$ Hz, 1H), 7.22 (t, $J=7.4$ Hz, 1H), 6.97 (d, $J=7.4$ Hz, 1H), 5.67 (s, 2H); HRMS (ESI-MS) *m/z*: 303.0349 $[M+H]^+$.

### *3.2.3. (E)-3–(2-chlorobenzyl)-2-hydrazono-2,3-dihydroquinazolin-4(1H)-one (10)*

To a solution of an intermediate **9** (30.00 g, 99.08 mmol) in isopropanol (150 ml) was added 80% hydrazine hydrate (93.00 g, 1486.23 mmol) and heated at reflux for 16 h. After cooling, a large amount of white solid was precipitated. The solid was filtered, washed with water and methyl tert-butyl ether three times. then dried to afford the corresponding compound **10** for 25.62 g, 86.0% yield; $^{1}$H NMR (400 MHz, DMSO-$d_6$) $\delta$ (ppm): 7.89 (d, $J = 7.2$ Hz, 1H), 7.62 (s, 1H), 7.49 (d, $J = 7.7$ Hz, 1H), 7.38 (d, $J = 8.2$ Hz, 1H), 7.28 (t, $J = 7.2$ Hz, 1H), 7.22 (t, $J = 7.4$ Hz, 1H), 7.10 (s, 1H), 6.79 (s, 1H), 5.22 (s, 2H), 4.43 (s, 1H); HRMS (ESI-MS) *m/z*: 301.0847 $[M+H]^+$.

### 3.2.4. General procedure for preparation of triazoloquinazolinones (11, 13)

Compound **10** (10.00 g, 33.25 mmol), corresponding aryl formaldehyde (5.06 g, 33.25 mmol) and glacial acetic acid (0.50 ml) were successively added to a solution of isopropanol (200 ml). After stirring at 83 °C for 0.5 h. After cooling, the ferric chloride hexahydrate (44.94 g, 166.25 mmol) was added under stirring and the reaction mixture was heated for 1 h at 83 °C. Then the reaction mixture was poured into water (800 ml) when it cool to room temperature. After stirring for 0.5 h, a large amount of brown-gray solid was precipitated. The precipitant was filtered, washed with water, acetone and methyl tert-butyl ether twice and dried to afford the corresponding compound **11**, **13**.

### 3.2.5. 4–(2-Chlorobenzyl)-1–(2-hydroxy-3-methoxyphenyl)-[1,2,4]triazolo[4,3-a] quinazolin-5(4H)-one (11)

Off-white solid; yield: 86.2%; $^{1}$H NMR (400 MHz, DMSO-$d_6$) $\delta$ (ppm): 9.52 (s, 1H), 8.27 (d, $J = 7.7$ Hz, 1H), 7.74 (t, $J = 7.7$ Hz, 1H), 7.56 (t, $J = 7.9$ Hz, 2H), 7.34 (t, $J = 7.5$ Hz, 1H), 7.30–7.24 (m, 2H), 7.20 (d, $J = 8.4$ Hz, 2H), 7.08–7.00 (m, 2H), 5.49 (s, 2H), 3.91 (s, 3H); HRMS (ESI-MS) *m/z*: 433.1058 $[M+H]^+$.

### 3.2.6. 4–(2-Chlorobenzyl)-1–(4-hydroxy-3-methoxyphenyl)-[1,2,4]triazolo[4,3-a] quinazolin-5(4H)- one (13)

White solid; yield: 98.7%; $^{1}$H NMR (400 MHz, DMSO-$d_6$) $\delta$ (ppm): 9.76 (s, 1H), 8.26 (d, $J = 7.1$ Hz, 1H), 7.74 (t, $J = 7.9$ Hz, 1H), 7.58–7.53 (m, 2H), 7.34 (t, $J = 6.0$ Hz, 1H), 7.28–7.21 (m, 4H), 7.11 (d, $J = 8.0$ Hz, 1H), 7.02 (d, $J = 8.1$ Hz, 1H), 5.47 (s, 2H), 3.77 (s, 3H); HRMS (ESI-MS) *m/z*: 433.1056 $[M+H]^+$.

### 3.2.7. General procedure for the preparation of triazoloquinazolinone derivatives (12a–12m, 14a–14m)

A mixture of compounds **11**, **13** (1.00 g, 2.31 mmol), glacial acetic acid (20 ml) and the mixture of a freshly prepared 40% aqueous solution of dimethylamine (1.04 g, 9.24 mmol) and a 37% aqueous solution of formaldehyde (0.75 g, 9.24 mmol) was refluxed for 6 h. The solvent was concentrated under vacuum and the residue was poured into stirring ice-water (100 ml), basified with sodium hydroxide solution to pH 8–9, the precipitate was filtered off, and the filtrate was extracted with dichloromethane (90 ml). The organic phases were combined and were washed with water (30 ml), dried over anhydrous $Na_2SO_4$, concentrated under reduced pressure to afford brown solid, it was washed twice with methyl *tert*-butyl ether and dried to obtain corresponding triazoloquinazolinones **12a–12m** and **14a–14m**.

### 3.2.8. 4–(2-Chlorobenzyl)-1-{5-[(dimethylamino)methyl]-2-hydroxy-3-methoxyphenyl} -[1,2,4] triazolo[4,3-a]quinazolin-5(4H)-one (12a)

Light brown solid; yield: 75.2%; m.p.:140 – 143 °C; $^{1}$H NMR (400 MHz, DMSO-$d_6$) $\delta$ (ppm): 9.43 (s, 1H), 8.27 (d, $J = 7.6$ Hz, 1H), 7.76–7.71 (m, 1H), 7.56 (dd, $J = 7.4$, 4.4 Hz, 2H), 7.37–7.32 (m, 1H), 7.30–7.11 (m, 4H), 6.94 (s, 1H), 5.50 (d, $J = 16.3$ Hz, 2H), 3.90 (s, 3H), 3.38 (s, 2H), 2.17 (s, 6H); $^{13}$C NMR (100 MHz, DMSO-$d_6$) $\delta$ (ppm): 158.80, 155.29, 150.28, 148.69, 148.50, 148.31, 147.15, 144.79, 135.34, 134.56, 133.48, 132.21, 129.81, 129.49, 129.38, 127.84, 127.11, 124.25, 123.27, 117.27, 115.99, 63.40, 56.56, 45.32, 44.32; HRMS (ESI-MS) *m/z*: 490.1635 $[M+H]^+$.

### 3.2.9. 4–(2-Chlorobenzyl)-1-{5-[(diethylamino)methyl]-2-hydroxy-3-methoxyphenyl}-[1,2,4] triazolo[4,3-a]quinazolin-5(4H)-one (12 b)

Off-white solid; yield: 57.5%; m.p.:117–120 °C; $^{1}$H NMR (400 MHz, DMSO-$d_6$) $\delta$ (ppm): 9.37 (s, 1H), 8.27 (d, $J = 9.3$ Hz, 1H), 7.73 (t, $J = 7.9$ Hz, 1H), 7.59–7.54 (m, 2H), 7.37–7.32 (m, 1H), 7.26 (t, $J = 7.9$ Hz, 1H), 7.21 (t, $J = 9.1$ Hz, 3H), 6.99 (s, 1H), 5.49 (d, $J = 13.7$ Hz, 2H), 3.90 (s, 3H), 3.54 (s, 2H), 2.48 (d, $J = 7.5$ Hz, 4H), 0.98 (t, $J = 7.1$ Hz, 6H); HRMS (ESI-MS) *m/z*: 518.1951 $[M+H]^+$.

### 3.2.10. 4–(2-Chlorobenzyl)-1-[2-hydroxy-3-methoxy-5-(pyrrolidin-1-ylmethyl)phenyl]-[1,2,4] triazolo[4,3-a]quinazolin-5(4H)-one (12c)

White solid; yield: 64.7%; m.p.:192–194 °C; $^{1}$H NMR (400 MHz, DMSO-$d_6$) $\delta$ (ppm): 9.35 (s, 1H), 8.27 (d, $J = 8.1$ Hz, 1H), 7.73 (t, $J = 8.0$ Hz, 1H), 7.56 (t, $J = 7.7$ Hz, 2H), 7.34 (t, $J = 7.8$ Hz, 1H), 7.26 (t, $J = 7.0$ Hz, 1H), 7.23–7.17 (m, 3H), 6.98 (s, 1H), 5.50 (d, $J = 12.7$ Hz, 2H), 3.90 (s, 3H), 3.57 (s, 2H), 2.46 (s, 4H), 1.69 (s, 4H); HRMS (ESI-MS) *m/z*: 516.1792 $[M+H]^+$.

### 3.2.11. 4–(2-Chlorobenzyl)-1-[2-hydroxy-3-methoxy-5-(morpholinomethyl)phenyl]-[1,2,4]triazolo [4,3-a]quinazolin-5(4H)-one (12d)

White solid; yield: 74.8%; m.p.:190–193 °C; $^{1}$H NMR (400 MHz, DMSO-$d_6$) $\delta$ (ppm): 9.44 (s, 1H), 8.26 (d, $J = 9.3$ Hz, 1H), 7.73 (t, $J = 7.9$ Hz, 1H), 7.58–7.53 (m, 2H), 7.34 (t, $J = 7.6$ Hz, 1H), 7.26 (t, $J = 7.5$ Hz, 1H), 7.22–7.17 (m, 3H), 6.98 (s, 1H), 5.49 (d, $J = 14.0$ Hz, 2H), 3.90 (s, 3H), 3.61–3.55 (m, 4H), 3.47 (s, 2H), 2.39 (s, 4H); $^{13}$C NMR (100 MHz, DMSO-$d_6$) $\delta$ (ppm): 158.77, 151.31, 150.77, 148.78, 148.35, 146.08, 145.22, 135.35, 134.50, 133.50, 132.20, 129.80, 129.38, 127.83, 127.14, 123.39, 122.20, 121.20, 117.28, 116.10, 115.73, 66.68, 62.40, 56.60, 53.56, 53.21; HRMS (ESI-MS) *m/z*: 532.1745 $[M+H]^+$.

### 3.2.12. 4–(2-Chlorobenzyl)-1-[2-hydroxy-3-methoxy-5-(thiomorpholinomethyl)phenyl] -[1,2,4]triazolo[4,3-a]quinazolin-5(4H)-one (12e)

Beige solid; yield: 69.3%; m.p.:128–131 °C; $^{1}$H NMR (400 MHz, DMSO-$d_6$) $\delta$ (ppm): 9.43 (s, 1H), 8.26 (d, $J = 8.9$ Hz, 1H), 7.73 (t, $J = 8.7$ Hz, 1H), 7.56 (t, $J = 7.4$ Hz, 2H), 7.34 (t, $J = 7.6$ Hz, 1H), 7.26 (t, $J = 7.7$ Hz, 1H), 7.23–7.15 (m, 3H), 6.97 (s, 1H), 5.49 (d, $J = 13.9$ Hz, 2H), 3.90 (s, 3H), 3.51 (s, 2H), 2.71–2.63 (m, 4H), 2.63–2.56 (m, 4H); HRMS (ESI-MS) *m/z*: 548.1515 $[M+H]^+$.

### 3.2.13. 4–(2-Chlorobenzyl)-1-[2-hydroxy-3-methoxy-5-(piperidin-1-ylmethyl)phenyl]-[1,2,4] triazolo[4,3-a]quinazolin-5(4H)-one (12f)

White solid; yield: 82.8%; m.p.:169–171 °C; $^{1}$H NMR (400 MHz, DMSO-$d_6$) $\delta$ (ppm): 9.43 (s, 1H), 8.27 (d, $J = 7.4$ Hz, 1H), 7.73 (t, $J = 7.1$ Hz, 1H), 7.55 (d, $J = 6.9$ Hz, 2H), 7.34 (t, $J = 6.9$ Hz, 1H), 7.29 − 7.17 (m, 4H), 6.96 (s, 1H), 5.50 (d, $J = 10.6$ Hz, 2H), 3.90 (s,

3H), 3.43 (s, 2H), 2.35 (s, 4H), 1.49 (s, 4H), 1.39 (s, 2H); $^{13}$C NMR (100 MHz, DMSO-$d_6$) $\delta$ (ppm): 158.78, 154.84, 148.73, 148.30, 146.12, 145.05, 135.31, 134.51, 133.46, 132.19, 130.69, 129.80, 129.53, 129.37, 127.83, 127.13, 123.16, 117.29, 116.03, 115.71, 115.47, 62.74, 56.58, 54.28, 44.32, 26.04, 24.51; HRMS (ESI-MS) *m/z*: 530.1948 $[M+H]^+$.

### *3.2.14. 4–(2-Chlorobenzyl)-1-{2-hydroxy-3-methoxy-5-[(4-methylpiperidin-1-yl)methyl] phenyl}- [1,2,4]triazolo[4,3-a]quinazolin-5(4H)-one (12 g)*

Brown solid; yield: 72.2%; m.p.:125–128 °C; $^1$H NMR (400 MHz, DMSO-$d_6$) $\delta$ (ppm): 9.42 (s, 1H), 8.26 (s, 1H), 7.73 (s, 1H), 7.56 (s, 2H), 7.34 (s, 1H), 7.21 (dd, $J$ = 17.0, 11.4 Hz, 4H), 6.95 (s, 1H), 5.49 (d, $J$ = 8.5 Hz, 2H), 3.89 (s, 3H), 3.43 (s, 2H), 2.81 (s, 2H), 1.91 (t, $J$ = 11.3 Hz, 2H), 1.56 (d, $J$ = 10.1 Hz, 2H), 1.31 (s, 1H), 1.12 (s, 2H), 0.88 (s, 3H); $^{13}$C NMR (100 MHz, DMSO-$d_6$) $\delta$ (ppm): 158.78, 154.16, 148.73, 148.32, 148.02, 146.12, 145.06, 135.31, 134.52, 133.46, 132.20, 130.80, 129.81, 129.53, 129.37, 127.83, 127.13, 123.13, 117.30, 116.04, 115.68, 56.60, 53.68, 50.58, 44.25, 34.47, 30.79, 22.23; HRMS (ESI-MS) *m/z*: 544.2103 $[M+H]^+$.

### *3.2.15. 4–(2-Chlorobenzyl)-1-{2-hydroxy-5-[(4-hydroxypiperidin-1-yl) methyl]-3-methoxyphenyl}- [1,2,4] triazolo[4,3-a]quinazolin-5(4H)-one (12 h)*

Light yellow solid; yield: 57.9%; m.p.:141–143 °C; $^1$H NMR (400 MHz, DMSO-$d_6$) $\delta$ (ppm): 9.40 (s, 1H), 8.27 (d, $J$ = 7.9 Hz, 1H), 7.73 (t, $J$ = 8.3 Hz, 1H), 7.57 (t, $J$ = 7.2 Hz, 2H), 7.35 (t, $J$ = 8.0 Hz, 1H), 7.29–7.17 (m, 4H), 6.95 (s, 1H), 5.49 (d, $J$ = 13.0 Hz, 2H), 4.55 (d, $J$ = 3.9 Hz, 1H), 3.90 (s, 3H), 3.44 (s, 2H), 2.74–2.67 (m, 2H), 2.05 (t, $J$ = 10.3 Hz, 2H), 1.75–1.67 (m, 2H), 1.43–1.33 (m, 2H); HRMS (ESI-MS) *m/z*: 546.1895 $[M+H]^+$.

### *3.2.16. 1-{3-[4–(2-Chlorobenzyl)-5-oxo-4,5-dihydro-[1,2,4]triazolo[4,3-a] quinazolin-1-yl]-4- hydroxy-5- methoxybenzyl}piperidine-4-carboxylic acid (12i)*

Off-white solid; yield: 80.5%; m.p.:187–190 °C; $^1$H NMR (400 MHz, DMSO-$d_6$) $\delta$ (ppm): 9.47 (s, 1H), 8.26 (d, $J$ = 7.8 Hz, 1H), 7.73 (t, $J$ = 7.6 Hz, 1H), 7.56 (t, $J$ = 6.9 Hz, 2H), 7.34 (t, $J$ = 7.2 Hz, 1H), 7.28–7.17 (m, 4H), 6.96 (s, 1H), 5.49 (d, $J$ = 13.6 Hz, 2H), 3.90 (s, 3H), 3.45 (s, 2H), 2.79 (d, $J$ = 9.1 Hz, 2H), 2.18 (t, $J$ = 10.9 Hz, 1H), 1.99 (t, $J$ = 10.8 Hz, 2H), 1.77 (d, $J$ = 11.5 Hz, 2H), 1.54 (s, 2H); HRMS (ESI-MS) *m/z*: 574.1842 $[M+H]^+$.

### *3.2.17. 4–(2-Chlorobenzyl)-1-{2-hydroxy-3-methoxy-5-[(4-methylpiperazin-1-yl)methyl] phenyl}- [1,2,4]triazolo[4,3-a]quinazolin-5(4H)-one (12j)*

Beige solid; yield: 65.9%; m.p.:215–218 °C; $^1$H NMR (400 MHz, DMSO-$d_6$) $\delta$ (ppm): 9.44 (s, 1H), 8.27 (d, $J$ = 7.0 Hz, 1H), 7.73 (t, $J$ = 7.3 Hz, 1H), 7.55 (d, $J$ = 7.0 Hz, 2H), 7.39–7.31 (m, 1H), 7.30–7.13 (m, 4H), 6.97 (s, 1H), 5.50 (d, $J$ = 12.5 Hz, 2H), 3.90 (s, 3H), 3.46 (s, 2H), 2.39 (s, 4H), 2.33 (s, 4H), 2.14 (s, 3H); $^{13}$C NMR (100 MHz, DMSO-$d_6$) $\delta$ (ppm): 158.79, 154.76, 148.75, 148.34, 146.11, 145.15, 135.34, 134.52, 133.47, 132.21, 130.36, 129.82, 129.54, 129.39, 127.85, 123.25, 120.43, 117.30, 116.10, 115.74, 115.52, 58.59, 56.59, 55.22, 52.95, 46.17, 44.34; HRMS (ESI-MS) *m/z*: 545.2054 $[M+H]^+$.

### *3.2.18. Tert-Butyl 4-{3-[4–(2-chlorobenzyl)-5-oxo-4,5-dihydro-[1,2,4] triazolo[4,3-a] quinazolin-1-yl]-4-hydroxy-5-methoxybenzyl}piperazine-1-carboxylate (12k)*

White solid; yield: 61.0%; m.p.:130–133 °C; $^1$H NMR (400 MHz, DMSO-$d_6$) $\delta$ (ppm): 9.45 (s, 1H), 8.27 (d, $J$ = 7.8 Hz, 1H), 7.74 (t, $J$ = 7.9 Hz, 1H), 7.57 (t, $J$ = 7.6 Hz, 2H), 7.34 (t, $J$ = 7.0 Hz, 1H), 7.26 (t, $J$ = 7.5 Hz, 1H), 7.20 (t, $J$ = 7.4 Hz, 3H), 6.98 (s, 1H), 5.50 (d, $J$ = 14.0 Hz, 2H), 3.91 (s, 3H), 3.50 (s, 2H), 3.32 (s, 4H), 2.38–2.32 (m, 4H), 1.39 (s, 9H); $^{13}$C NMR (100 MHz, DMSO-$d_6$) $\delta$ (ppm): 159.39, 158.81, 148.72, 148.39, 145.27, 144.07, 139.35, 137.10, 135.42, 134.51, 133.47, 132.24, 129.91, 129.83, 129.40, 127.85, 127.79, 126.76, 126.49, 123.53, 117.29, 116.74, 79.19, 56.60, 52.73, 49.90, 44.34, 43.66, 28.53; HRMS (ESI-MS) *m/z*: 631.2422 $[M+H]^+$.

### *3.2.19. 4–(2-Chlorobenzyl)-1-[5-(piperazin-1-ylmethyl)-2-hydroxy-3-methoxyphenyl]-[1,2,4] triazolo[4,3-a]quinazoline-5(4H)-one hydrochloride (12 l)*

Off-white solid; yiled: 82.2%; m.p.:223–226 °C; $^1$H NMR (400 MHz, $D_2O$) $\delta$ (ppm): 8.10 (d, $J$ = 7.6 Hz, 1H), 7.53–7.48 (m, 1H), 7.45–7.38 (m, 2H), 7.35 (s, 1H), 7.17 (q, $J$ = 8.7 Hz, 3H), 7.05 (t, $J$ = 7.5 Hz, 1H), 6.99 (d, $J$ = 7.6 Hz, 1H), 5.37 (s, 2H), 4.32 (s, 2H), 3.89 (s, 3H), 3.48 (s, 8H); $^{13}$C NMR (100 MHz, DMSO-$d_6$) $\delta$ (ppm): 158.74, 153.90, 148.84, 148.51, 145.61, 135.68, 134.33, 133.59, 133.41, 132.22, 129.84, 129.54, 129.40, 127.84, 127.79, 127.26, 125.21, 120.63, 117.22, 116.34, 115.98, 63.36, 56.76, 56.03, 44.70, 44.36; HRMS (ESI-MS) *m/z*: 531.1902 $[M+H]^+$.

### *3.2.20. 4–(2-Chlorobenzyl)-1–(2-hydroxy-5-{[4–(2-hydroxyethyl)piperazin-1-yl] methyl}-3- methoxyphenyl)-[1,2,4]triazolo[4,3-a]quinazolin-5(4H)-one (12 m)*

Light yellow solid; yield: 54.1%; m.p.:134–137 °C; $^1$H NMR (400 MHz, $CDCl_3$) $\delta$ (ppm): 8.41 (d, $J$ = 7.9 Hz, 1H), 7.55 (t, $J$ = 8.3 Hz, 1H), 7.48 (t, $J$ = 7.4 Hz, 1H), 7.42 (d, $J$ = 7.8 Hz, 1H), 7.36 (d, $J$ = 8.3 Hz, 1H), 7.19 (dt, $J$ = 14.9, 4.8 Hz, 3H), 7.13 (d, $J$ = 2.9 Hz, 2H), 5.74 (s, 2H), 3.98 (s, 3H), 3.61 (t, $J$ = 5.3 Hz, 2H), 3.51 (s, 2H), 2.65–2.41 (m, 10H); HRMS (ESI-MS) *m/z*: 575.2162 $[M+H]^+$.

### *3.2.21. 4–(2-Chlorobenzyl)-1-{3-[(dimethylamino)methyl]-4-hydroxy-5-methoxyphenyl} -[1,2,4] triazolo[4,3-a]quinazolin-5(4H)-one (14a)*

White solid; yield: 80.5%; m.p.:117–120 °C; $^1$H NMR (400 MHz, DMSO-$d_6$) $\delta$ (ppm): 8.26 (d, $J$ = 7.9 Hz, 1H), 7.72 (t, $J$ = 8.6 Hz, 1H), 7.55 (dt, $J$ = 7.2, 3.4 Hz, 2H), 7.36–7.32 (m, 1H), 7.26–7.17 (m, 4H), 7.04 (s, 1H), 5.47 (s, 2H), 3.78 (s, 3H), 3.66 (s, 2H), 2.28 (s, 6H); $^{13}$C NMR (100 MHz, DMSO-$d_6$) $\delta$ (ppm): 158.84, 153.57, 149.09, 148.85, 148.64, 148.22, 134.98, 134.63, 133.40, 132.17, 129.77, 129.71, 129.36, 127.83, 127.76, 127.07, 124.39, 123.15, 118.36, 117.66, 116.23, 60.22, 56.40, 53.69, 44.74; HRMS (ESI-MS) *m/z*: 490.1632 $[M+H]^+$.

### *3.2.22. 4–(2-Chlorobenzyl)-1-{3-[(diethylamino)methyl]-4-hydroxy-5-methoxyphenyl}-[1,2,4] triazolo[4,3-a]quinazolin-5(4H)-one (14 b)*

Brown solid; yield: 74.2%; m.p.:100–103 °C; $^1$H NMR (400 MHz, DMSO-$d_6$) $\delta$ (ppm): 8.27 (s, 1H), 7.71 (s, 1H), 7.55 (s, 2H), 7.36–7.14 (m, 5H), 7.03 (s, 1H), 5.47 (s, 2H), 3.83 (s, 2H), 3.76 (s, 3H), 2.60 (s, 4H), 1.04 (s, 6H); HRMS (ESI-MS) *m/z*: 518.1946 $[M+H]^+$.

### 3.2.23. 4–(2-Chlorobenzyl)-1-[4-hydroxy-3-methoxy-5-(pyrrolidin-1-ylmethyl)phenyl]-[1,2,4] triazolo[4,3-a]quinazolin-5(4H)-one (14c)

Brown solid; yield: 79.8%; m.p.:118–120 °C; $^1$H NMR (400 MHz, DMSO-$d_6$) $\delta$ (ppm): 8.26 (d, $J = 9.3$ Hz, 1H), 7.72 (t, $J = 8.7$ Hz, 1H), 7.58–7.53 (m, 2H), 7.36–7.31 (m, 1H), 7.27–7.20 (m, 3H), 7.16 (s, 1H), 7.06 (s, 1H), 5.47 (s, 2H), 3.82 (s, 2H), 3.77 (s, 3H), 2.58 (s, 4H), 1.76 (s, 4H); HRMS (ESI-MS) *m/z*: 516.1792 $[M + H]^+$.

### 3.2.24. 4–(2-Chlorobenzyl)-1-[4-hydroxy-3-methoxy-5-(morpholinomethyl)phenyl]-[1,2,4]triazolo [4,3-a]quinazolin-5(4H)-one (14d)

Beige solid; yield: 82.1%; m.p.:206–208 °C; $^1$H NMR (400 MHz, DMSO-$d_6$) $\delta$ (ppm): 8.27 (d, $J = 7.6$ Hz, 1H), 7.71 (t, $J = 7.7$ Hz, 1H), 7.59–7.52 (m, 2H), 7.34 (t, $J = 6.7$ Hz, 1H), 7.22 (dd, $J = 13.6$, 4.8 Hz, 4H), 7.11 (s, 1H), 5.48 (s, 2H), 3.80 (s, 3H), 3.68 (s, 2H), 3.60 (s, 4H), 2.48 (s, 4H); $^{13}$C NMR (100 MHz, DMSO-$d_6$) $\delta$ (ppm): 158.87, 153.61, 148.85, 148.69, 148.36, 148.25, 135.00, 134.63, 133.42, 132.19, 129.78, 129.37, 128.32, 127.88, 127.77, 127.05, 123.98, 123.67, 118.65, 117.69, 116.28, 66.62, 58.30, 56.48, 53.25, 44.22; HRMS (ESI-MS) *m/z*: 532.1741 $[M + H]^+$.

### 3.2.25. 4–(2-Chlorobenzyl)-1-[4-hydroxy-3-methoxy-5-(thiomorpholinomethyl)phenyl] -[1,2,4]triazolo[4,3-a]quinazolin-5(4H)-one (14e)

Light brown solid; yield: 63.8%; m.p.:230–232 °C; $^1$H NMR (400 MHz, DMSO-$d_6$) $\delta$ (ppm): 8.26 (d, $J = 7.3$ Hz, 1H), 7.72 (t, $J = 7.2$ Hz, 1H), 7.55 (d, $J = 6.8$ Hz, 2H), 7.33 (d, $J = 6.4$ Hz, 1H), 7.28–7.17 (m, 4H), 7.09 (s, 1H), 5.47 (s, 2H), 3.79 (s, 3H), 3.70 (s, 2H), 2.74 (s, 4H), 2.62 (s, 4H); HRMS (ESI-MS) *m/z*: 548.1510 $[M + H]^+$.

### 3.2.26. 4–(2-Chlorobenzyl)-1-[4-hydroxy-3-methoxy-5-(piperidin-1-ylmethyl)phenyl]-[1,2,4] triazolo[4,3-a]quinazolin-5(4H)-one (14f)

Off-white solid; yield: 63.9%; m.p.:115–117 °C; $^1$H NMR (400 MHz, DMSO-$d_6$) $\delta$ (ppm): 8.26 (d, $J = 6.5$ Hz, 1H), 7.72 (t, $J = 7.9$ Hz, 1H), 7.59–7.53 (m, 2H), 7.34 (t, $J = 7.2$ Hz, 1H), 7.24 (q, $J = 8.0$ Hz, 3H), 7.16 (s, 1H), 7.02 (s, 1H), 5.47 (s, 2H), 3.76 (s, 3H), 3.71 (s, 2H), 2.49 (s, 4H), 1.59–1.50 (m, 4H), 1.47–1.40 (m, 2H); HRMS (ESI-MS) *m/z*: 530.1947 $[M + H]^+$.

### 3.2.27. 4–(2-Chlorobenzyl)-1-{4-hydroxy-3-methoxy-5-[(4-methylpiperidin-1-yl)methyl] phenyl}-[1,2,4]triazolo[4,3-a]quinazolin-5(4H)-one (14 g)

White solid; yield: 77.8%; m.p.:120–122 °C; $^1$H NMR (400 MHz, DMSO-$d_6$) $\delta$ (ppm): 8.26 (d, $J = 8.7$ Hz, 1H), 7.72 (t, $J = 7.9$ Hz, 1H), 7.58–7.53 (m, 2H), 7.34 (t, $J = 8.4$ Hz, 1H), 7.24 (q, $J = 8.2$ Hz, 3H), 7.16 (s, 1H), 7.02 (s, 1H), 5.47 (s, 2H), 3.76 (s, 3H), 3.72 (s, 2H), 2.92 (d, $J = 10.8$ Hz, 2H), 2.09 (t, $J = 11.5$ Hz, 2H), 1.65 (d, $J = 13.6$ Hz, 2H), 1.46–1.35 (m, 1H), 1.20–1.09 (m, 2H), 0.91 (d, $J = 6.4$ Hz, 3H); HRMS (ESI-MS) *m/z*: 544.2101 $[M + H]^+$.

### 3.2.28. 4–(2-Chlorobenzyl)-1-{4-hydroxy-3-[(4-hydroxypiperidin-1-yl) methyl]-5-methoxyphenyl}-[1,2,4]triazolo[4,3-a]quinazolin-5(4H)-one (14 h)

White solid; yield: 69.8%; m.p.:134–136 °C; $^1$H NMR (400 MHz, DMSO-$d_6$) $\delta$ (ppm): 8.27 (d, $J = 9.3$ Hz, 1H), 7.72 (t, $J = 7.9$ Hz, 1H), 7.59–7.53 (m, 2H), 7.37–7.32 (m, 1H), 7.25 (q, $J = 7.9$ Hz, 3H), 7.17 (s, 1H), 7.04 (s, 1H), 5.48 (s, 2H), 3.78 (s, 3H), 3.72 (s, 2H), 3.58–3.51 (m, 1H), 2.85–2.74 (m, 2H), 2.25 (t, $J = 9.6$ Hz, 2H), 1.81–1.72 (m, 2H), 1.43 (q, $J = 10.5$, 8.9 Hz, 2H); HRMS (ESI-MS) *m/z*: 546.1896 $[M + H]^+$.

### 3.2.29. 1-{5-[4–(2-Chlorobenzyl)-5-oxo-4,5-dihydro-[1,2,4]triazolo[4,3-a]quinazolin-1-yl]-2- hydroxy-3-methoxybenzyl}piperidine-4-carboxylic acid (14i)

Off-white solid; yield: 73.7%; m.p.:183–185 °C; $^1$H NMR (400 MHz, DMSO-$d_6$) $\delta$ (ppm): 8.28–8.24 (m, 1H), 7.72 (t, $J = 7.9$ Hz, 1H), 7.56 (dd, $J = 7.6$, 4.8 Hz, 2H), 7.34 (t, $J = 7.1$ Hz, 1H), 7.24 (q, $J = 9.0$, 8.4 Hz, 3H), 7.17 (s, 1H), 7.04 (s, 1H), 5.47 (s, 2H), 3.77 (s, 3H), 3.70 (s, 2H), 2.88 (d, $J = 9.8$ Hz, 2H), 2.23 (t, $J = 11.2$ Hz, 1H), 2.16 (t, $J = 11.4$ Hz, 2H), 1.87–1.79 (m, 2H), 1.56 (q, $J = 12.2$ Hz, 2H); HRMS (ESI-MS) *m/z*: 574.1845 $[M + H]^+$.

### 3.2.30. 4–(2-Chlorobenzyl)-1-{4-hydroxy-3-methoxy-5-[(4-methylpiperazin-1-yl)methyl] phenyl}-[1,2,4]triazolo[4,3-a]quinazolin-5(4H)-one (14j)

Light brown solid; yield: 75.4%; m.p.:123–125 °C; $^1$H NMR (400 MHz, $CDCl_3$) $\delta$ (ppm): 8.42 (d, $J = 7.1$ Hz, 1H), 7.51 (p, $J = 7.2$ Hz, 2H), 7.42 (d, $J = 7.7$ Hz, 1H), 7.32 (d, $J = 8.0$ Hz, 1H), 7.23–7.19 (m, 1H), 7.16 (d, $J = 6.5$ Hz, 2H), 7.05 (s, 1H), 6.95 (s, 1H), 5.72 (s, 2H), 3.88 (s, 3H), 3.82 (s, 2H), 2.62 (s, 8H), 2.33 (s, 3H); $^{13}$C NMR (100 MHz, DMSO-$d_6$) $\delta$ (ppm): 158.84, 148.83, 148.75, 148.67, 148.19, 134.99, 134.61, 133.40, 132.17, 129.77, 129.70, 129.36, 127.85, 127.75, 127.06, 123.90, 123.33, 118.53, 117.66, 116.26, 112.89, 58.53, 56.41, 55.05, 52.57, 46.05, 44.22; HRMS (ESI-MS) *m/z*: 545.2056 $[M + H]^+$.

### 3.2.31. Tert-Butyl4-{5-[4–(2-chlorobenzyl)-5-oxo-4,5-dihydro-[1,2,4]triazolo[4,3-a] quinazolin-1-yl]-2-hydroxy-3-methoxybenzyl}piperazine-1-carboxylate (14k)

Light brown solid; yield: 67.8%; m.p.:127–130 °C; $^1$H NMR (400 MHz, DMSO-$d_6$) $\delta$ (ppm): 8.27 (d, $J = 8.9$ Hz, 1H), 7.72 (t, $J = 7.9$ Hz, 1H), 7.56 (t, $J = 7.3$ Hz, 2H), 7.34 (t, $J = 8.5$ Hz, 1H), 7.28–7.19 (m, 4H), 7.10 (s, 1H), 5.47 (s, 2H), 3.80 (s, 3H), 3.67 (s, 2H), 3.43–3.34 (m, 4H), 2.46–2.39 (m, 4H), 1.39 (s, 9H); $^{13}$C NMR (100 MHz, DMSO-$d_6$) $\delta$ (ppm): 159.01, 158.86, 154.22, 148.83, 148.68, 148.22, 148.18, 135.00, 134.62, 133.40, 132.17, 129.77, 129.72, 129.37, 127.85, 127.76, 124.19, 123.74, 123.47, 118.65, 117.69, 116.32, 79.33, 57.63, 56.44, 52.60, 44.20, 43.26, 28.52; HRMS (ESI-MS) *m/z*: 631.2422 $[M + H]^+$.

### 3.2.32. 4–(2-Chlorobenzyl)-1-[3-(piperazin-1-ylmethyl)-4-hydroxy-5-methoxyphenyl]-[1,2,4]triazolo[4,3-a]quinazoline-5(4H)-one hydrochloride (14 l)

Light brown solid; yield: 71.1%; m.p.:214–216 °C; $^1$H NMR (400 MHz, $D_2O$) $\delta$ (ppm): 8.12 (d, $J = 9.4$ Hz, 1H), 7.51–7.43 (m, 2H), 7.40 (d, $J = 8.0$ Hz, 1H), 7.25 (s, 1H), 7.23–7.13 (m, 3H), 7.08 (t, $J = 8.0$ Hz, 1H), 6.98 (d, $J = 7.0$ Hz, 1H), 5.36 (s, 2H), 4.40 (s, 2H), 3.78 (s, 3H), 3.50 (s, 8H); HRMS (ESI-MS) *m/z*: 531.1897 $[M + H]^+$.

### 3.2.33. 4–(2-Chlorobenzyl)-1–(4-hydroxy-3-{[4–(2-hydroxyethyl)piperazin-1-yl]methyl} -5- methoxyphenyl)-[1,2,4]triazolo[4,3-a]quinazolin-5(4H)-one (14 m)

Light yellow solid; yield: 82.7%; m.p.:117–120 °C; $^1$H NMR (400 MHz, $CDCl_3$) $\delta$ (ppm): 8.43 (d, $J = 9.1$ Hz, 1H), 7.51 (p, $J = 7.4$ Hz, 2H), 7.43 (d, $J = 7.8$ Hz, 1H), 7.32 (d, $J = 8.3$ Hz, 1H), 7.24–7.19 (m, 1H), 7.16 (d, $J = 6.5$ Hz, 2H), 7.05 (s, 1H), 6.95 (s, 1H),

5.73 (s, 2H), 3.88 (s, 3H), 3.83 (s, 2H), 3.64 (t, $J = 5.3$ Hz, 2H), 2.89–2.42 (m, 10H); $^{13}$C NMR (100 MHz, DMSO-$d_6$) $\delta$ (ppm): 158.85, 154.42, 148.83, 148.78, 148.67, 148.19, 135.02, 134.62, 133.40, 132.16, 129.77, 129.71, 129.36, 127.84, 127.77, 127.06, 123.84, 123.31, 118.50, 117.66, 116.27, 61.60, 60.57, 58.99, 56.40, 53.51, 52.68, 44.23; HRMS (ESI-MS) *m/z*: 575.2161 $[M+H]^+$.

### *3.2.34. General procedure for preparation of the intermediate compounds 16a–16j*

2-Bromo-4-fluorobenzaldehyde (**15**) (9.8 mmol), corresponding secondary amine (11.82 mmol), and potassium carbonate (14.78 mmol) were successively added to a solution of DMF (20 ml). After stirring at 85 °C for 1 h. The reaction solution was poured into water (50 ml), whereby a precipitate was formed. The precipitant was filtered, washed with water and *N*-hexane twice, and dried to afford the corresponding compound **16a–16j**.

### *3.2.35. 2-Bromo-4-morpholinobenzaldehyde (16a)*

White solid; yield: 82.2%; $^1$H NMR (400 MHz, DMSO-$d_6$) $\delta$ 9.94 (d, $J = 0.7$ Hz, 1H), 7.69 (d, $J = 8.9$ Hz, 1H), 7.16 (d, $J = 2.5$ Hz, 1H), 7.04 (dd, $J = 8.9$, 2.5 Hz, 1H), 3.74–3.66 (m, 4H), 3.41–3.33 (m, 4H).

### *3.2.36. 2-Bromo-4–(4-methylpiperidin-1-yl)benzaldehyde (16 b)*

Yellow solid; yield: 74.0%; $^1$H NMR (400 MHz, $CDCl_3$) $\delta$ 10.01 (s, 1H) 8.01 (s, 1H), 7.76 (d, $J = 8.9$ Hz, 1H), 6.95 (d, $J = 2.6$ Hz, 1H), 6.79 (ddd, $J = 9.0$, 2.6, 0.8 Hz, 1H), 3.93–3.82 (m, 2H), 2.98–2.84 (m, 2H), 2.88–2.75 (m, 2H), (s, 1H), 1.80–1.65 (m, 2H), 1.69–1.59 (m, 1H), 1.31–1.16 (m, 2H), 0.97 (d, $J = 6.5$ Hz, 3H)

### *3.2.37. 2-Bromo-4-(pyrrolidin-1-yl)benzaldehyde (16c)*

White solid; yield: 78.3%; $^1$H NMR (400 MHz, $CDCl_3$) $\delta$ 10.09 (s, 1H) 7.81 (d, $J = 8.8$ Hz, 1H), 6.70 (d, $J = 2.4$ Hz, 1H), 6.52 (ddd, $J = 8.8$, 2.4, 0.8 Hz, 1H), 3.44–3.34 (m, 4H), 2.14–2.02 (m, 4H)

### *3.2.38. 2-Bromo-4–(4-hydroxypiperidin-1-yl)benzaldehyde (16d)*

White solid; yield: 81.1%; $^1$H NMR (400 MHz, $CDCl_3$) $\delta$ 10.09 (d, $J = 0.8$ Hz, 1H), 7.80 (d, $J = 8.9$ Hz, 1H), 7.00 (d, $J = 2.6$ Hz, 1H), 6.87–6.79 (m, 1H), 3.99 (dt, $J = 8.3$, 4.2 Hz, 1H), 3.76 (ddd, $J = 13.2$, 6.9, 4.3 Hz, 2H), 3.21 (ddd, $J = 13.0$, 9.1, 3.4 Hz, 2H), 2.07–1.94 (m, 2H), 1.66 (ddd, $J = 12.9$, 8.5, 4.0 Hz, 2H).

### *3.2.39. 2-Bromo-4-(dipropylamino)benzaldehyde (16e)*

White solid; yield: 85.0%; $^1$H NMR (400 MHz, $CDCl_3$) $\delta$ 10.04 (s, 1H), 7.76 (d, $J = 8.9$ Hz, 1H), 6.73 (d, $J = 2.5$ Hz, 1H), 6.57 (ddd, $J = 9.0$, 2.6, 0.8 Hz, 1H), 3.34–3.25 (m, 4H), 1.64 (h, $J = 7.4$ Hz, 4H), 0.96 (t, $J = 7.4$ Hz, 6H).

### *3.2.40. 2-Bromo-4-(diethylamino)benzaldehyde (16f)*

White solid; yield: 72.0%; $^1$H NMR (400 MHz, $CDCl_3$) $\delta$ 10.05 (s, 1H) 7.78 (d, $J = 8.9$ Hz, 1H), 6.76 (d, $J = 2.5$ Hz, 1H), 6.61 (dd, $J = 9.0$, 2.5 Hz, 1H), 3.42 (q, $J = 7.1$ Hz, 4H), 1.22 (t, $J = 7.1$ Hz, 6H).

### *3.2.41. 2-Bromo-4-thiomorpholinobenzaldehyde (16 g)*

White solid; yield: 80.0%; $^1$H NMR (400 MHz, $CDCl_3$) $\delta$ 10.13 (s, 1H), 7.83 (d, $J = 8.9$ Hz, 1H), 7.00 (d, $J = 2.4$ Hz, 1H), 6.84 (dd, $J = 8.9$, 2.5 Hz, 1H), 3.89–3.81 (m, 4H), 3.39–3.31 (m, 4H).

### *3.2.42. 2-Bromo-4-(dibutylamino)benzaldehyde (16 h)*

White solid; yield: 69.2%; $^1$H NMR (400 MHz, $CDCl_3$) $\delta$ 10.05 (s, 1H) 7.77 (d, $J = 9.0$ Hz, 1H), 6.73 (d, $J = 2.6$ Hz, 1H), 6.57 (ddd, $J = 9.0$, 2.6, 0.8 Hz, 1H), 3.36–3.28 (m, 4H), 1.65–1.53 (m, 4H), 1.37 (h, $J = 7.3$ Hz, 4H), 0.98 (t, $J = 7.3$ Hz, 6H).

### *3.2.43. 2-Bromo-4-(dimethylamino)benzaldehyde (16i)*

White solid; yield: 73.3%; $^1$H NMR (400 MHz, $CDCl_3$) $\delta$ 10.09 (s, 1H) 7.80 (d, $J = 8.9$ Hz, 1H), 6.79 (d, $J = 2.5$ Hz, 1H), 6.63 (dd, $J = 9.0$, 2.5 Hz, 1H), 3.08 (s, 6H).

### *3.2.44. 2-Bromo-4–(2-methylpiperidin-1-yl)benzaldehyde (16j)*

White solid; yield: 77.1%; $^1$H NMR (400 MHz, $CDCl_3$) $\delta$ 10.04 (s, 1H), 7.78 (d, $J = 8.9$ Hz, 2H), 6.96 (d, $J = 2.5$ Hz, 2H), 6.79 (d, $J = 9.0$, 2.6, 0.8 Hz, 2H), 3.87 (d, 2H), 2.92 (t, $J = 7.4$ Hz, 2H), 1.75 (d, 2H), 1.65 (s, 1H), 1.30–1.23 (m, 2H), 0.96 (t, $J = 7.4$ Hz, 3H).

### *3.2.45. General procedure for preparation of the targeting compounds 17a–17j*

To the solution of **10** (1.00 g, 3.33 mmol) in isopropanol (20 ml) was added intermediate **16a–16j** (0.90 g, 3.33 mmol) and glacial acetic acid (0.02 g, 0.33 mmol), the resulting reaction mixture was heated to 50 °C for 20 min. After cooling, the ferric chloride hexahydrate (4.49 g, 16.63 mmol) was added under stirring and the reaction mixture was heated for 30 min at 90 °C. After the reaction is complete, it was quenched by adding water, solid was precipitated. The solid was filtered, washed with water, ethyl acetate and acetone twice, and dried to afford the targeting compound **17a–17j**.

### *3.2.46. 1–(2-Bromo-4-morpholinophenyl)-4–(2-chlorobenzyl)-[1,2,4]triazolo[4,3-a]quinazolin-5(4H) -one (17a)*

Off-white solid; yield: 75.7%; m.p.:238–240 °C; $^1$H NMR (400 MHz, DMSO-$d_6$) $\delta$ 8.29 (dd, $J = 7.8$, 1.6 Hz, 1H), 7.81–7.71 (m, 1H), 7.64–7.47 (m, 3H), 7.42–7.14 (m, 5H), 7.02 (d, $J = 8.4$ Hz, 1H), 5.50 (q, $J = 16.4$ Hz, 2H), 3.77 (t, $J = 4.8$ Hz, 4H), 3.32 (t, $J = 4.8$ Hz, 4H); $^{13}$C NMR (100 MHz, DMSO-$d_6$) $\delta$ (ppm): 158.71, 153.99, 148.60, 147.37, 136.18, 135.63, 134.25, 133.43, 133.39, 132.24, 129.84, 129.42, 127.87, 127.84, 127.37, 125.42, 121.66, 118.70, 118.00, 117.36, 114.05, 66.31, 47.51, 44.33; ESI-MS, *m/z*: 552.06 $[M+H]^+$.

### *3.2.47. 1-[2-Bromo-4–(4-methylpiperidin-1-yl)phenyl]-4–(2-chlorobenzyl)-[1,2,4]triazolo[4,3-a] quinazolin-5(4H)-one (17 b)*

Purple solid; yield: 83.3%; m.p.:257–259 °C; $^1$H NMR (400 MHz, DMSO-$d_6$) $\delta$ 8.29 (d, $J = 7.8$ Hz, 1H), 7.79 (t, $J = 8.0$ Hz, 1H), 7.58 (dd, $J = 16.4$, 7.9 Hz, 2H), 7.45 (d, $J = 8.6$ Hz, 1H), 7.39–7.12 (m, 5H), 7.06 (d, $J = 8.4$ Hz, 1H), 5.59–5.41 (m, 2H), 3.94 (d, $J = 12.9$ Hz, 2H), 2.87 (t, $J = 12.3$ Hz, 2H), 1.73 (d, $J = 12.8$ Hz, 2H), 1.22 (q, $J = 12.8$, 12.4 Hz, 2H), 0.97 (d, $J = 6.5$ Hz, 3H); ESI-MS, *m/z*: 564.10 $[M+H]^+$.

### *3.2.48. 1-[2-Bromo-4-(pyrrolidin-1-yl)phenyl]-4–(2-chlorobenzyl)-[1,2,4] triazolo[4,3-a]quinazolin- 5(4H)-one (17c)*

Dark green solid; yield: 62.0%; m.p.:229–232 °C; $^1$H NMR (400 MHz, DMSO-$d_6$) $\delta$ 8.28 (d, $J = 7.5$ Hz, 1H), 7.78 (t, $J = 7.8$ Hz, 1H), 7.64–7.48 (m, 2H), 7.43 (d, $J = 8.3$ Hz, 1H), 7.34 (t, $J = 7.3$ Hz, 1H), 7.22 (dd, $J = 19.4$, 7.4 Hz, 2H), 7.08 (d, $J = 8.2$ Hz, 1H), 6.96 (s, 1H), 6.75 (d, $J = 8.4$ Hz, 1H), 5.50 (q, $J = 16.3$ Hz, 2H), 3.37 (d, $J = 5.8$ Hz, 4H), 2.01 (d, $J = 5.7$ Hz, 4H); $^{13}$C NMR (100 MHz, DMSO-$d_6$) $\delta$ (ppm):

158.73, 153.54, 150.45, 148.50, 147.86, 135.58, 135.31, 134.36, 133.41, 133.32, 132.23, 129.83, 129.41, 127.84, 127.30, 125.97, 125.25, 117.34, 115.28, 114.89, 111.65, 47.85, 44.36, 25.46; ESI-MS, *m/z*:: 536.07 $[M+H]^+$.

### 3.2.49. 1-[2-Bromo-4–(4-hydroxypiperidin-1-yl)phenyl]-4–(2-chlorobenzyl)-[1,2,4] triazolo[4,3-a]quinazolin-5(4H)-one (17d)

Beige solid; yield: 72.2%; m.p.:214–217 °C; $^1$H NMR (400 MHz, DMSO-$d_6$) $\delta$ 8.28 (d, $J=7.9$ Hz, 1H), 7.74 (t, $J=7.9$ Hz, 1H), 7.63–7.50 (m, 2H), 7.45 (d, $J=8.6$ Hz, 1H), 7.34 (d, $J=7.5$ Hz, 2H), 7.26 (d, $J=8.0$ Hz, 1H), 7.15 (t, $J=7.5$ Hz, 2H), 7.04 (d, $J=8.5$ Hz, 1H), 5.49 (q, $J=16.3$ Hz, 2H), 3.74 (d, $J=12.3$ Hz, 3H), 3.07 (t, $J=11.4$ Hz, 2H), 1.87–1.81 (m, 2H), 1.47 (s, 2H); $^{13}$C NMR (100 MHz, DMSO-$d_6$) $\delta$ (ppm): 158.72, 153.54, 148.93, 148.56, 147.52, 135.60, 134.28, 133.43, 133.40, 132.24, 130.35, 129.83, 129.41, 127.87, 127.84, 127.34, 125.45, 117.97, 117.36, 115.28, 114.18, 66.08, 45.37, 44.31, 33.85; ESI-MS, *m/z*: 566.08 $[M+H]^+$.

### 3.2.50. 1-[2-Bromo-4-(dipropylamino)phenyl]-4–(2-chlorobenzyl)-[1,2,4]triazolo[4,3-a]quinazolin-5 (4H)-one (17e)

Pink solid; yield: 47.3%; m.p.:183–186 °C; $^1$H NMR (400 MHz, DMSO-$d_6$) $\delta$ 8.27 (d, $J=7.2$ Hz, 1H), 7.75 (s, 1H), 7.61–7.50 (m, 2H), 7.42–7.31 (m, 2H), 7.25 (s, 1H), 7.16 (s, 1H), 7.09 (d, $J=7.9$ Hz, 1H), 7.00 (s, 1H), 6.85 (s, 1H), 5.51 (t, $J=16.1$ Hz, 2H), 3.33 (s, 4H), 1.59 (s, 4H), 0.92 (s, 6H); $^{13}$C NMR (100 MHz, DMSO-$d_6$) $\delta$ (ppm): 158.74, 151.19, 148.50, 147.79, 136.55, 135.58, 134.36, 133.42, 132.24, 129.84, 129.41, 128.38, 127.85, 127.30, 126.93, 126.23, 125.50, 117.35, 115.30, 114.76, 111.37, 52.21, 44.30, 20.28, 11.58; ESI-MS, *m/z*: 566.11 $[M+H]^+$.

### 3.2.51. 1-[2-Bromo-4-(diethylamino)phenyl]-4–(2-chlorobenzyl)-[1,2,4]triazolo[4,3-a] quinazolin-5 (4H)-one (17f)

White solid; yield: 41.9%; m.p.:229–232 °C; $^1$H NMR (400 MHz, DMSO-$d_6$) $\delta$ 8.32–8.25 (m, 1H), 7.80 (s, 1H), 7.57 (s, 2H), 7.41 (s, 1H), 7.35 (s, 1H), 7.26 (s, 2H), 7.13 (s, 2H), 7.06 (s, 1H), 6.89 (s, 1H), 5.51 (d, $J=18.2$ Hz, 2H), 3.46 (s, 4H), 1.17 (s, 6H); $^{13}$C NMR (100 MHz, DMSO-$d_6$) $\delta$ (ppm): 158.85, 150.80, 148.60, 147.89, 147.14, 135.69, 134.46, 133.75, 133.61, 133.53, 132.34, 129.90, 129.51, 128.12, 127.96, 127.39, 125.68, 117.45, 115.42, 114.92, 114.76, 44.39, 42.70, 12.77; ESI-MS, *m/z*: 538.08 $[M+H]^+$.

### 3.2.52. 1–(2-Bromo-4-thiomorpholinophenyl)-4–(2-chlorobenzyl)-[1,2,4]triazolo[4,3-a] quinazolin-5 (4H)-one (17 g)

White solid; yield: 35.8%; m.p.:248–250 °C; $^1$H NMR (400 MHz, DMSO-$d_6$) $\delta$ 8.29 (d, $J=7.9$ Hz, 1H), 7.79 (s, 1H), 7.57 (d, $J=12.8$ Hz, 2H), 7.47 (s, 1H), 7.37 (d, $J=18.1$ Hz, 2H), 7.24 (d, $J=17.6$ Hz, 2H), 7.16 (s, 1H), 7.07 (s, 1H), 5.55 (d, $J=15.7$ Hz, 1H), 5.46 (d, $J=16.4$ Hz, 1H), 3.834 (m, 4H), 2.70 (s, 4H); ESI-MS, *m/z*: 658.04 $[M+H]^+$.

### 3.2.53. 1-[2-Bromo-4-(dibutylamino)phenyl]-4–(2-chlorobenzyl)-[1,2,4]triazolo[4,3-a] quinazolin-5 (4H)-one (17 h)

White solid; yield: 59.6%; m.p.:176–179 °C; $^1$H NMR (400 MHz, DMSO-$d_6$) $\delta$ 8.29 (s, 1H), 7.79 (s, 1H), 7.57 (s, 2H), 7.35 (s, 1H), 7.22 (s, 3H), 7.03 (s, 1H), 6.86 (s, 1H), 5.50 (s, 2H), 1.57 (s, 4H), 1.37 (s, 6H), 0.96 (s, 8H); $^{13}$C NMR (100 MHz, DMSO-$d_6$) $\delta$ (ppm): 158.74, 151.11, 148.49, 148.31, 147.77, 135.82, 135.56, 134.34, 133.41, 132.23, 129.83, 129.80, 129.40, 127.85, 127.30, 125.79, 125.49, 117.34, 115.28, 114.72, 111.34, 50.26, 44.30, 29.21, 20.08, 14.28; ESI-MS, *m/z*: 594.14 $[M+H]^+$.

### 3.2.54. 1-[2-Bromo-4-(dimethylamino)phenyl]-4–(2-chlorobenzyl)-[1,2,4]triazolo[4,3-a]quinazolin- 5(4H)-one (17i)

White solid; yield: 43.6%; m.p.:246–249 °C; $^1$H NMR (400 MHz, DMSO-$d_6$) $\delta$ 8.29 (d, $J=7.9$ Hz, 1H), 7.78 (s, 1H), 7.58 (dd, $J=15.3$, 7.7 Hz, 2H), 7.45 (d, $J=8.7$ Hz, 1H), 7.35 (t, $J=7.7$ Hz, 1H), 7.31–7.17 (m, 2H), 7.15–7.02 (m, 2H), 6.92 (d, $J=7.9$ Hz, 1H), 5.55 (d, $J=16.4$ Hz, 1H), 5.46 (d, $J=16.4$ Hz, 1H), 3.06 (s, 6H); $^{13}$C NMR (100 MHz, DMSO-$d_6$) $\delta$ (ppm): 158.73, 153.12, 148.53, 147.73, 146.50, 135.58, 135.08, 134.34, 133.41, 133.24, 133.16, 132.24, 129.84, 129.41, 127.85, 127.31, 125.33, 117.35, 115.61, 115.29, 111.72, 41.13, 39.19; ESI-MS, *m/z*: 510.05 $[M+H]^+$.

### 3.2.55. 1-[2-Bromo-4–(2-methylpiperidin-1-yl)phenyl]-4–(2-chlorobenzyl)-[1,2,4] triazolo[4,3-a] quinazolin-5(4H)-one (17j)

White solid; yield: 76.2%; m.p.:245–248 °C; $^1$H NMR (400 MHz, DMSO-$d_6$) $\delta$ 8.29 (d, $J=7.8$ Hz, 1H), 7.79 (s, 1H), 7.64–7.53 (m, 2H), 7.45 (d, $J=8.7$ Hz, 1H), 7.35 (s, 1H), 7.32–7.19 (m, 3H), 7.15–7.04 (m, 2H), 5.50 (q, $J=16.5$ Hz, 2H), 4.35 (s, 1H), 3.67–3.62 (d, 1H) 2.96 (s, 1H), 1.78 (s, 2H), 1.60 (d, $J=15.5$ Hz, 4H), 1.12 (d, $J=6.5$ Hz, 3H); $^{13}$C NMR (100 MHz, DMSO-$d_6$) $\delta$ (ppm): 158.73, 153.43, 148.54, 147.58, 146.70, 135.60, 134.30, 133.57, 133.40, 132.23, 129.83, 129.41, 127.85, 127.34, 127.09, 125.54, 124.81, 117.35, 116.87, 115.28, 113.91, 49.10, 44.31, 41.56, 30.61, 25.63, 18.42, 13.52; ESI-MS, *m/z*: 564.10 $[M+H]^+$.

## 3.3. Pharmacology

### 3.3.1. In vitro enzyme inhibition assay

The enzyme level activity of target compounds was evaluated using self-isolated and purified SHP2 as the target protein, and **SHP244** was employed as a positive control. The concentration of the target compound was 10 μM. Briefly, target compounds (5 μL) and **SHP244** (5 μL) were transferred to the test plate, with 2 replicate wells for each group; then, 0.5 nM SHP2 protein (5 μL) was added and incubated for 10 min. Next, 0.5 μM 2 P-IRS-1 peptides (5 μL) was added, and the reaction was carried out for 30 min. Finally, 6,8-difluoro-4-methylumbelliferyl phosphate (DiFMUP) was added, and the reactions were performed for 30 min at 25 °C, the reactions were stopped by adding 5 μL of diluted 160 μM bpV. The plate was read using Envision (Perkin Elmer) at 340 nm and 450 nm. For each experiment, the average of two replicate wells was considered the result. The inhibition rate (%) was calculated using the following equation:

$$\%\text{Inhibition} = (\text{ODvalueofnormalhole} - \text{ODvalueofdosinghole}) / \text{ODvalueofnormalhole} \times 100$$

### 3.3.2. MTT assay in vitro

Many studies have shown that SHP2 levels are frequently elevated in melanoma. Inhibition of SHP2 activity, could effectively block SHP2-mediated activation of ERK1/2 and AKT, and reduce viability, migration, and colony formation of melanoma cells, thereby suppressing the growth of tumour cells[4,27,28]. Therefore, human melanoma cells (A375) were selected to assess the anti-proliferative activity *in vitro* of compounds **12f**, **12l**, **12j**, **17e,** and **17f** by employing the standard MTT assay *in vitro*. SHP244 was used as a positive control and incubated for 72 h. Based on experimental data, the drug concentration that can inhibit the growth of tumour cells by half, i.e. the $IC_{50}$ value, was calculated using Prism software (GraphPad Software, Inc., La Jolla, CA, USA).

#### 3.3.3. Metabolic stability of liver microsomes assay of compound 12l in vitro

The metabolic stability of liver microsomes was evaluated for target product **12l** using **SHP244** as the positive control. Standard stock solution (100 μM) of test compounds were prepared in dimethyl sulfoxide (DMSO) and acetonitrile (ACN). The compounds (1 μM) were preincubated with microsomes (human microsome: HLM, Corning, lot No. 38296; Rat microsome: RLM, Xenotech, Lot No. 1910100) (0.5 mg/mL) for 10 min at 37 °C in phosphate buffer (pH 7.4). The incubation system was then activated by initiation factors (NADPH, Chem-Impex International, Cat. No. 00616, 1 nM). After incubation for different time points (0, 5, 15, 30, 45, and 60 min) at 37 °C, cold (4 °C) acetonitrile (containing 200 ng/mL tolbutamide and 200 ng/mL labetalol) was added to quench the reaction. All sampling plates were shaken for 10 min and then centrifuged at 4000 rpm for 20 min at 4 °C. The supernatants were diluted three times with HPLC water and analysed using LC-MS/MS[29,30].

$$T_{1/2}\ (\mathrm{min}):\ \ \mathrm{half-life}$$

$$\mathrm{CL}\ (\mu\mathrm{L/min/mg}):\mathrm{intrinsicclearance} = 0.693/T_{1/2}/\mathrm{microsomeproteinpermL(mg)}.$$

#### 3.3.4. In vitro kinetic solubility assessment of compound 12l

The test compounds (**SHP244** and **12l**, 10 mM in DMSO; 10 μL) were added to the lower chambers of Whatman Miniuniprep vials (GE Healthcare Whatman, Cat. No. UN203NPUORG). Followed by the addition of 490 μL of phosphate buffer (50 mM, pH 7.4) and 0.1 N HCl solution, respectively. The resulting mixture was oscillated on a Barnstead shaker for 24 h at room temperature at a speed of 800 rpm. Finally, the mixture was centrifuged for 20 min (4000 rpm) and injected into the HPLC system for analysis.

## 4. Results and discussion

### 4.1. Chemistry

The synthetic routes of target compounds **12a–12m**, **14a–14m,** and **17a–17j** are summarised in Scheme 1. First, the nucleophilic substitution of isatoic anhydride and 2-chlorobenzylamine in the presence of ethyl acetate at 40 °C afforded intermediate **8**[31], followed by the introduction of the disulphide bond by carbon disulphide and potassium hydroxide to obtain **9**[32–35]. Reflux in isopropanol, the nucleophilic addition of compound **9** with 80% $NH_2NH_2{\cdot}H_2O$ to obtain **10**[36,37]. Subsequently, a mixture of **10**, aryl formaldehyde, glacial acetic acid, and isopropanol was heated to 83 °C for 30 min, followed by cooling to room temperature, and the addition of ferric chloride hexahydrate under stirring; then, the mixture was allowed to react at 83 °C for 1.5 h to obtain **11** or **13**[38,39], which reacted with aldehyde and appropriate secondary amines undergoing Mannich-type condensation reaction to afford target compounds **12a–12m** and **14a–14m**, respectively. Subsequently, the substitution of compound **15** with the corresponding secondary amine in the presence of potassium carbonate and *N,N*-dimethylformamide (DMF) afforded **16a–16j**. Finally, target compounds **17a–17j** were successfully obtained via the condensation reaction of intermediate **10** with the corresponding **16a–16j** in the presence of glacial acetic acid, ferric chloride hexahydrate, and isopropanol at 90 °C for 30 min, respectively[40,41].

### 4.2. Biological activity

#### 4.2.1. In vitro enzymatic assay

All synthesised triazoloquinazolinone derivatives were evaluated for their inhibitory activity towards the SHP2 protein enzyme. SHP244 was used as a positive control. The results are expressed as inhibition values and summarised in Table 1. Values represent the average of at least three independent experiments.

As shown in Table 1, all tested compounds displayed inhibitory activities against SHP2 protein enzymatic activity. Compared with **SHP244**, most **12a–12m** compounds demonstrated similar or higher sensitivity to SHP2, thus indicating that the introduction of different secondary amines on the terminal phenyl ring maintained the inhibitory activity. The enzymatic assays revealed that the position of the hydroxyl had a greater impact on activity, which suggested that compounds with the hydroxyl substituent at the 2-position of the phenyl ring exhibited a higher potency than those with the hydroxyl substituent at the 4-position of the phenyl ring. For example, compounds **12a** (33.79% inhibition), **12e** (30.80% inhibition), **12j** (26.20% inhibition), and **12l** (31.84% inhibition) exhibited better inhibitory activity against the SHP2 protein at 10 μM than compounds **14a** (28.13% inhibition), **14e** (25.60% inhibition), **14j** (13.48% inhibition), and **14l** (19.78% inhibition).

A preliminary assessment of structure-activity relationships (SARs) suggested that different biological properties could be observed when various groups were introduced into the phenyl ring. The introduction of electron-donating groups (EDGs) exhibited a positive effect on inhibitory activity. However, compounds with electron-withdrawing groups (EWGs) on the phenyl ring reduced the inhibitory activity. For example, target compounds **12a–12m** (3-methoxy) and **14a–14m** (3-methoxy) exhibited a better effect at 10 μM than compounds **17a–17j** (2-Bromo), indicating that an appropriate electron density on the phenyl ring is probably crucial to enhance the inhibitory activity. Further analysis revealed that compounds with different $NR_1R_2$ groups exhibit different SHP2 inhibitory efficacies; compounds of **12a** ($R_1{=}R_2{=}(CH_3)_2$, 33.79% inhibition), **12d** ($R_1{=}R_2{=}$morpholinyl, 27.56% inhibition), and **12l** ($R_1{=}R_2{=}$piperazine-1-yl hydrochloride, 31.84% inhibition) exhibited higher inhibitory activity than **12f** ($R_1{=}R_2{=}$piperidinyl, 19.19% inhibition), **12g** ($R_1{=}R_2{=}$4-methylpiperidin-1-yl, 17.08% inhibition), and **12i** ($R_1{=}R_2{=}$4-carboxypiperidin-1-yl, 18.90% inhibition) at 10 μM. Moreover, as shown in Table 1, the most promising compound **12l** showed a robust inhibitory effect against SHP2 protein at 10 μM (31.84% inhibition).

However, compared with SHP244, the activity of target compounds was not significantly enhanced, indicating that shortening the distance between the target compound and LYS266 to facilitate the formation of hydrogen bonds is not ideal for improving the inhibitory effect of the compound against SHP2 protein. Therefore, in future investigations, modifying the alternate side chain (chlorophenyl part) on the existing structure could be considered to increase its π-stacking interaction and improve the inhibition efficiency.

In conclusion, given the kinase inhibitory activity of these target compounds, we obtained the following SARs: (a) compounds with a hydroxyl substituent at the 2-position of the phenyl ring presented higher SHP2 protein inhibitory activity than those with a substituent at the 4-position; (b) the presence of EDGs such as

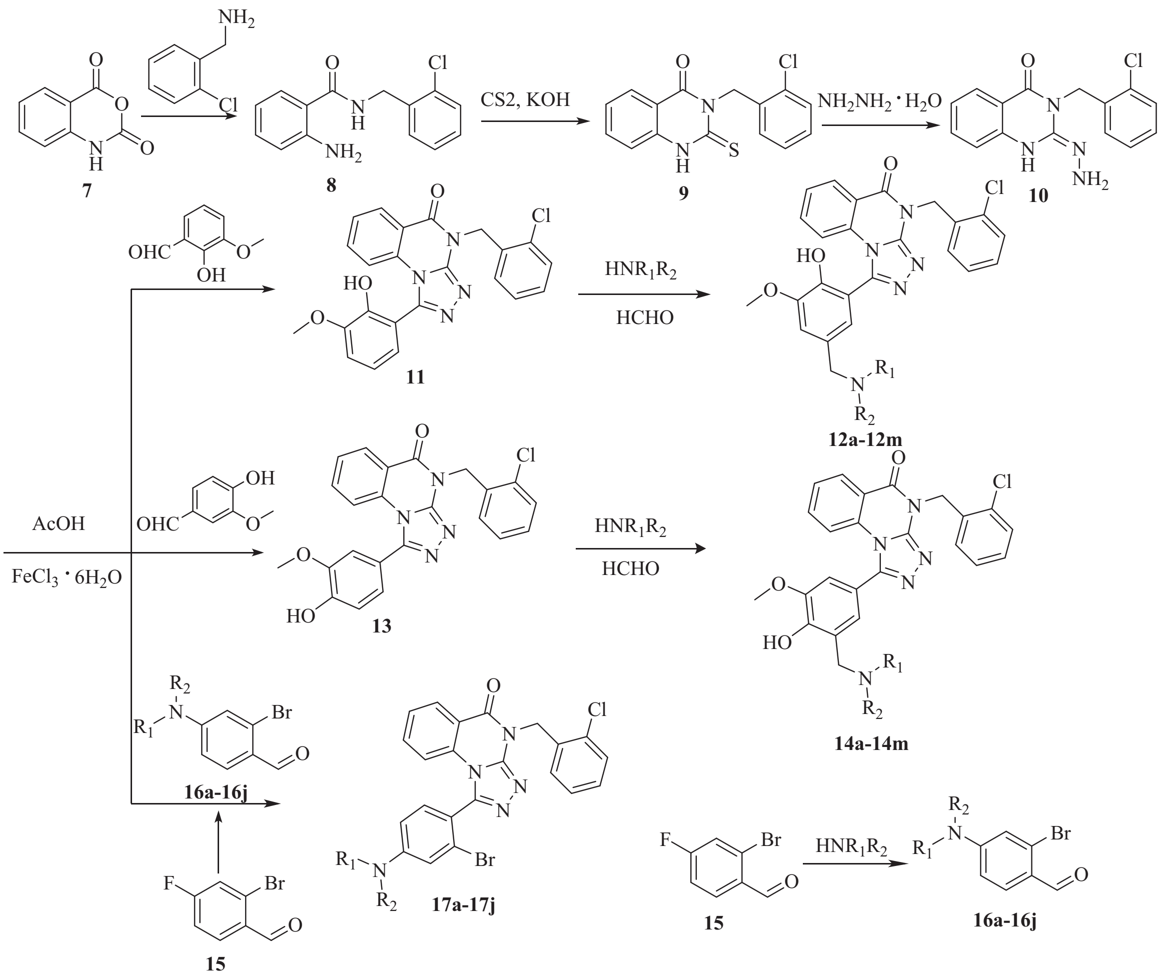

**12a**, **14a**: $R_1$=$R_2$=$(CH_3)_2$
**12b**, **14b**: $R_1$=$R_2$=$(C_2H_5)_2$
**12c**, **14c**: $R_1$=$R_2$=pyrrolldinyl
**12d**, **14d**: $R_1$=$R_2$=morphclinyl
**12e**, **14e**: $R_1$=$R_2$=thiomorpholinyl
**12f**, **14f**: $R_1$=$R_2$=piperidinyl
**12g**, **14g**: $R_1$=$R_2$=4-methylpiperidin-1-yl
**12h**, **14h**: $R_1$=$R_2$=4-hydroxypiperidin-1-yl
**12i**, **14i**: $R_1$=$R_2$=4-carboxypiperidin-1-yl
**12j**, **14j**: $R_1$=$R_2$=4-methylpiperazin-1-yl
**12k**, **14k**: $R_1$=$R_2$=4-(tert-butoxycarbonyl)piperazin-1-yl
**12l**, **14l**: $R_1$=$R_2$=piperazine-1-yl hydrochloride
**12m**, **14m**: $R_1$=$R_2$=4-(2-hydroxyethyl)piperazin-1-yl

**16a**:$R_1$=$R_2$=morphclinyl
**16b**:$R_1$=$R_2$=4-methylpiperidin-1-yl
**16c**:$R_1$=$R_2$=pyrrolldinyl
**16d**:$R_1$=$R_2$=4-hydroxypiperidin-1-yl
**16e**:$R_1$=$R_2$=dipropylamino
**16f**:$R_1$=$R_2$=$(C_2H_5)_2$
**16g**:$R_1$=$R_2$=thiomorpholinyl
**16h**:$R_1$=$R_2$=dibutylamino
**16i**:$R_1$=$R_2$=$(CH_3)_2$
**16j**:$R_1$=$R_2$=2-methylpiperidin-1-yl

**Scheme 1.** Synthetic route of target compounds methoxy groups, exhibited a positive effect on the inhibitory activity; (c) different secondary amine groups ($NR_1R_2$ group) have a certain effect on the inhibitory activity of SHP2 protein kinase.

### *4.2.2. In vitro cytotoxicity*

The cytotoxicity of target compounds was evaluated against the cancer cell line A375 using the MTT assay and **SHP244** as the positive control. The results are summarised in Table 2. We selected five compounds based on their SHP2 inhibitory potential (Table 1) to further explore the antitumor activity, evaluating their efficacy against the cancer cell line A375 (melanoma cells). The results were expressed as $IC_{50}$ values. As shown in Table 2, all selected compounds displayed significant cytotoxicity against A375 when compared with SHP244. Among them, compounds **12l**, **12j**, **17e** revealed excellent activity, presenting $IC_{50}$ values of 14.67 μM, 9.66 μM, 14.7 μM, respectively. These data indicated again that compound **12l** warrants further evaluation as an SHP2 protein inhibitor. Moreover, the piperazine group-containing secondary amine, which was selected to shorten the distance between the target compound and LYS266, conferred the highest efficacy.

### *4.2.3. Metabolic stability of liver microsomes and kinetic solubility assay for compound 12l in vitro*

We next assessed the aqueous solubility of the obtained target compounds. Accordingly, the metabolic stability of liver

**Table 1.** The enzyme inhibitory activities of target compounds on SHP2 protein at 10 μM.

| Compd. | $NR_1R_2$ | % Inhibition[a] 10 μM |
|---|---|---|
| **12a, 14a** |  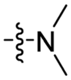 | 33.79, 28.13 |
| **12b, 14b** |  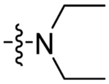 | 25.78, 28.32 |
| **12c, 14c** |  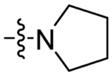 | 24.39, 27.34 |
| **12d, 14d** |  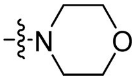 | 27.56, 19.72 |
| **12e, 14e** |  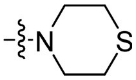 | 30.80, 25.60 |
| **12f, 14f** |  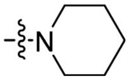 | 19.19, 10.81 |
| **12g, 14g** |  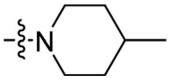 | 17.08, 16.78 |
| **12h, 14h** |  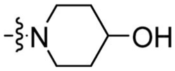 | 21.98, 23.33 |
| **12i, 14i** |  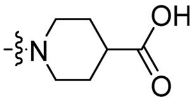 | 18.90, 18.08 |
| **12j, 14j** |  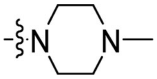 | 26.20, 13.48 |
| **12k, 14k** |  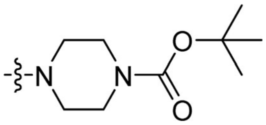 | 23.36, 19.97 |
| **12l, 14l** |  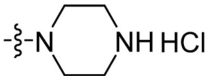 | 31.84, 19.78 |
| **12m, 14m** |  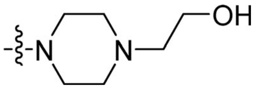 | 25.70, 22.37 |
| **17a** |  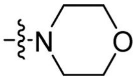 | 17.58 |
| **17b** |  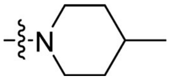 | 10.02 |
| **17c** |  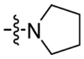 | 24.15 |
| **17d** |  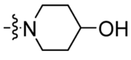 | 24.13 |
| **17e** |  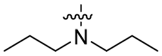 | 16.07 |

(*continued*)

**Table 1.** Continued.

| Compd. | $NR_1R_2$ | % Inhibition[a] 10 μM |
|---|---|---|
| **17f** |  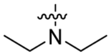 | 11.26 |
| **17g** |  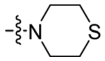 | 9.26 |
| **17h** |  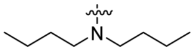 | 12.13 |
| **17i** |  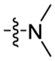 | 13.29 |
| **17j** |  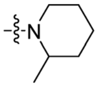 | 10.05 |
| **SHP244**[b] | | 19.67 |

[a]Data presented is the mean ± SD value of three independent determinations.
[b]Used as a positive control.

**Table 2.** *In vitro* cytotoxic activities of the target compounds **12f**, **12l**, **12j**, **17e**, **17f**, and **SHP244** against the A375.

| Compd. | $IC_{50}$ (μM)[a] |
|---|---|
| **12f** | 93.7 |
| **12l** | 14.67 |
| **12j** | 9.66 |
| **17e** | 14.7 |
| **17f** | 61.4 |
| **SHP244**[b] | >100 |

[a]$IC_{50}$ values shown are the mean of duplicate measurements.
[b]Used as a positive control.

**Table 3.** *In vitro* metabolic stability of liver microsomes and kinetic solubility for compounds **12f** and **SHP244**.

| | | Liver microsome stability | | |
|---|---|---|---|---|
| Compd. | Species | $T_{1/2}$(min) | CL (μL/min/mg) | Remaining ($T = 60$min) |
| **12l** | HLM | 75.8 | 18.3 | 57.0% |
| | RLM | >145 | <9.6 | 88.7% |
| **SHP244**[a] | HLM | 14.2 | 97.9 | 4.7% |
| | RLM | 12.2 | 114.0 | 2.9% |
| | | Kinetic solubility (μM) | | |
| **12l** | PB (pH 7.4) | 169 | | |
| | 0.1 N HCl | 191 | | |
| **SHP244**[a] | PB (pH 7.4) | 12.6 | | |
| | 0.1 N HCl | <1.6 | | |

[a]Used as a positive control.

microsomes and kinetic solubility of the most potent compound **12l** was determined. As shown in Table 3, compound **12l** exhibited considerable stability, presenting a clearance rate of 18.3 and <9.6 μL/min/mg in human and rat liver microsomes, respectively. Simultaneously, the $t_{1/2}$ of **12l** on human and rat liver microsomes was 75.8 and >145 min, which is considerably greater than the $t_{1/2}$ of **SHP244** on human and rat liver microsomes (14.2 and 12.2 min, respectively). In addition, the kinetic solubility of **12l** was significantly improved when compared with that of **SHP244** under PB (pH 7.4) and 0.1 N HCl. These results indicated that the hepatic stability and aqueous solubility of compound **12l** were enhanced by introducing a polar functional group.

## 5. Conclusions

In summary, a series of novel triazoloquinazolinone derivatives were designed, synthesised, and evaluated for their biological activity. In addition, the metabolic stability of liver microsomes and kinetic solubility of compound **12l** were examined. Based on our preliminary investigation, the most promising compound **12l** showed a potent inhibitory effect against SHP2 protein at 10 μM (31.84% inhibition) when compared with **SHP244**. Furthermore, **12l** demonstrated superior antitumor activities, presenting an $IC_{50}$ value of 14.67 μM against A375 cells. The SARs revealed that compounds with hydroxyl substituents at 2-position of the phenyl ring exhibited greater activity than compounds with a substituent at the 4-positions. In addition, the presence of EDG such as methoxy groups exhibited a positive effect on inhibitory activity. Moreover, the piperazine group-containing secondary amine, selected to shorten the distance between the target compounds and LYS266 afforded the highest efficacy. Finally, the results of hepatic stability and kinetic solubility revealed that the aqueous solubility of compound **12l** was significantly enhanced by introducing the polar functional group. In conclusion, compound **12l** warrants further assessment as a potential anticancer agent for the treatment of human cancers.

## Author contributions

R.L. Writing – original draft. R.L., D.L., Y.Q. designed and synthesised the compounds, performed the biological evaluation. Z.W. performed the biological evaluation and processed the data. C.Z., D.Y., T.L., Z.Z, Z.H. interpreted the data, reviewed and supervised the manuscript. All authors have read and agreed to the published version of the manuscript.

## Disclosure statement

No potential conflict of interest was reported by the author(s).

## Funding

This work was supported by Guizhou Provincial Natural Science Foundation ([2020]1Y393).
