# Supplementary material for: Design, synthesis, and biological evaluation of novel triazoloquinazolinone derivatives as SHP2 protein inhibitors
Source: J Enzyme Inhib Med Chem. 2021 Nov 8;36(1):2170–82. doi: 10.1080/14756366.2021.1986491 (PMC8583937; doi:10.1080/14756366.2021.1986491)
Supplement: Supplemental Material [file IENZ_A_1986491_SM4220.pdf]

Supplementary Material

Design, synthesis and biological evaluation of novel triazoloquinazolinone derivatives as SHP2 protein inhibitors

Rongshuang Luo[a,b]*, Zhongyuan Wang[c]*, Dali Luo[a,b], Yumei Qin[a,b], Chunshen Zhao[a,b], Di Yang[a,b], Tian Lu[d], Zhixu Zhou[a,b,e] and Zhuyan Huang[a,b]

a. School of Pharmaceutical Sciences, Guizhou University, Guiyang, 550025, China
b. Guizhou Engineering Laboratory for Synthetic Drugs, Guiyang, 550025, China
c. Department of Pharmacy, Guizhou Provincial People's Hospital, Guiyang, 550002, China
d. School of Chinese Materia Medica, Nanjing University of Chinese Medicine, Nanjing, 210023, China
e. Department of Dermatology, Affiliated Hospital of Guizhou Medical University, Guiyang, 550001, China

* These authors contributed equally to this work.

Contents:

Table S1. Summary of target compounds

Figure S1. ^{1}H-NMR spectrum of **8.**

Figure S2. MS spectrum of **8.**

Figure S3. ^{1}H-NMR spectrum of **9.**

Figure S4. MS spectrum of **9.**

Figure S5. ^{1}H-NMR spectrum of **10.**

Figure S6. MS spectrum of **10.**

Figure S7. ^{1}H-NMR spectrum of **11.**

Figure S8. MS spectrum of **11.**

Figure S9. ^{1}H-NMR spectrum of **13.**

Figure S10. MS spectrum of **13.**

Figure S11. ^{1}H-NMR spectrum of **12a.**

Figure S12. MS spectrum of **12a.**

Figure S13.^{13}C-NMR spectrum of **12a.**

Figure S14. ^{1}H-NMR spectrum of **12b.**

Figure S15. MS spectrum of **12b.**

Figure S16. ^{1}H-NMR spectrum of **12c.**

Figure S17. MS spectrum of **12c.**

Figure S18. ^{1}H-NMR spectrum of 1**2d.**

Figure S19. MS spectrum of 1**2d.**

Figure S20.^{13}C-NMR spectrum of **12d.**

Figure S21. ^{1}H-NMR spectrum of 1**2e.**

Figure S22. MS spectrum of **12e.**

Figure S23. ^{1}H-NMR spectrum of 1**2f.**

Figure S24. MS spectrum of 1**2f.**

Figure S25.^{13}C-NMR spectrum of **12f.**

Figure S26. ^{1}H-NMR spectrum of **12g.**

Figure S27. MS spectrum of **12g.**

Figure S28.^{13}C-NMR spectrum of **12g.**

Figure S29. ^{1}H-NMR spectrum of **12h.**

Figure S30. MS spectrum of **12h.**

Figure S31. ^{1}H-NMR spectrum of **12i.**

Figure S32. MS spectrum of **12i.**

Figure S33. ^{1}H-NMR spectrum of **12j.**

Figure S34. MS spectrum of **12j.**

Figure S35.^{13}C-NMR spectrum of **12j.**

Figure S36. ^{1}H-NMR spectrum of **12k.**

Figure S37. MS spectrum of **12k.**

Figure S38.^{13}C-NMR spectrum of **12k.**

Figure S39. ^{1}H-NMR spectrum of **121.**

Figure S40. MS spectrum of **121.**

Figure S41.^{13}C-NMR spectrum of **12l.**

Figure S42. ^{1}H-NMR spectrum of **12m.**

Figure S43. MS spectrum of **12m.**

Figure S44. ^{1}H-NMR spectrum of **14a.**

Figure S45. MS spectrum of **14a.**

Figure S46.^{13}C-NMR spectrum of **14a.**

Figure S47. ^{1}H-NMR spectrum of **14b.**

Figure S48. MS spectrum of **14b.**

Figure S49. ^{1}H-NMR spectrum of **14c.**

Figure S50. MS spectrum of **14c.**

Figure S51. ^{1}H-NMR spectrum of **14d.**

Figure S52. MS spectrum of **14d.**

Figure S53.^{13}C-NMR spectrum of **14d.**

Figure S54. ^{1}H-NMR spectrum of **14e.**

Figure S55. MS spectrum of **14e.**

Figure S56. ^{1}H-NMR spectrum of **14f.**

Figure S57. MS spectrum of **14f.**

Figure S58. ^{1}H-NMR spectrum of **14g.**

Figure S59. MS spectrum of **14g.**

Figure S60. ^{1}H-NMR spectrum of **14h.**

Figure S61. MS spectrum of **14h.**

Figure S62. ^{1}H-NMR spectrum of **14i.**

Figure S63. MS spectrum of **14i.**

Figure S64. ^{1}H-NMR spectrum of **14j.**

Figure S65. MS spectrum of **14j.**

Figure S66.^{13}C-NMR spectrum of **14j.**

Figure S67. ^{1}H-NMR spectrum of **14k.**

Figure S68. MS spectrum of **14k.**

Figure S69.^{13}C-NMR spectrum of **14k.**

Figure S70. ^{1}H-NMR spectrum of **14l.**

Figure S71. MS spectrum of **14l.**

Figure S72. ^{1}H-NMR spectrum of **14m.**

Figure S73. MS spectrum of **14m.**

Figure S74.^{13}C-NMR spectrum of **14m.**

Figure S75. ^{1}H-NMR spectrum of **16a.**

Figure S76. ^{1}H-NMR spectrum of **16b.**

Figure S77. ^{1}H-NMR spectrum of **16c.**

Figure S78. ^{1}H-NMR spectrum of **16d.**

Figure S79. ^{1}H-NMR spectrum of **16e.**

Figure S80. ^{1}H-NMR spectrum of **16f.**

Figure S81. ^{1}H-NMR spectrum of **16g.**

Figure S82. ^{1}H-NMR spectrum of **16h.**

Figure S83. ^{1}H-NMR spectrum of **16i.**

Figure S84. ^{1}H-NMR spectrum of **16j.**

Figure S85. ^{1}H-NMR spectrum of **17a.**

Figure S86. MS spectrum of **17a.**

Figure S87.^{13}C-NMR spectrum of **17a.**

Figure S88. ^{1}H-NMR spectrum of **17b.**

Figure S89. MS spectrum of **17b.**

Figure S90. ^{1}H-NMR spectrum of **17c.**

Figure S91. MS spectrum of **17c.**

Figure S92.^{13}C-NMR spectrum of **17c.**

Figure S93. ^{1}H-NMR spectrum of **17d.**

Figure S94. MS spectrum of **17d.**

Figure S95.^{13}C-NMR spectrum of **17d.**

Figure S96. ^{1}H-NMR spectrum of **17e.**

Figure S97. MS spectrum of **17e.**

Figure S98.^{13}C-NMR spectrum of **17e.**

Figure S99. ^{1}H-NMR spectrum of **17f.**

Figure S100. MS spectrum of **17f.**

Figure S101.^{13}C-NMR spectrum of **17f.**

Figure S102. ^{1}H-NMR spectrum of **17g.**

Figure S103. MS spectrum of **17g.**

Figure S104. ^{1}H-NMR spectrum of **17h.**

Figure S105. MS spectrum of **17h.**

Figure S106.^{13}C-NMR spectrum of **17h.**

Figure S107. ^{1}H-NMR spectrum of **17i.**

Figure S108. MS spectrum of **17i.**

Figure S109.^{13}C-NMR spectrum of **17i.**

Figure S110. ^{1}H-NMR spectrum of **17j.**

Figure S111. MS spectrum of **17j.**

Figure S112.^{13}C-NMR spectrum of **17j.**

Corresponding author. Tel./fax: +86-13639093442; +86-15890031801

E-mail address: zhixuzhou@126.com; 785596957@qq.com

Table S1. Summary of target compounds

Compd	Structural formula	Compd	Structural formula
**12a**	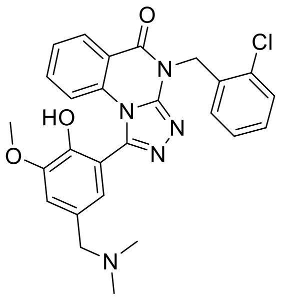	**14a**	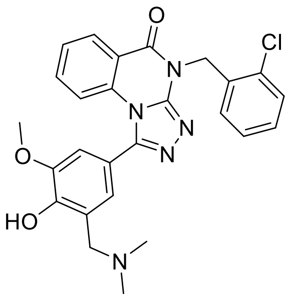
**12b**	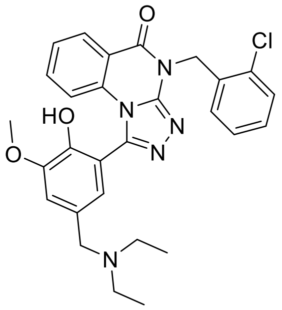	**14b**	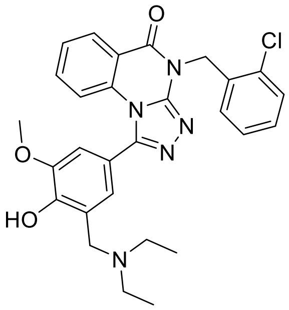
**12c**	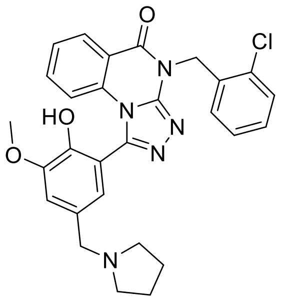	**14c**	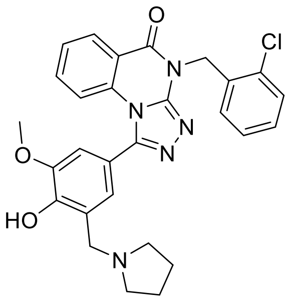
**12d**	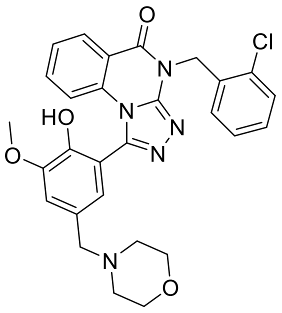	**14d**	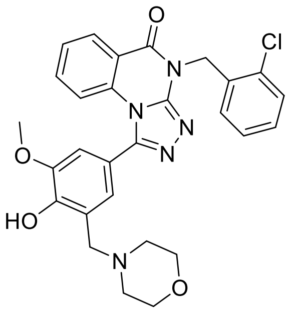
**12e**	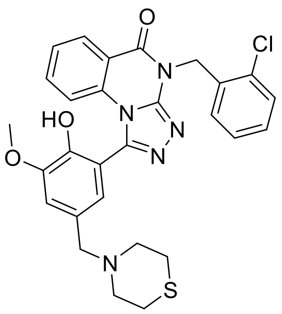	**14e**	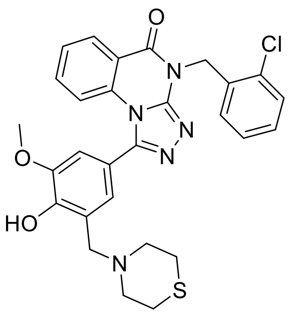

**12f**	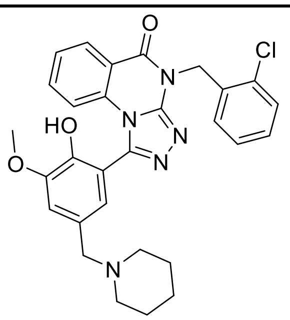	**14f**	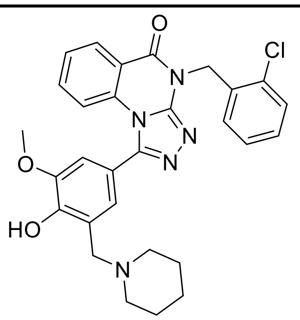
**12g**	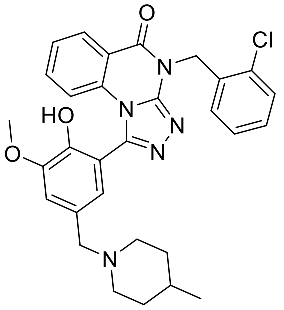	**14g**	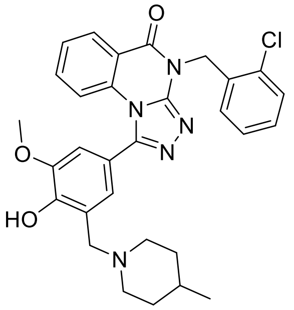
**12h**	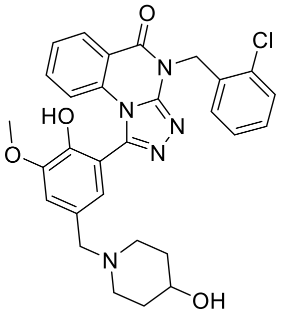	**14h**	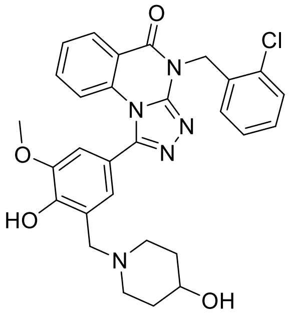
**12i**	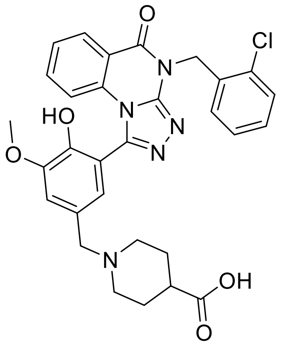	**14i**	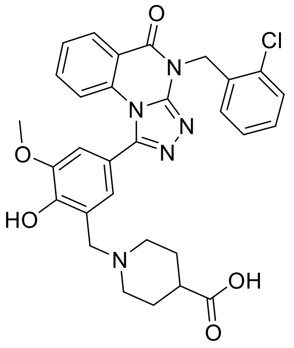
**12j**	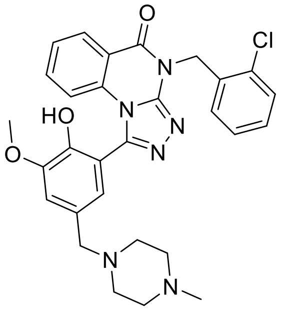	**14j**	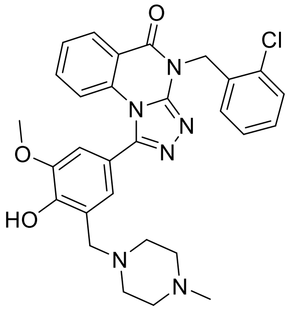

**12k**	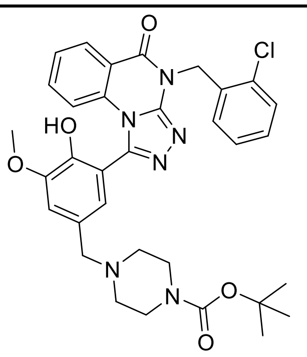	**14k**	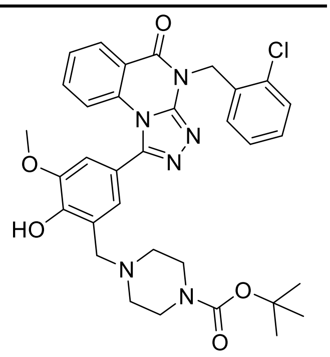
**12l**	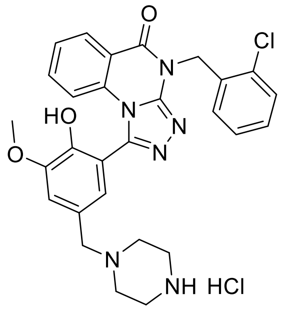	**14l**	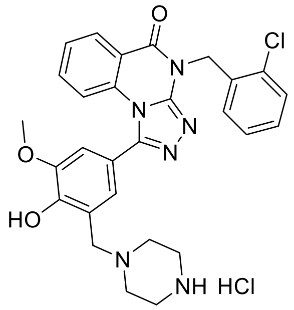
**12m**	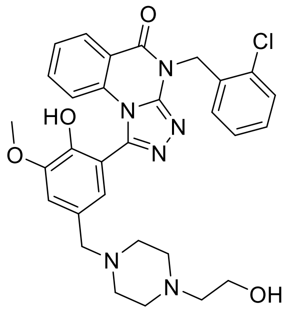	**14m**	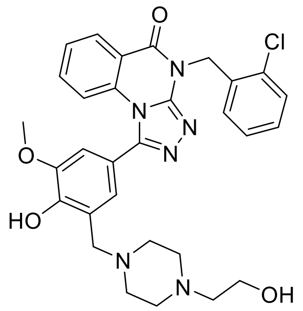
**17a**	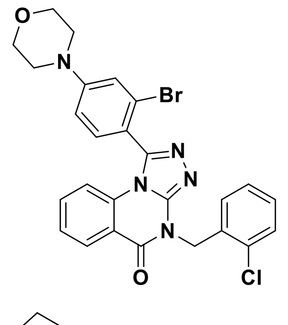	**17b**	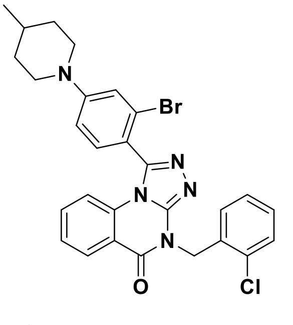
**17c**	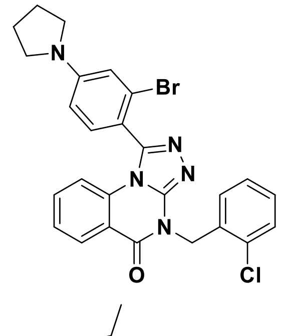	**17d**	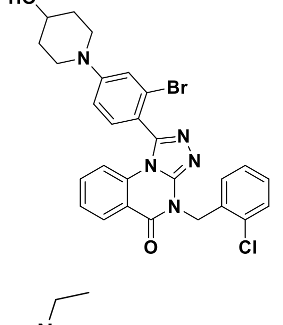
**17e**	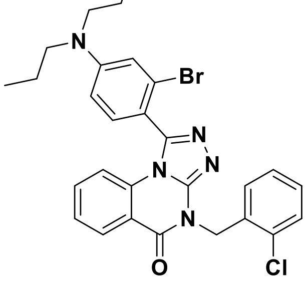	**17f**	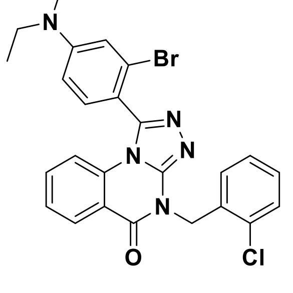

**17g**	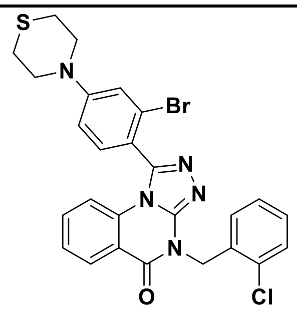	**17h**	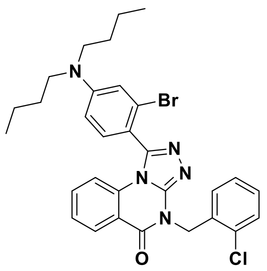
**17i**	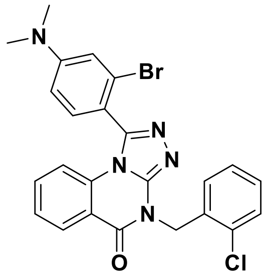	**17j**	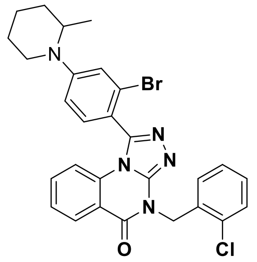

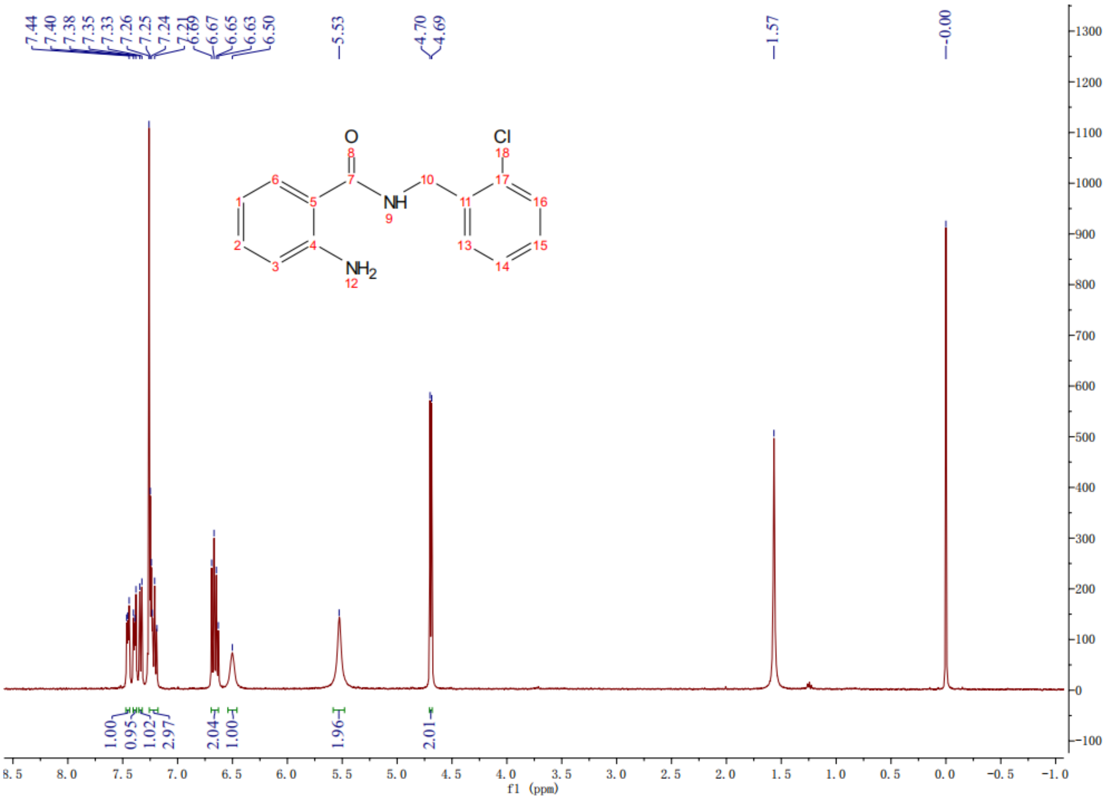

Figure S1. ^{1}H-NMR spectrum of **8.**

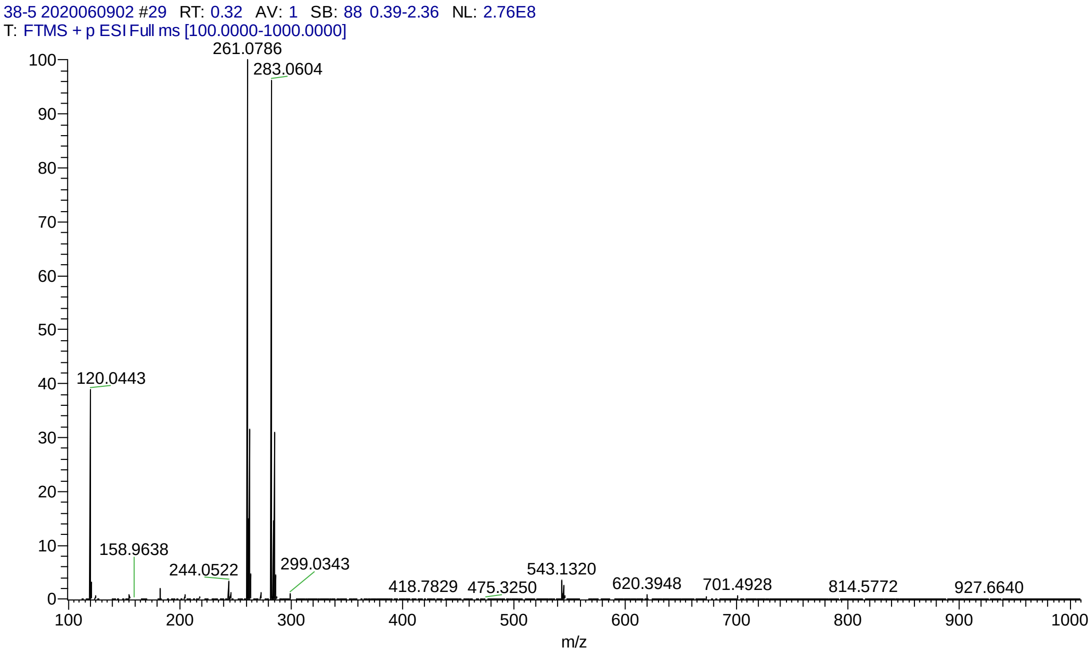

Figure S2. MS spectrum of **8.**

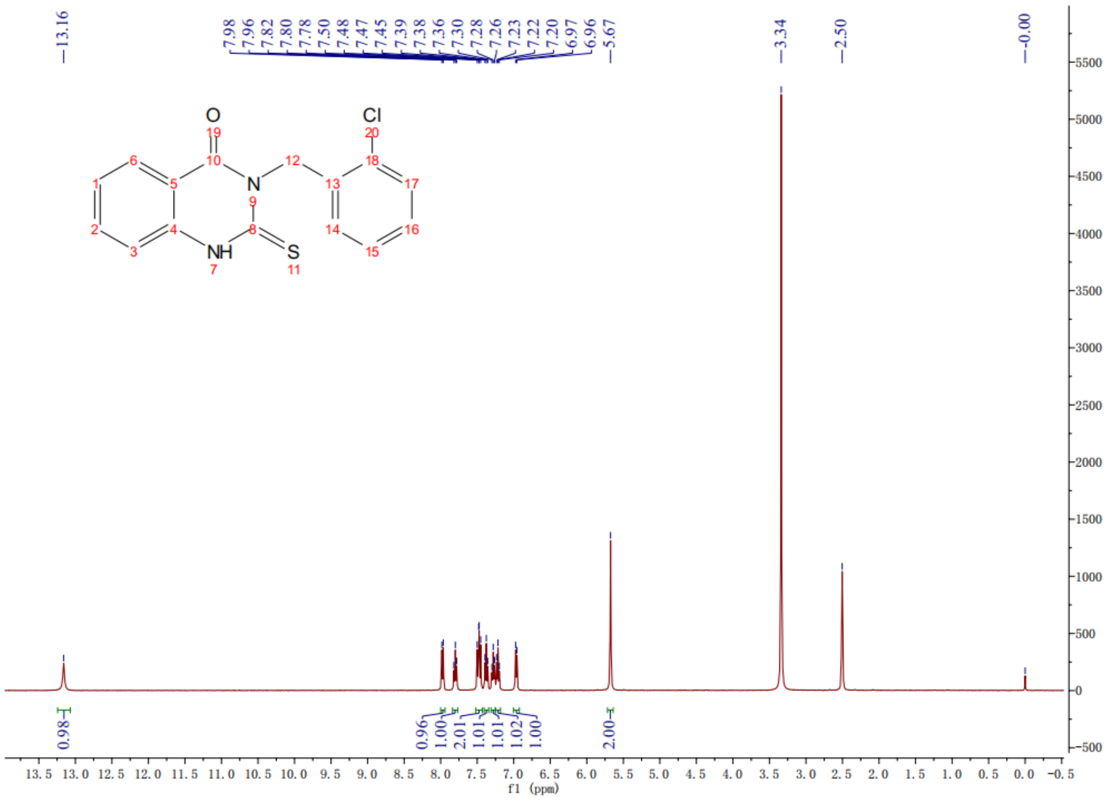

Figure S3. ^{1}H-NMR spectrum of **9**.

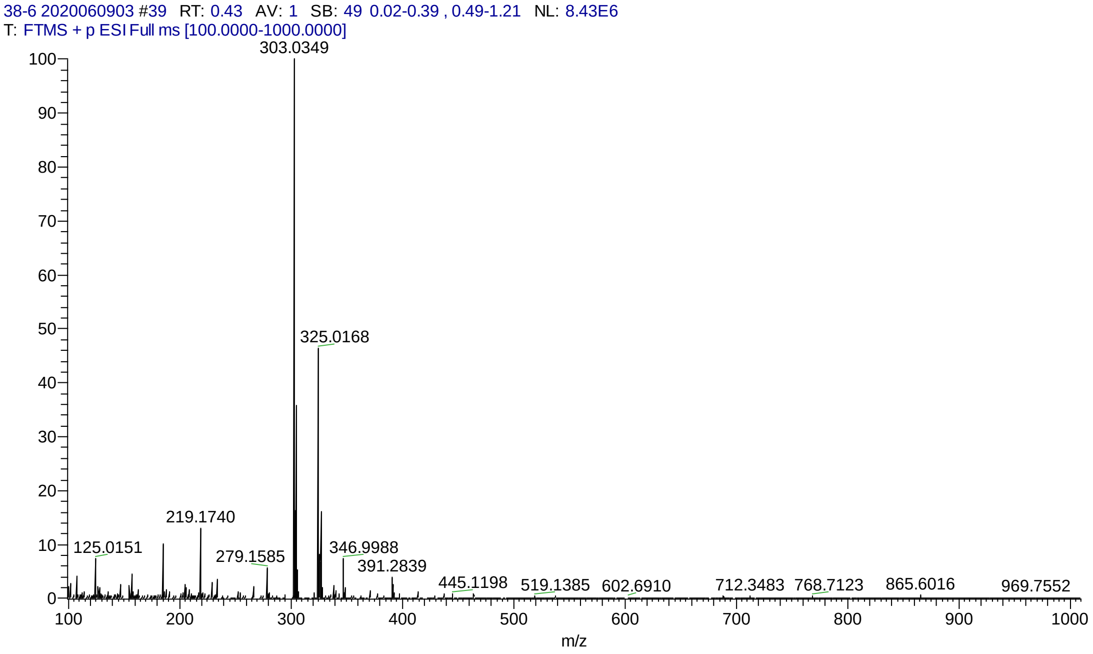

Figure S4. MS spectrum of **9**.

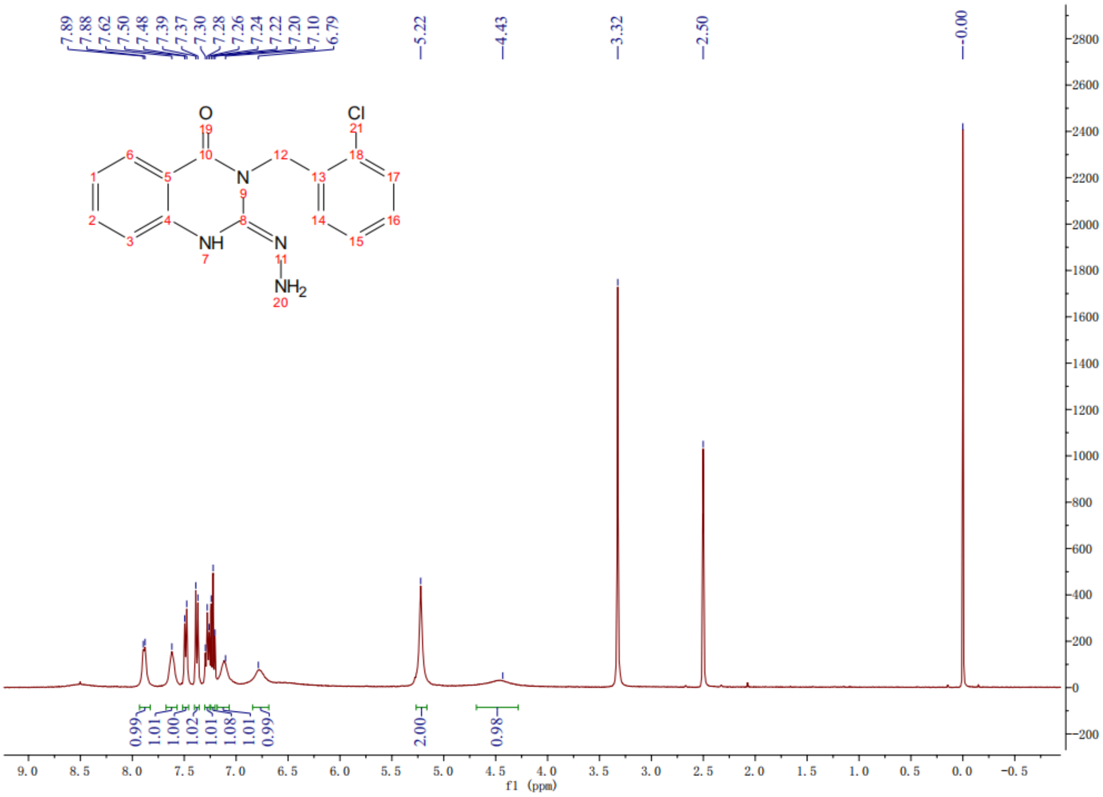

Figure S5. ^{1}H-NMR spectrum of **10**.

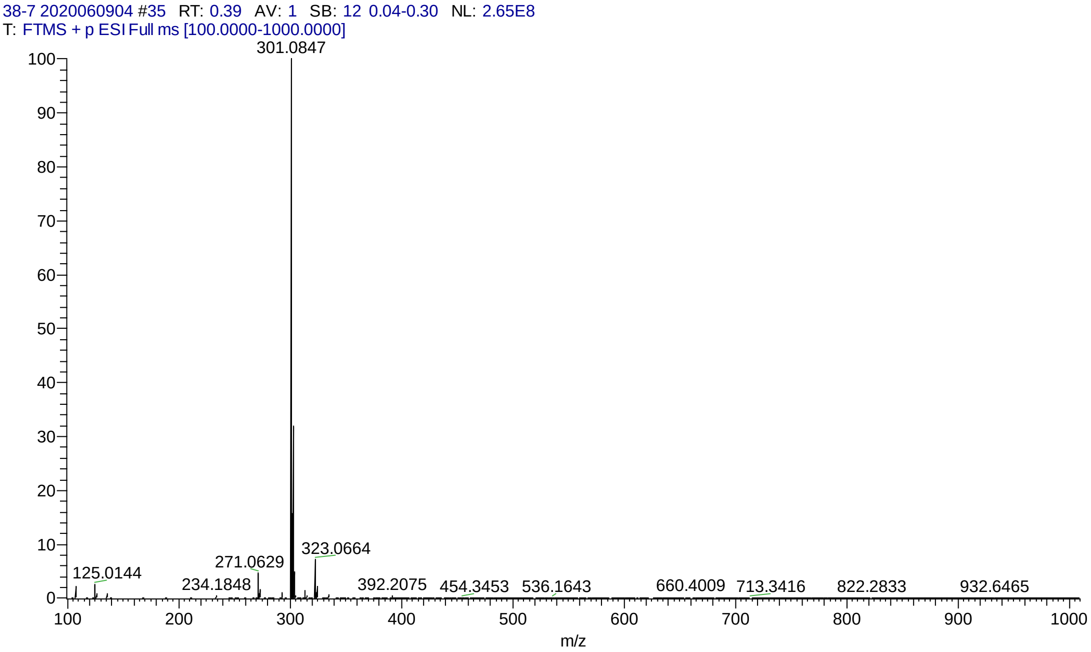

Figure S6. MS spectrum of **10**.

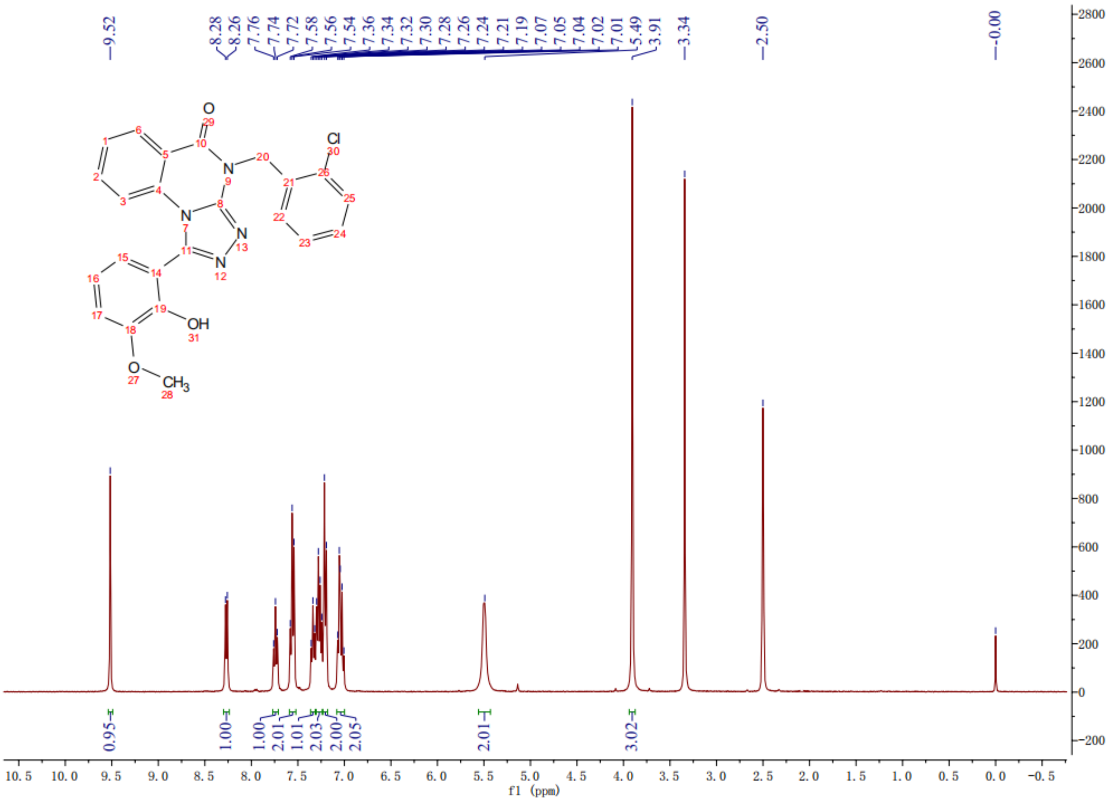

Figure S7. ^{1}H-NMR spectrum of **11.**

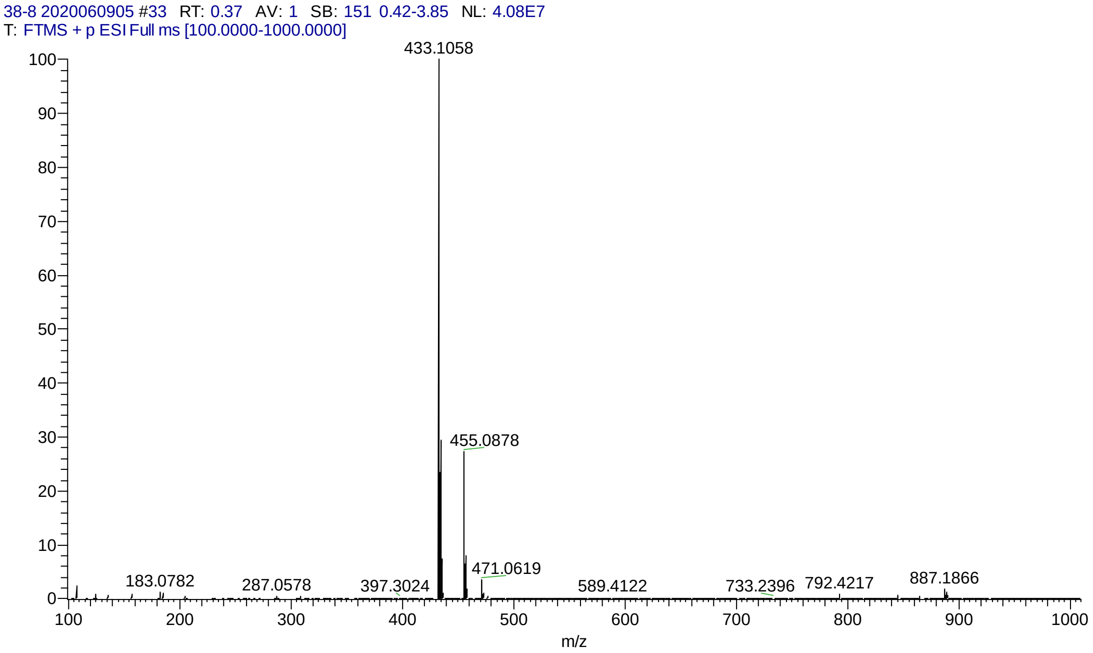

Figure S8. MS spectrum of **11**.

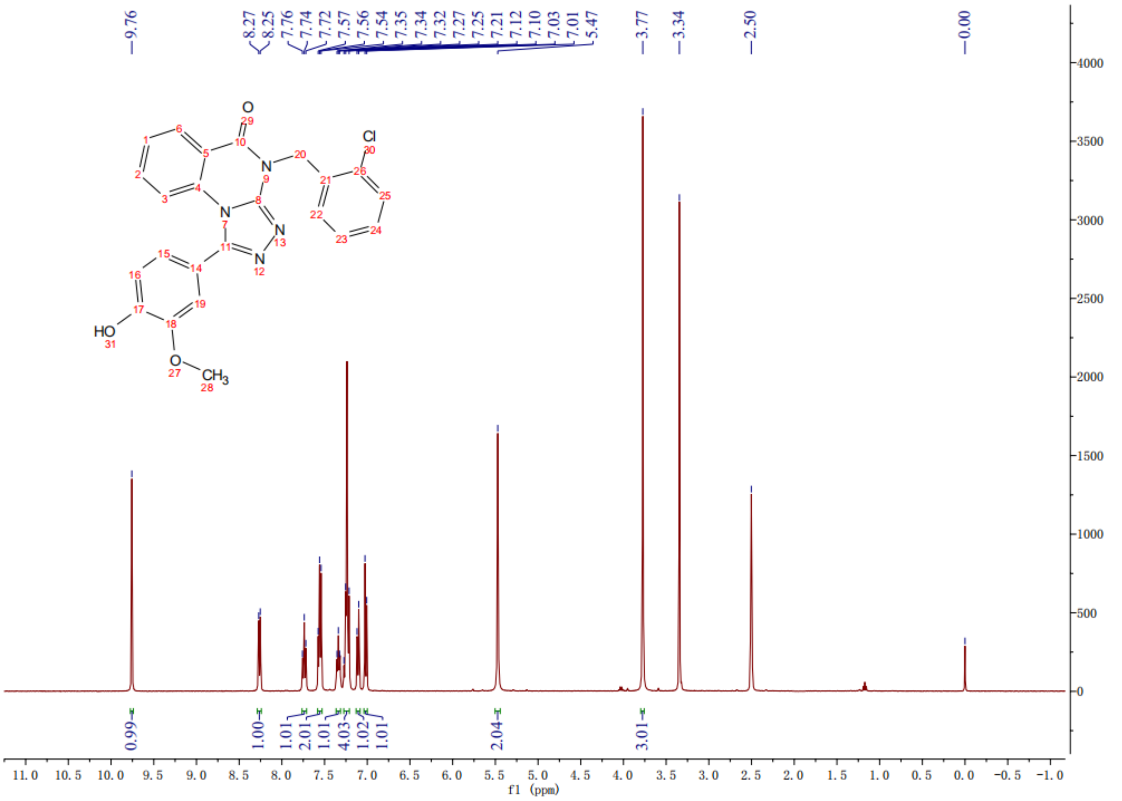

Figure S9. ^{1}H-NMR spectrum of **13.**

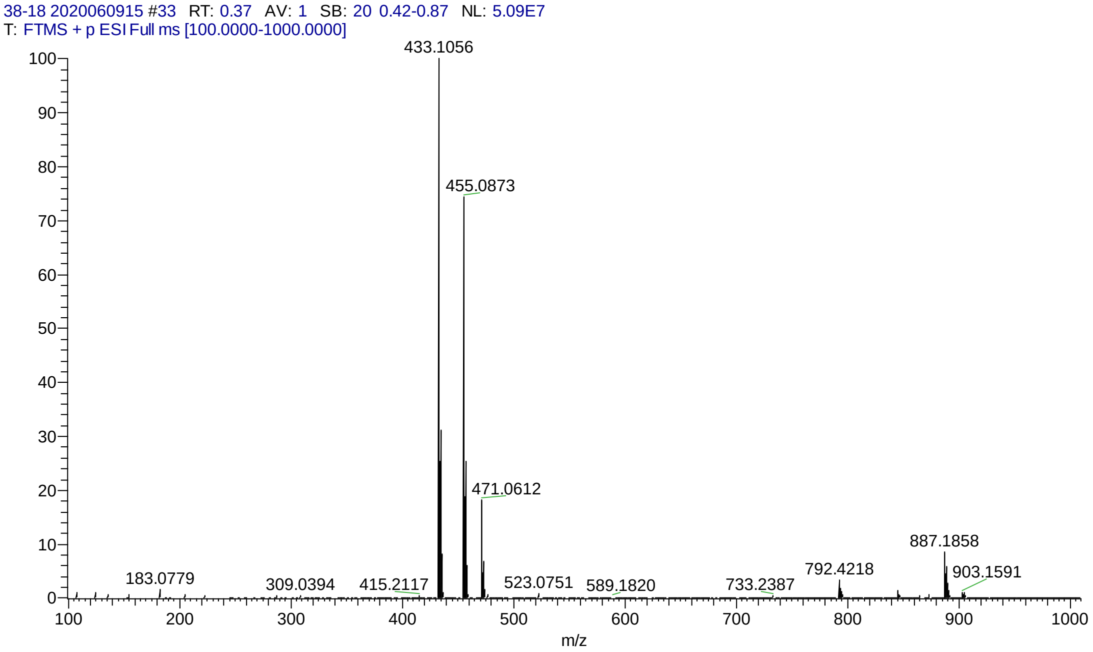

Figure S10. MS spectrum of **13.**

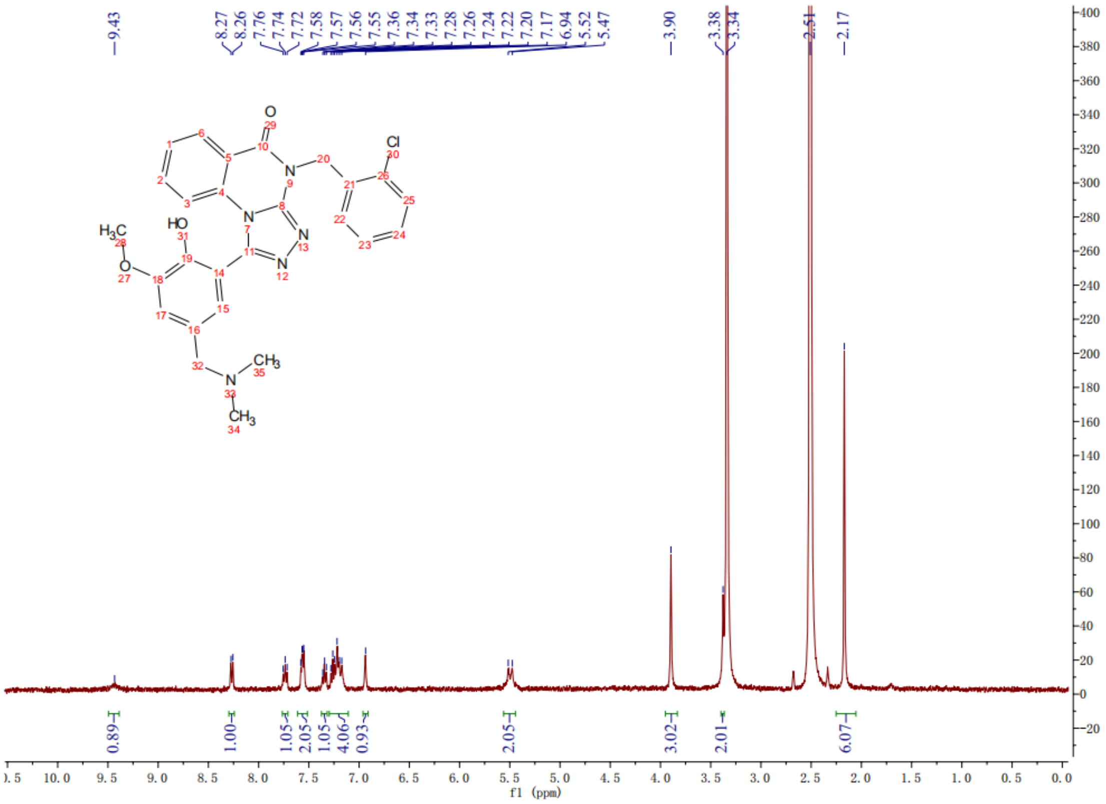

Figure S11. ^{1}H-NMR spectrum of **12a.**

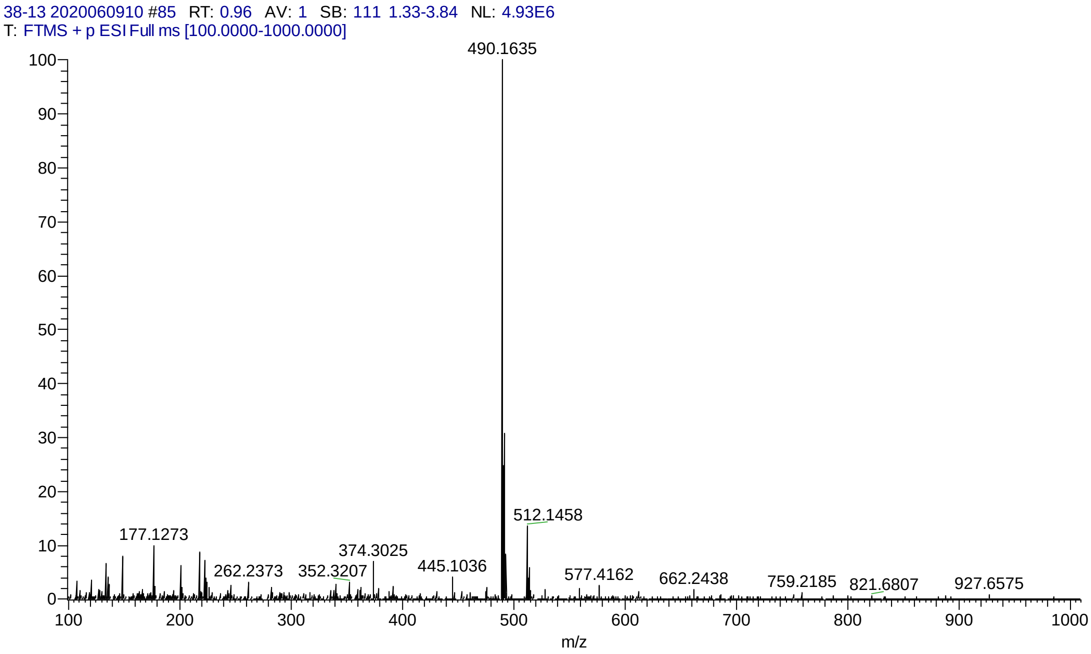

Figure S12. MS spectrum of **12a**.

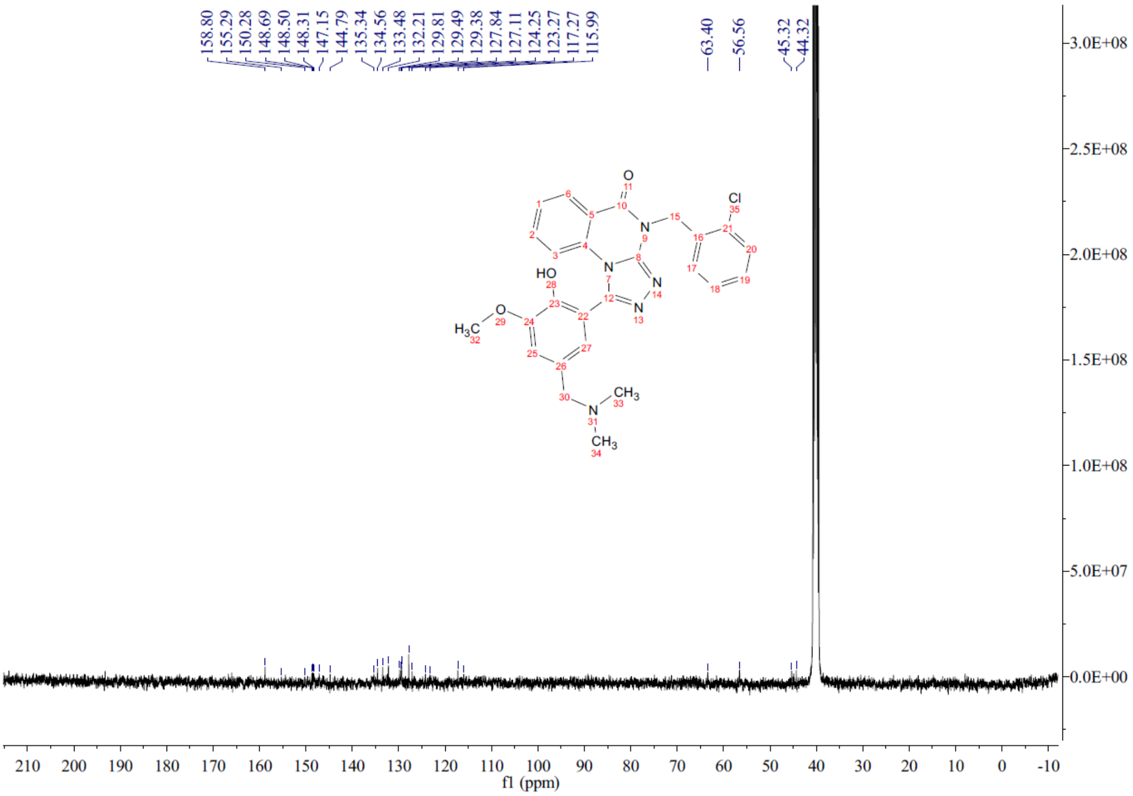

Figure S13.^{13}C-NMR spectrum of **12a**.

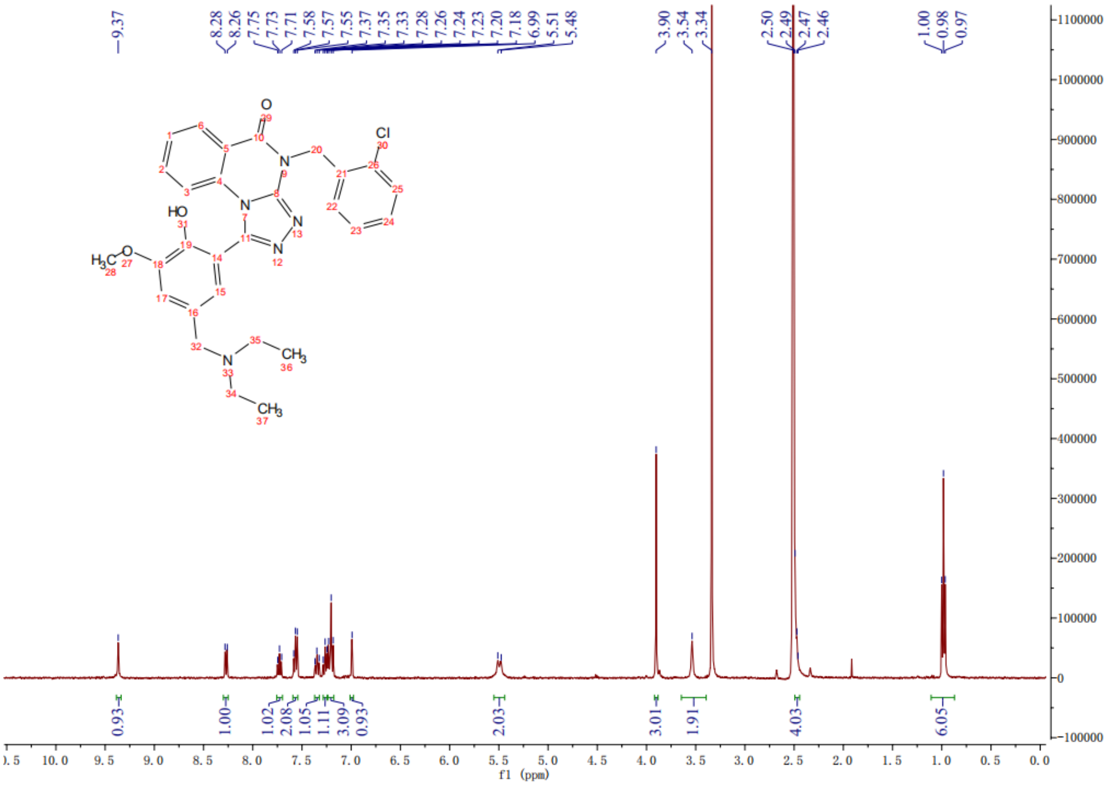

Figure S14. ^{1}H-NMR spectrum of **12b**

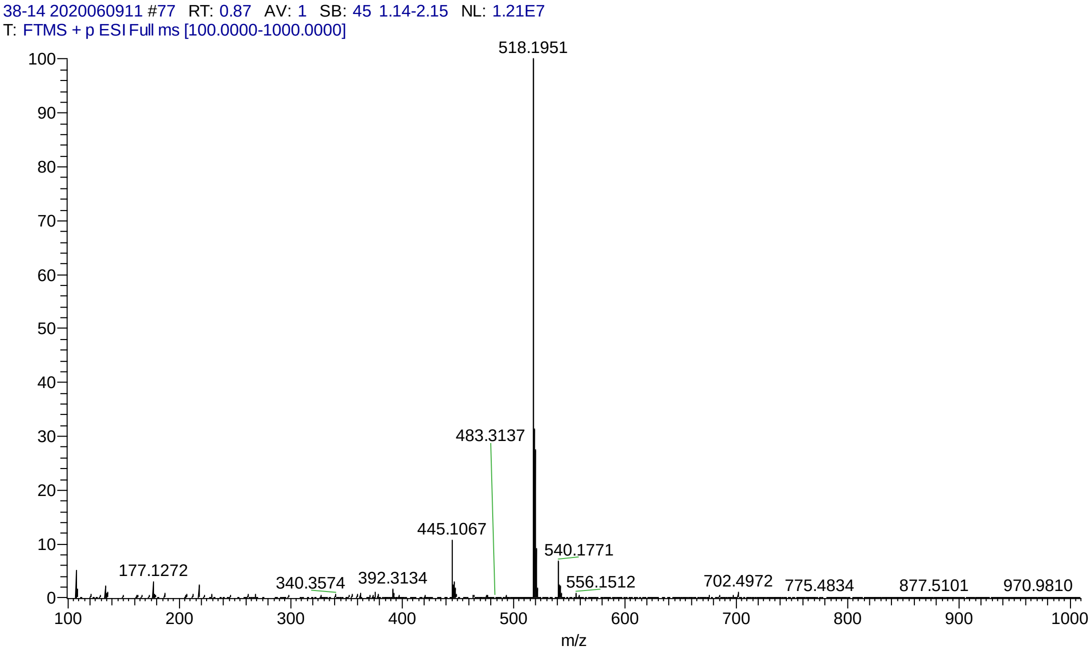

Figure S15. MS spectrum of **12b**.

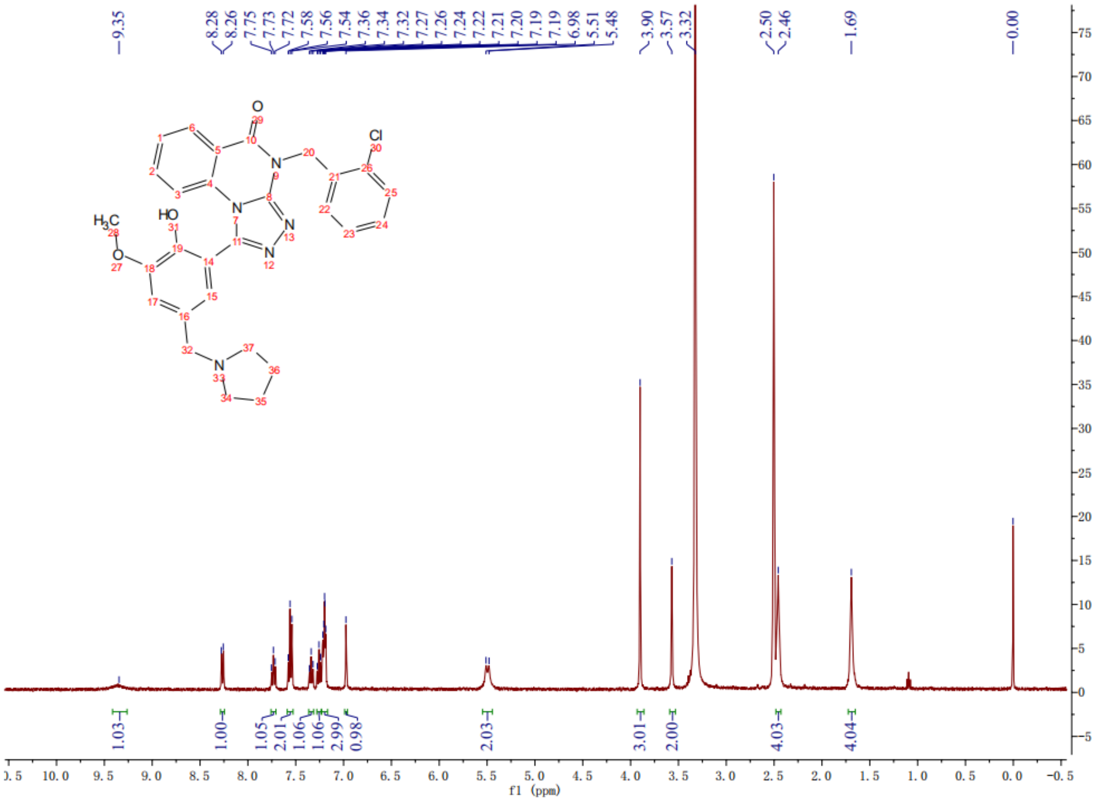

Figure S16. ^{1}H-NMR spectrum of **12c**.

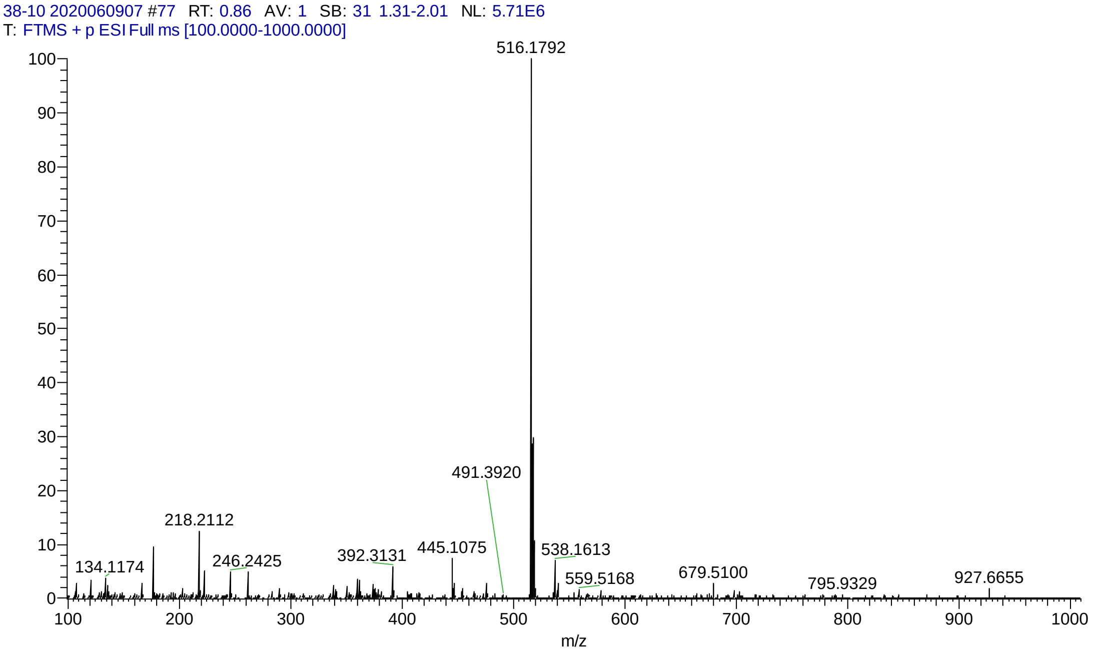

Figure S17. MS spectrum of **12c**.

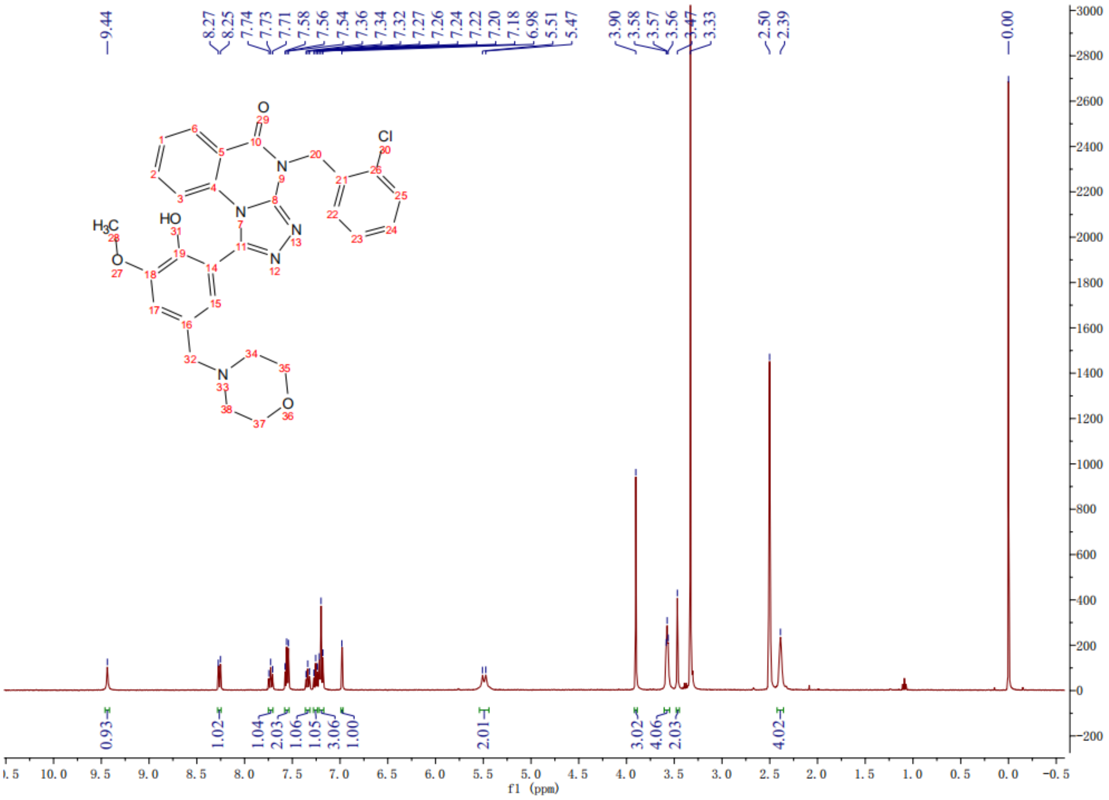

Figure S18. ^{1}H-NMR spectrum of **12d**.

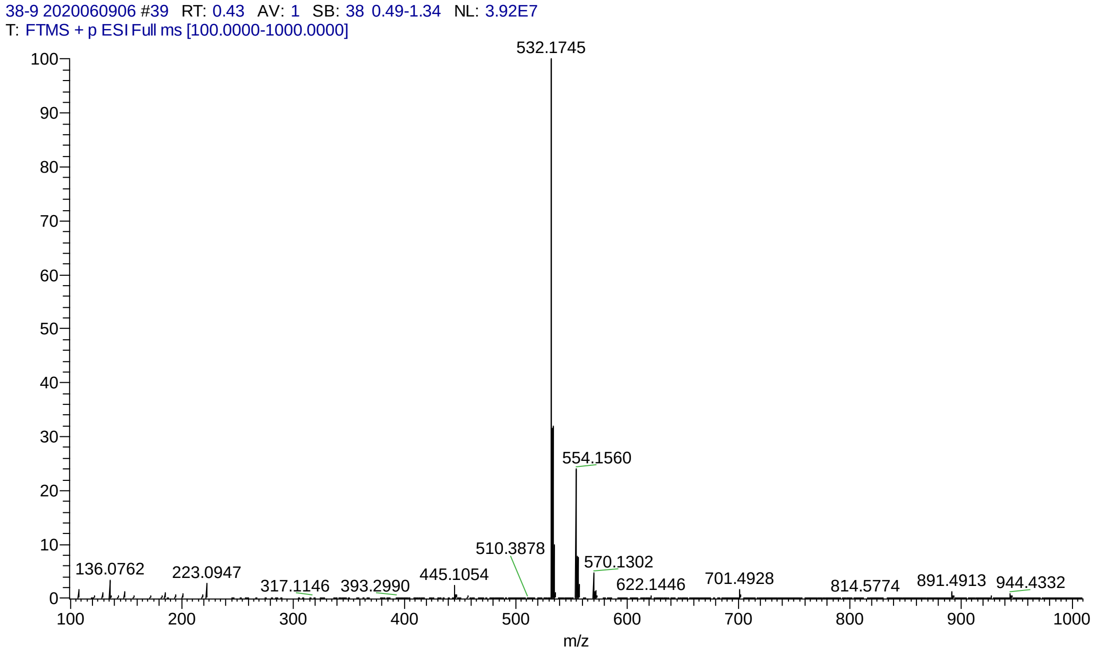

Figure S19. MS spectrum of **12d**.

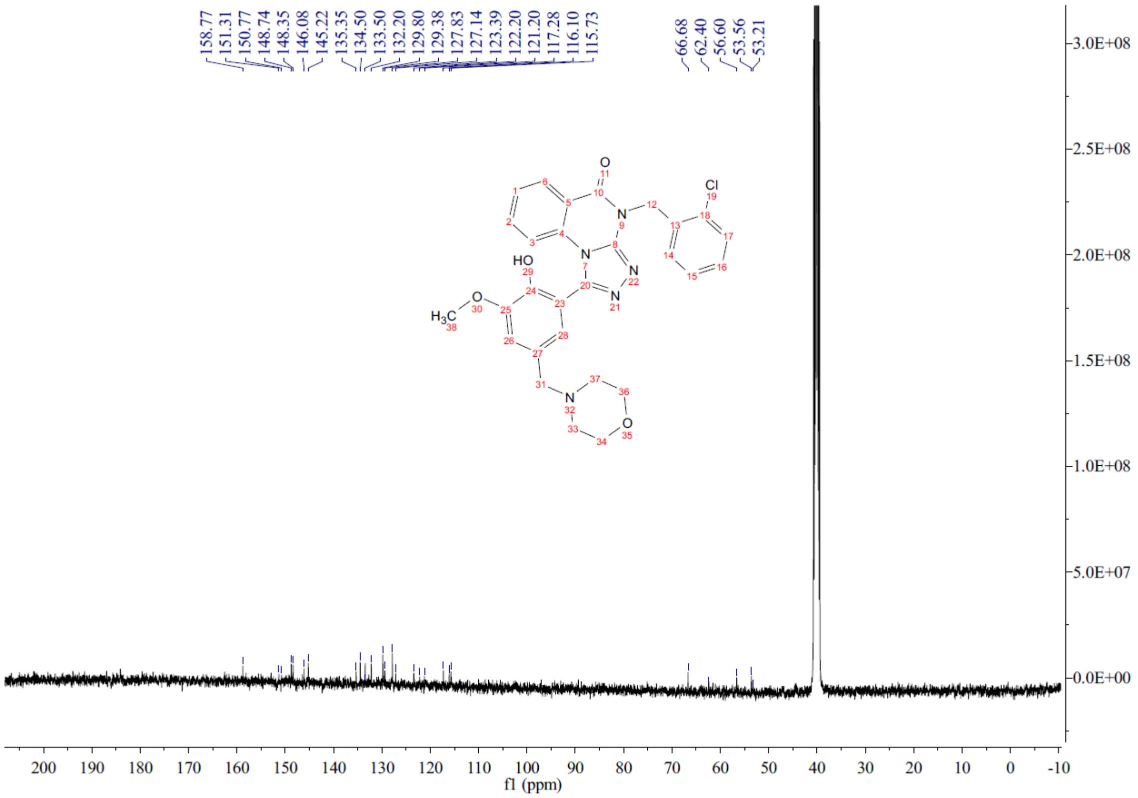

Figure S20.^{13}C-NMR spectrum of **12d**.

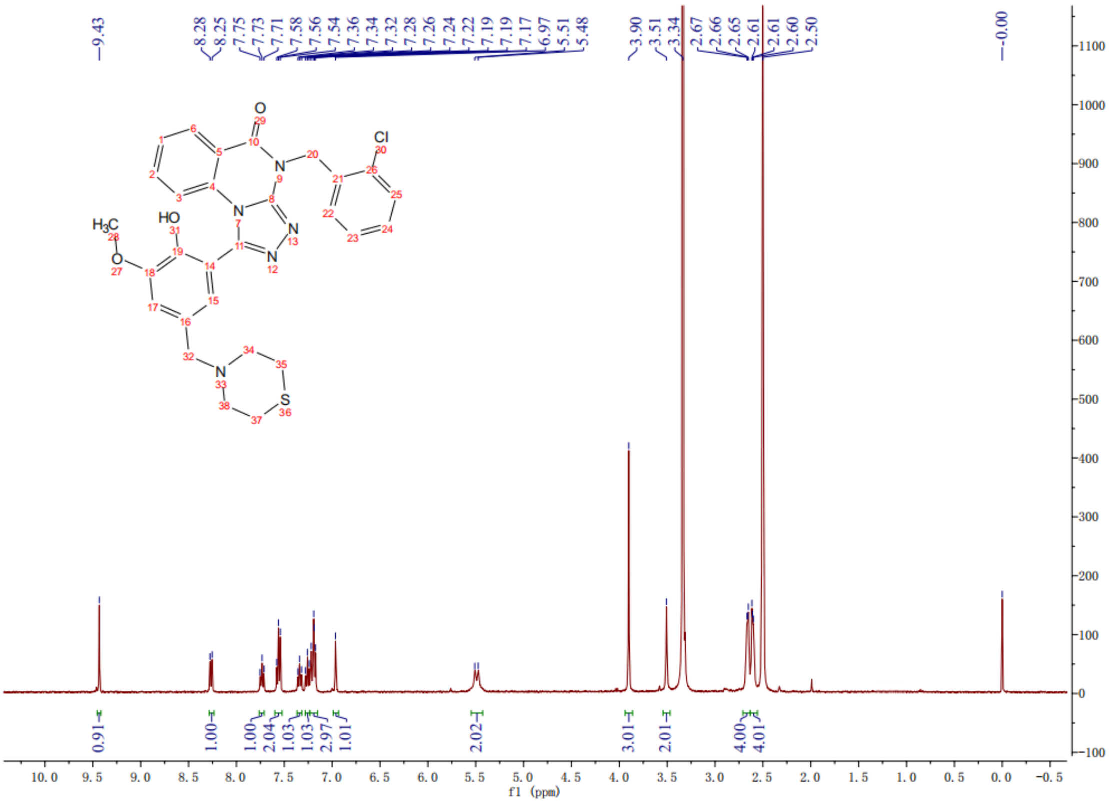

Figure S21. ^{1}H-NMR spectrum of **12e**.

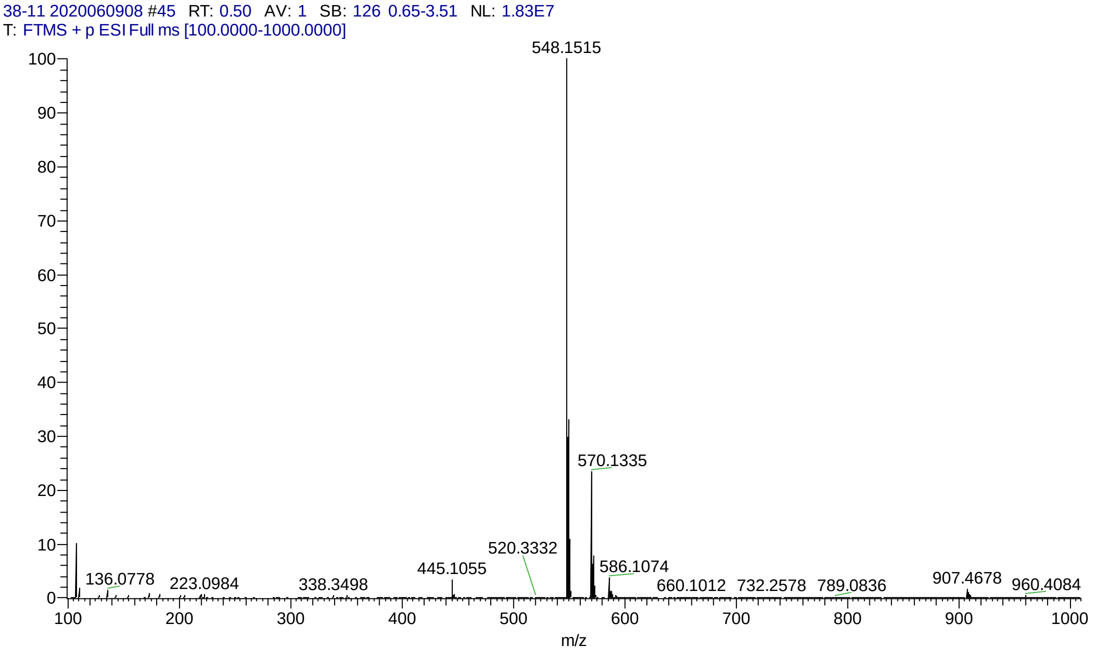

Figure S22. MS spectrum of **12e**.

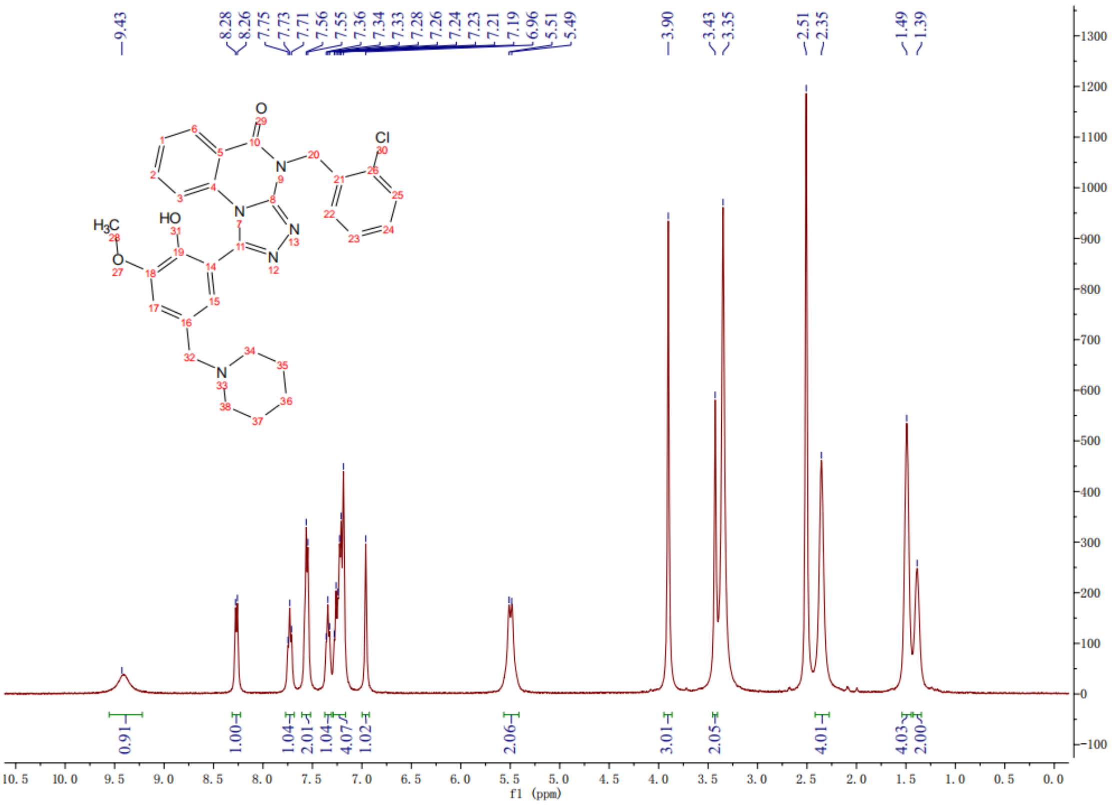

Figure S23. ^{1}H-NMR spectrum of **12f**.

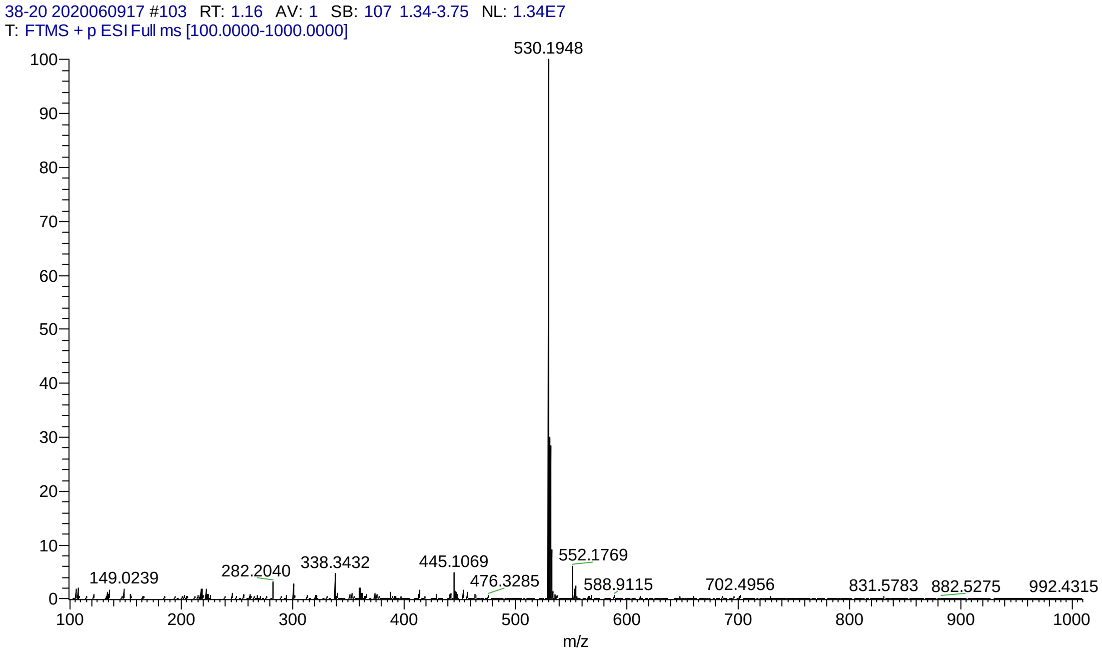

Figure S24. MS spectrum of **12f**.

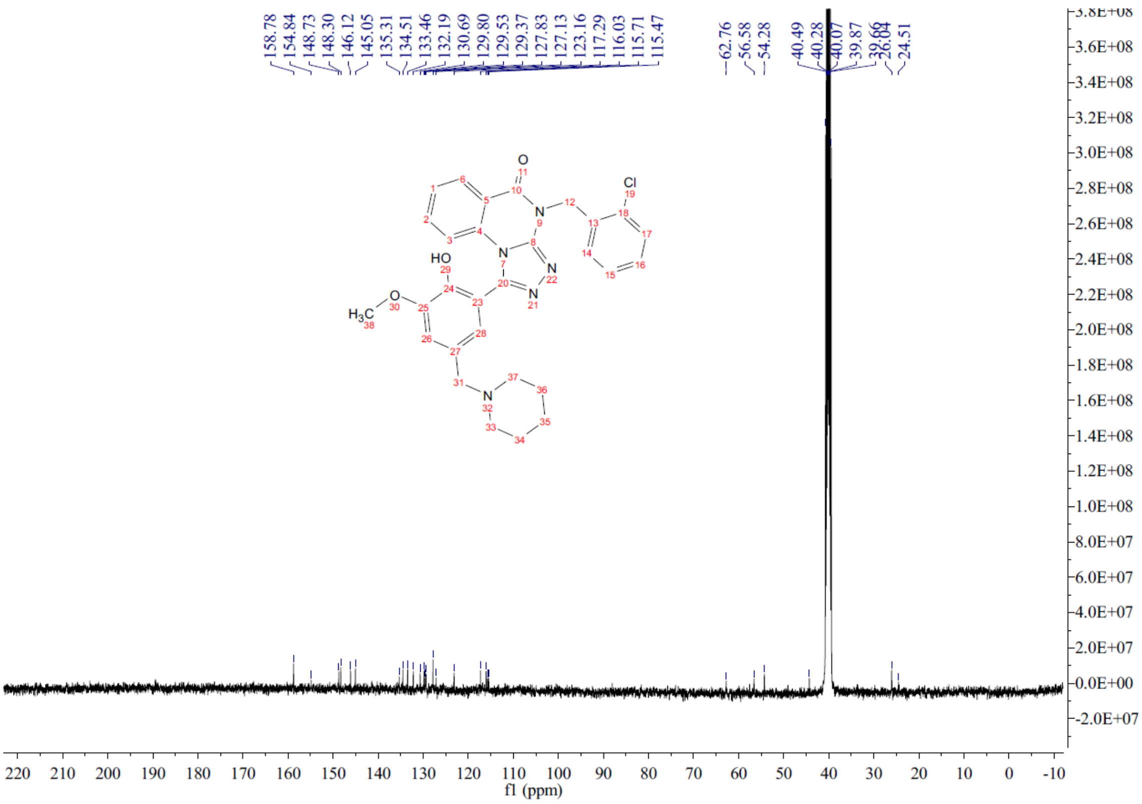

Figure S125.^{13}C-NMR spectrum of **12f**.

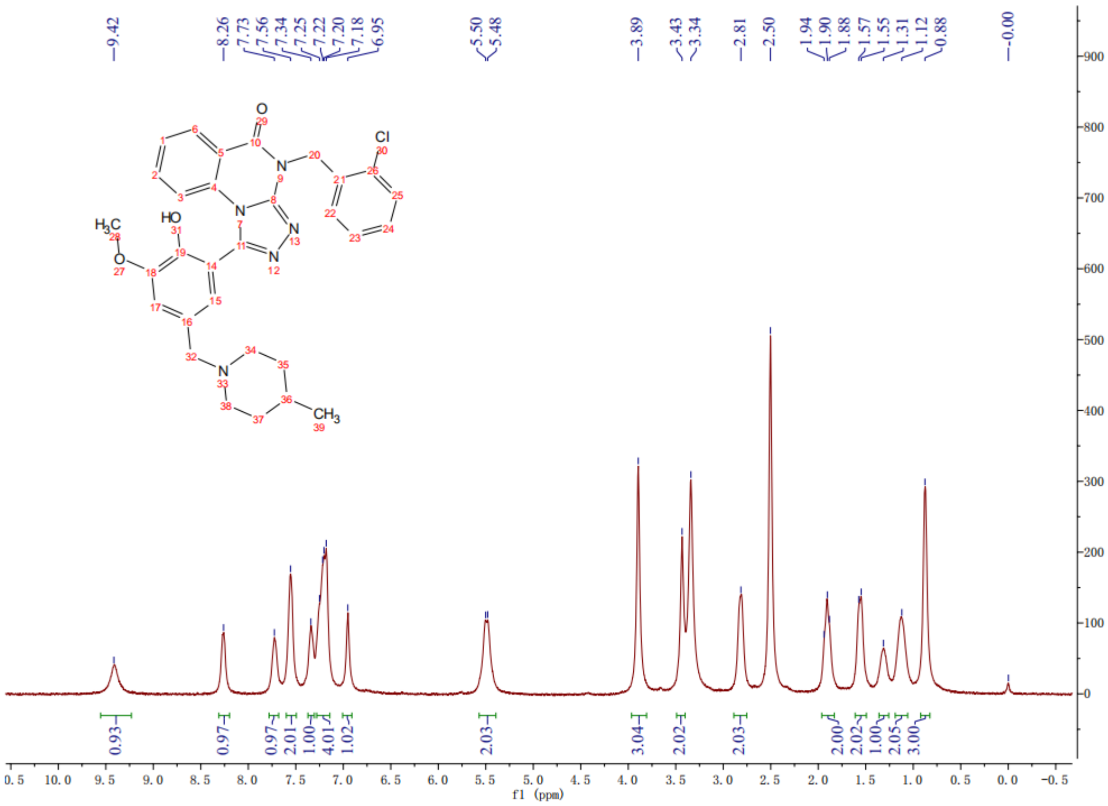

Figure S26. ^{1}H-NMR spectrum of **12g**.

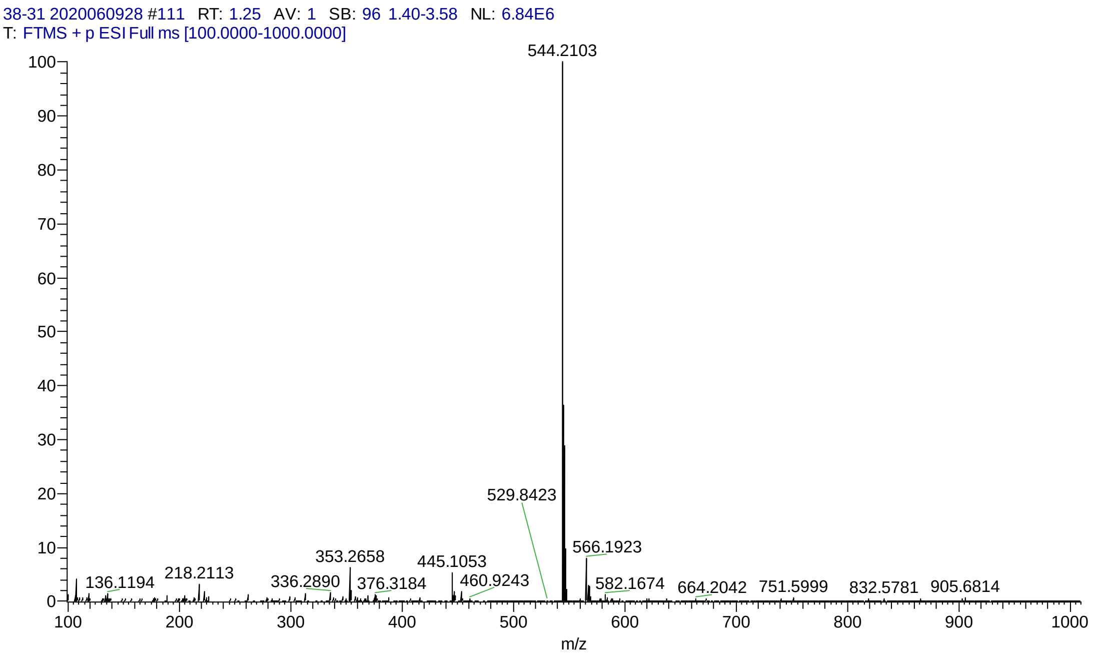

Figure S27. MS spectrum of **12g**.

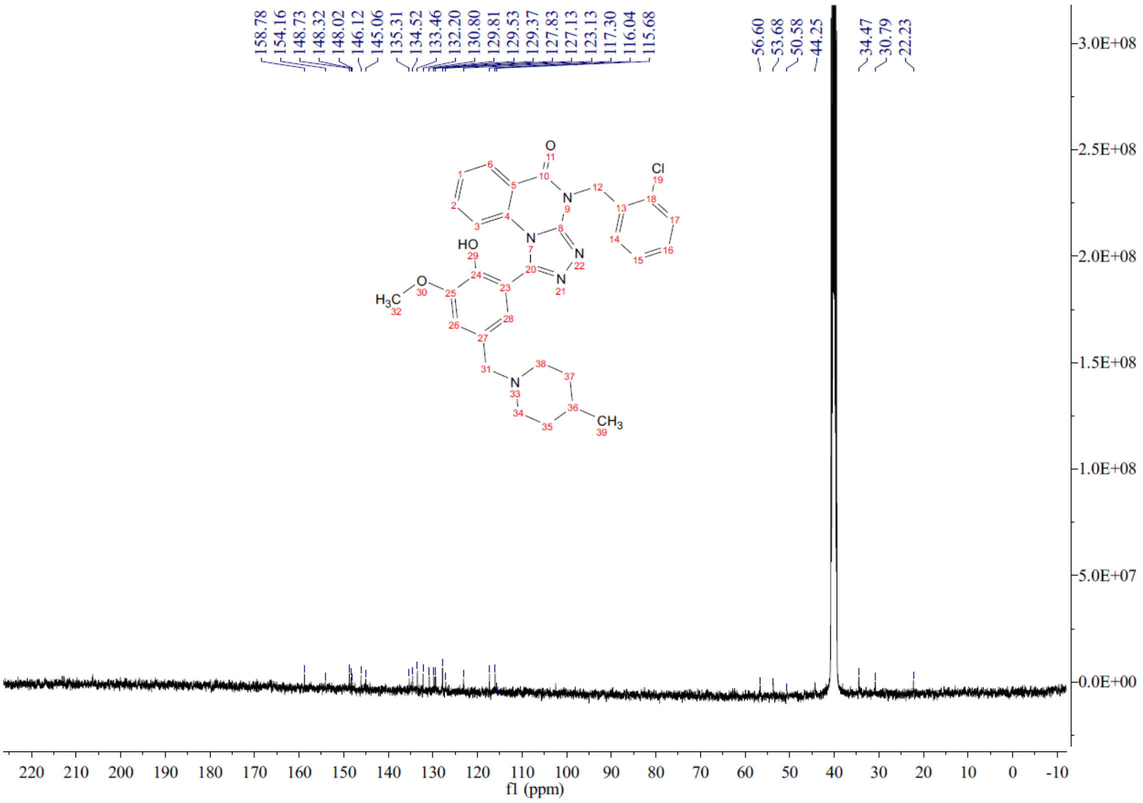

Figure S28.^{13}C-NMR spectrum of **12g**.

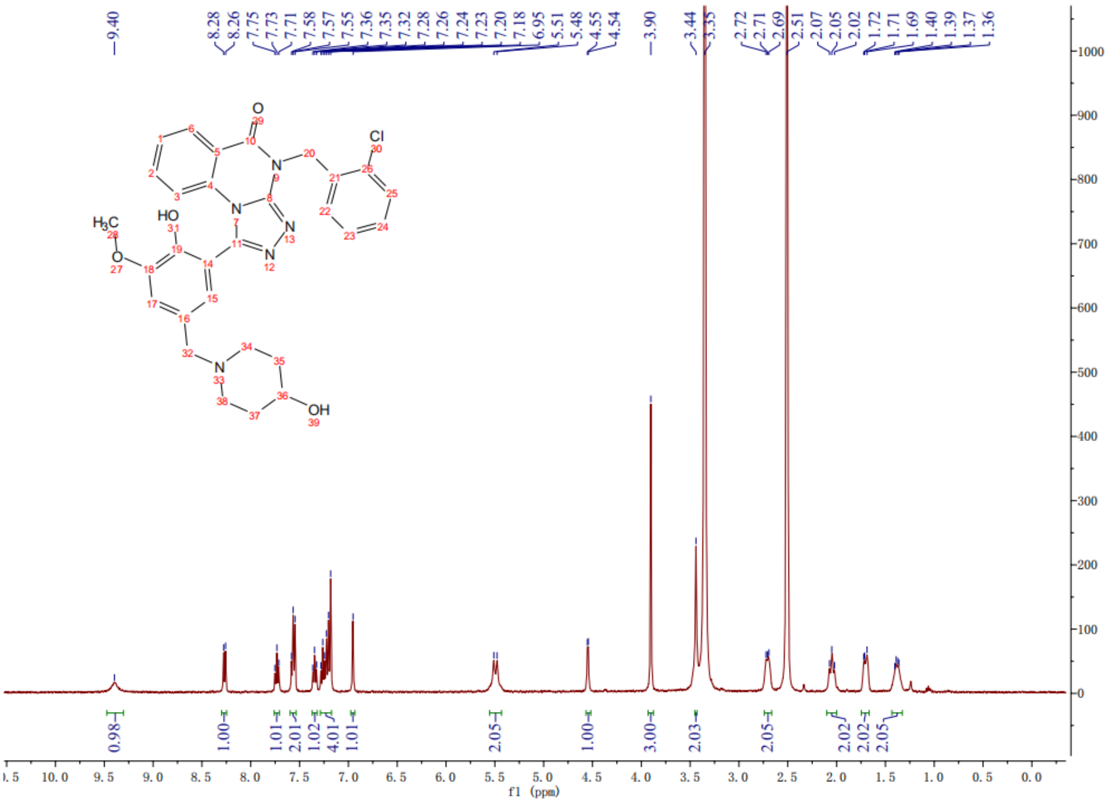

Figure S29. ^{1}H-NMR spectrum of **12h**.

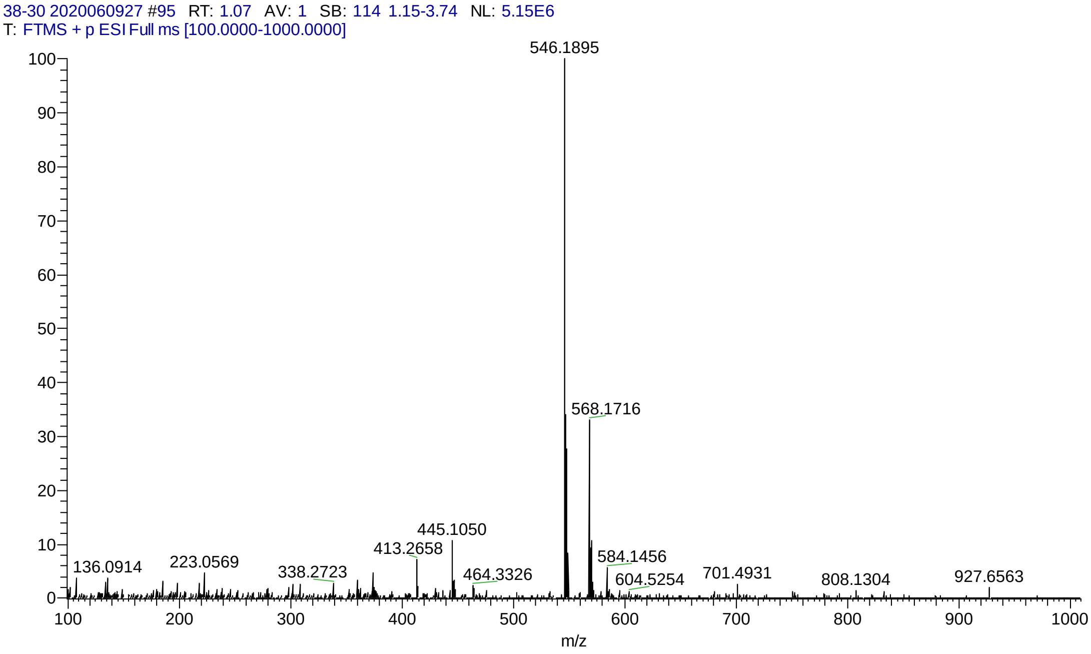

Figure S30. MS spectrum of **12h**.

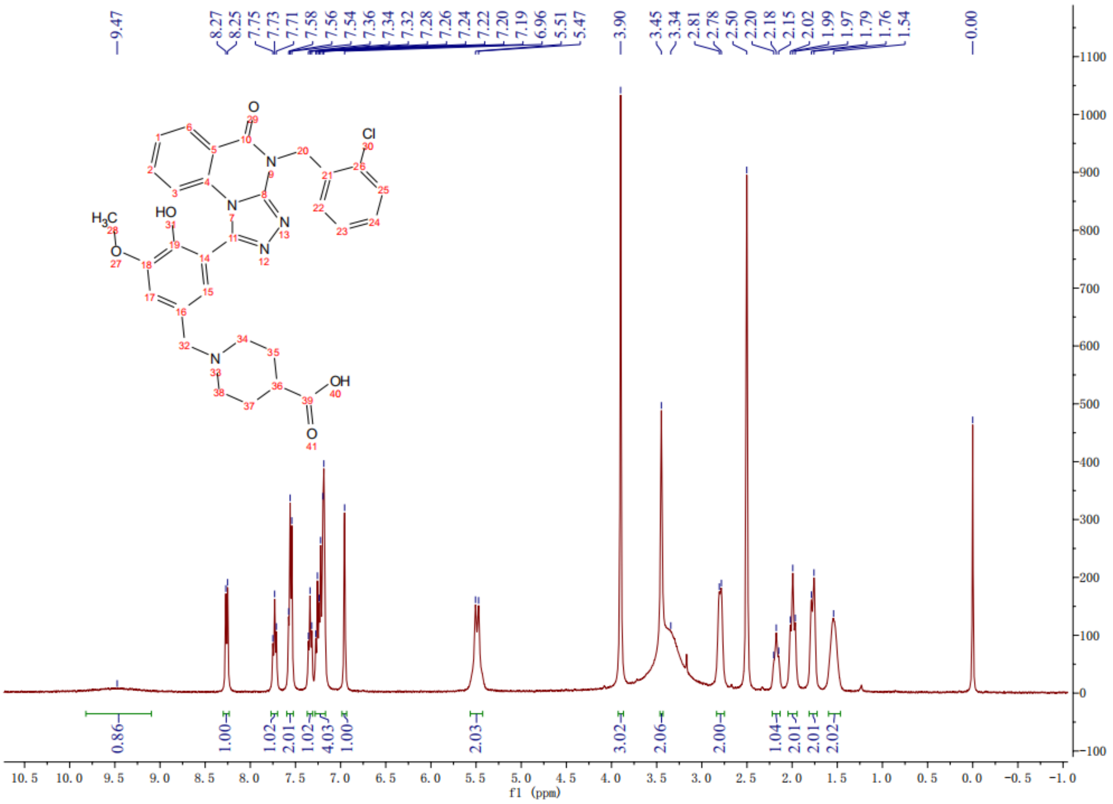

Figure S31. ^{1}H-NMR spectrum of **12i.**

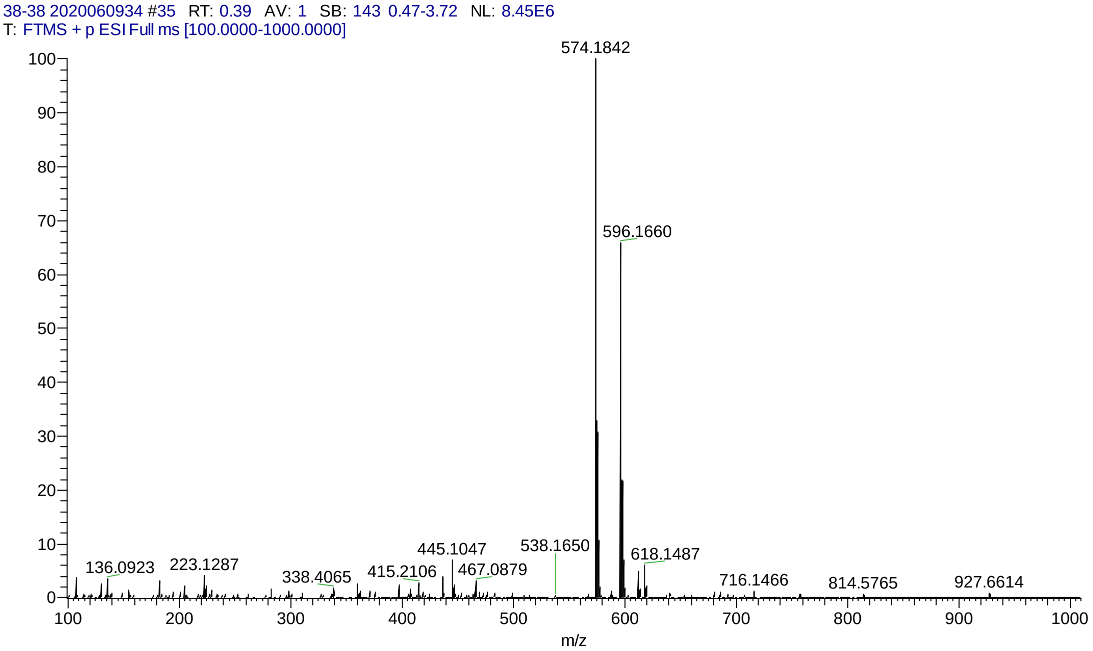

Figure S32. MS spectrum of **12i.**

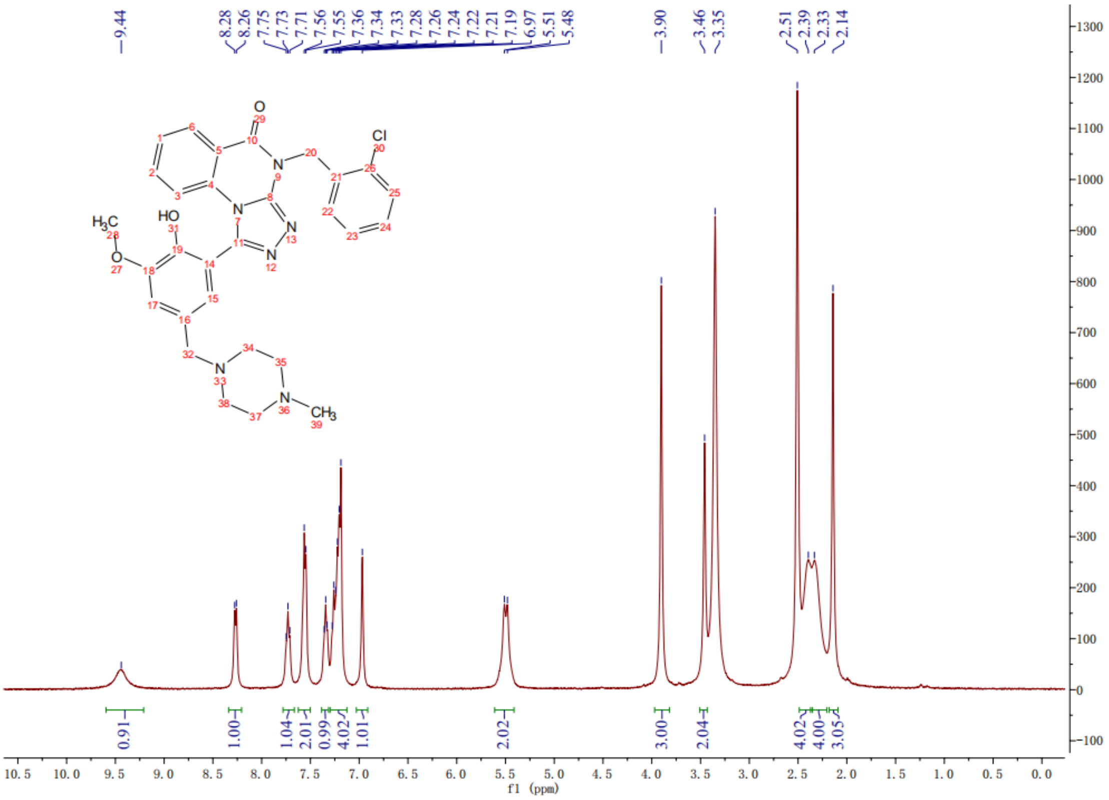

Figure S33. ^{1}H-NMR spectrum of **12j**.

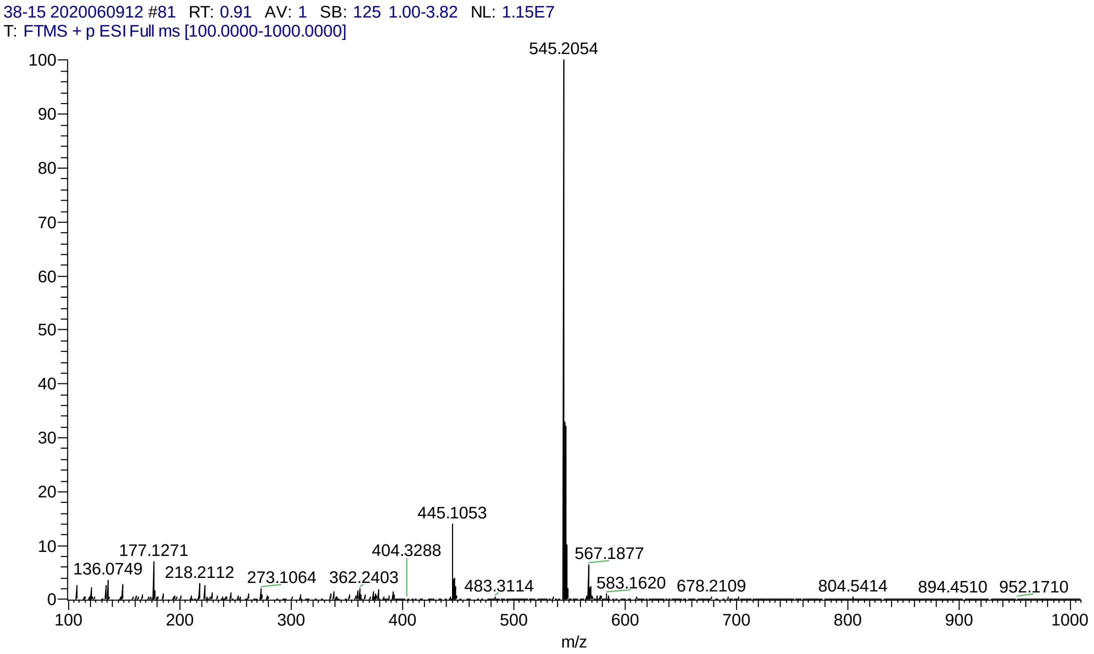

Figure S34. MS spectrum of **12j.**

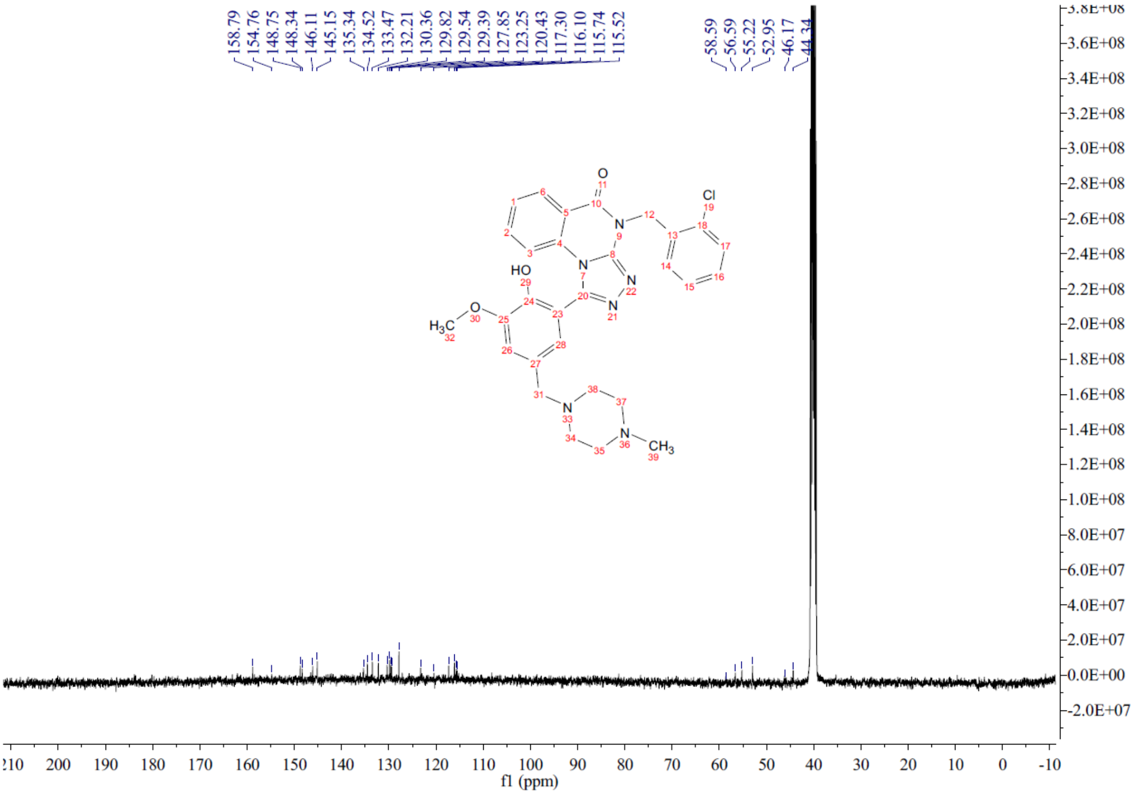

Figure S35.^{13}C-NMR spectrum of **12j**.

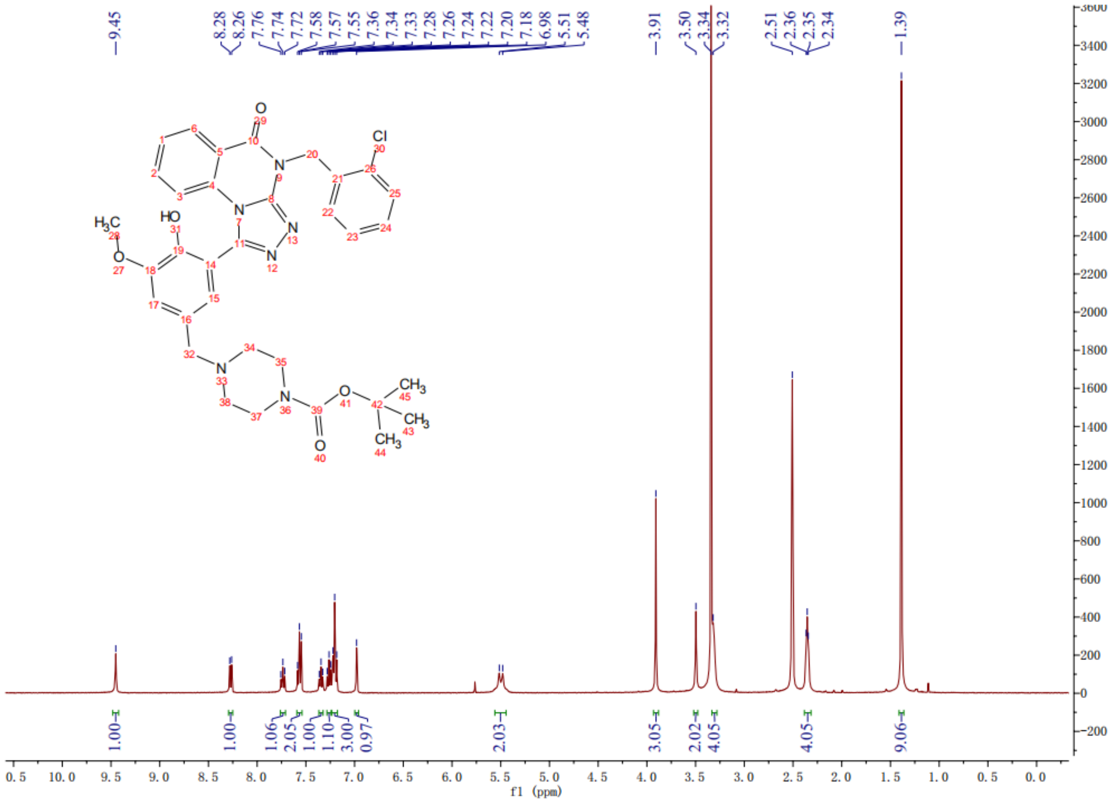

Figure S36. ^{1}H-NMR spectrum of **12k**

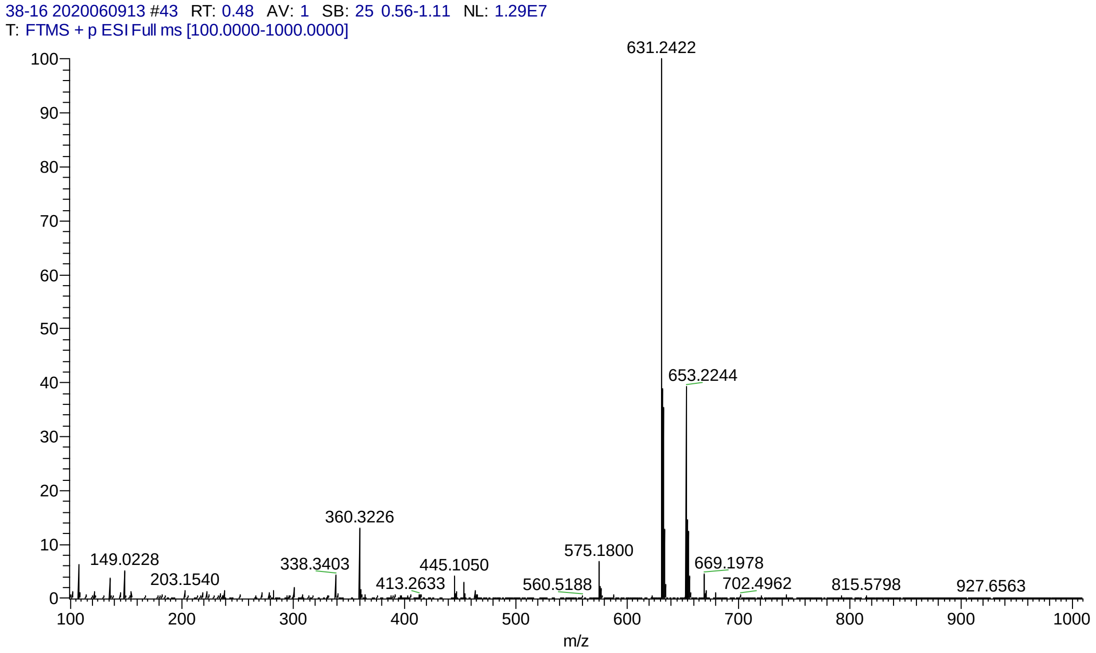

Figure S37. MS spectrum of **12k.**

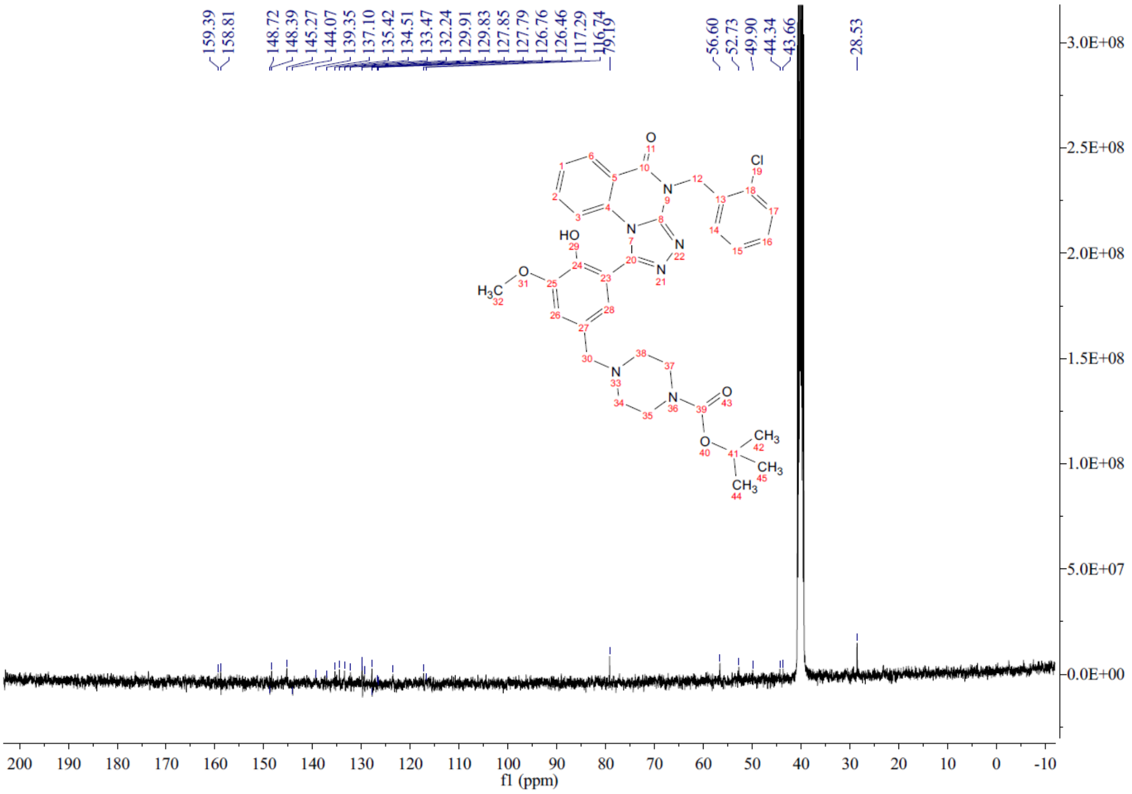

Figure S38.^{13}C-NMR spectrum of **12k**.

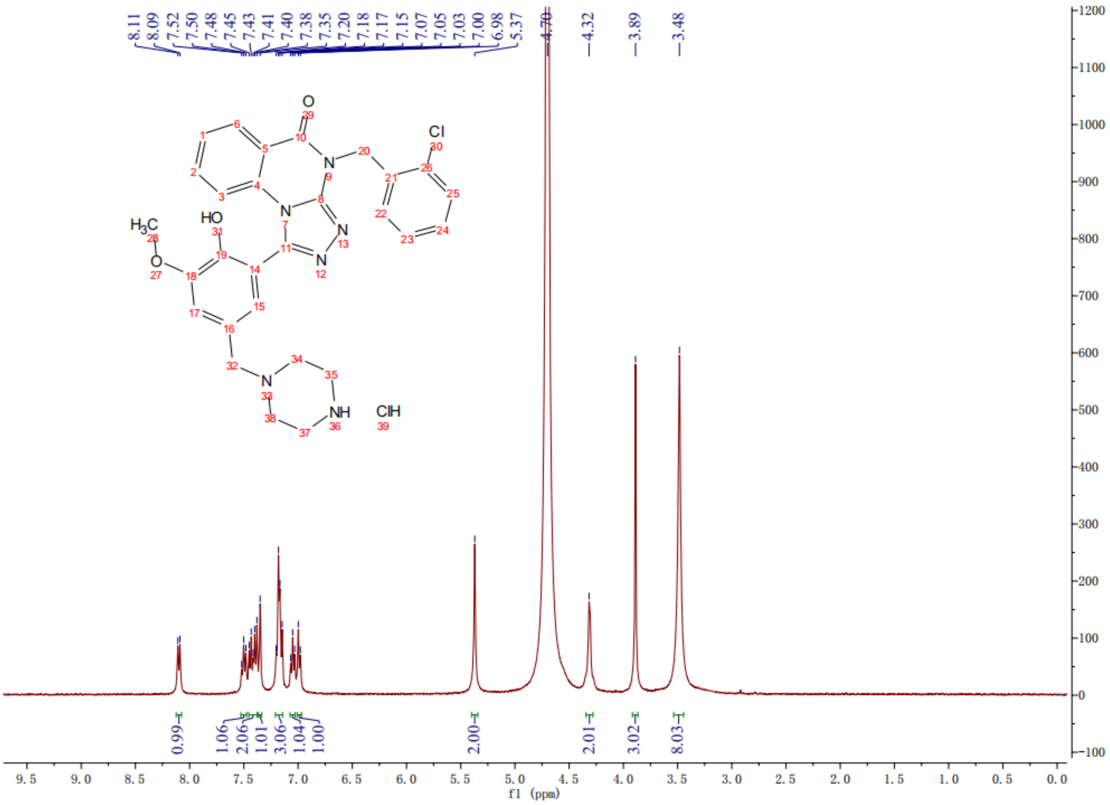

Figure S39. ^{1}H-NMR spectrum of **12l**

38-17 2020060914 #125 RT: 1.41 AV: 1 SB: 108 1.52-3.97 NL: 8.34E5
T: FTMS + p ESI Full ms [100.0000-1000.0000]

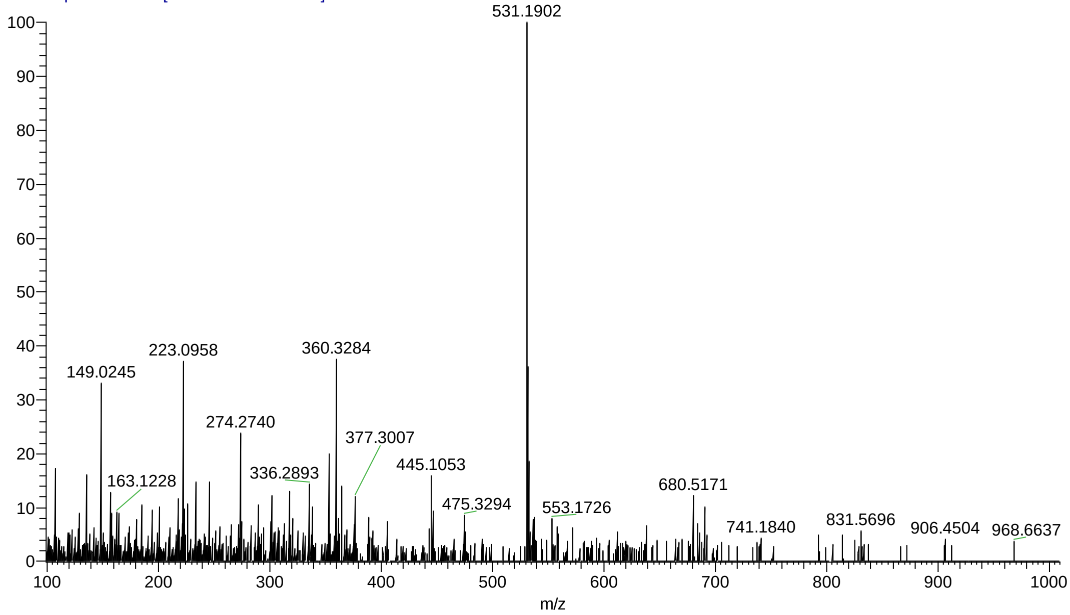

Figure S40. MS spectrum of **12l.**

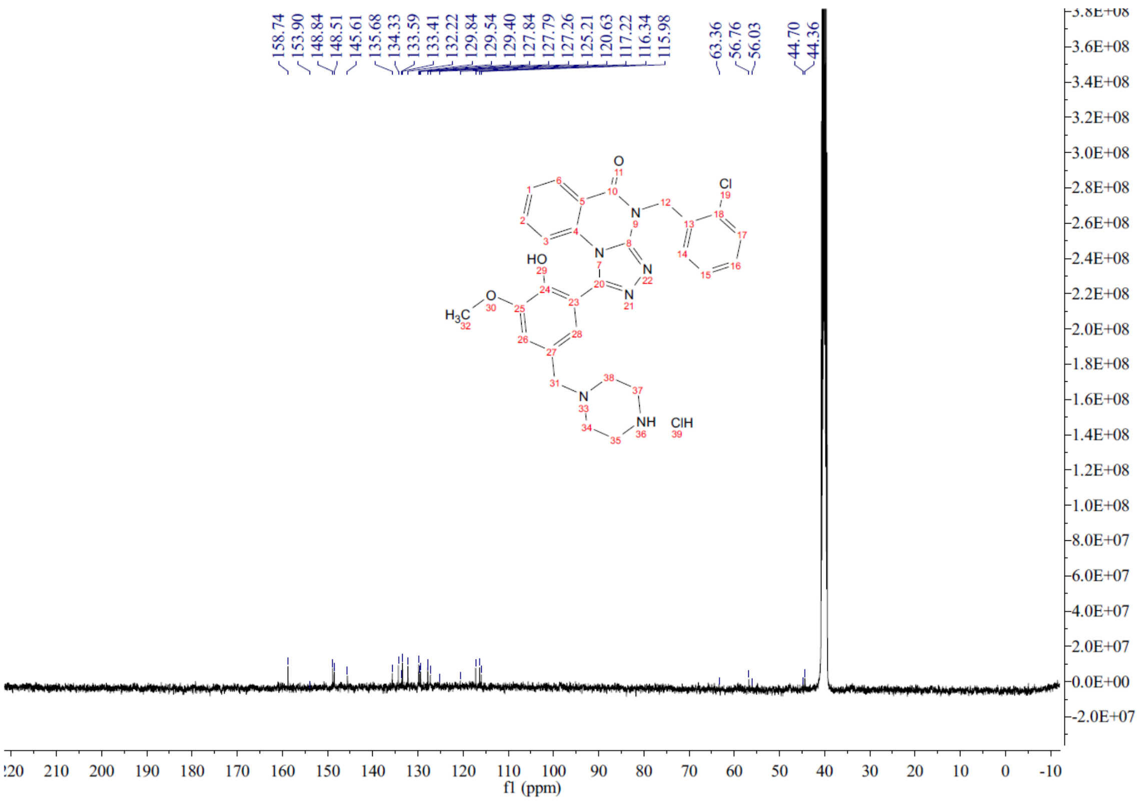

Figure S41.^{13}C-NMR spectrum of **12l**.

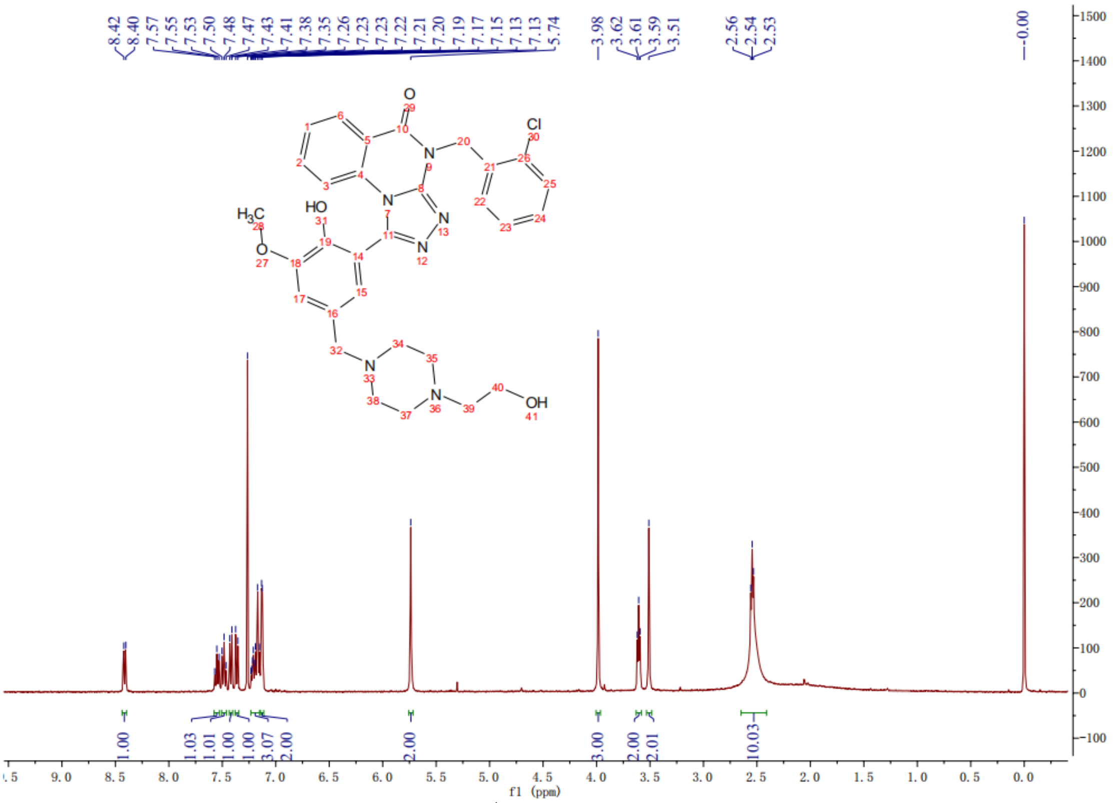

Figure S42. ^{1}H-NMR spectrum of **12m.**

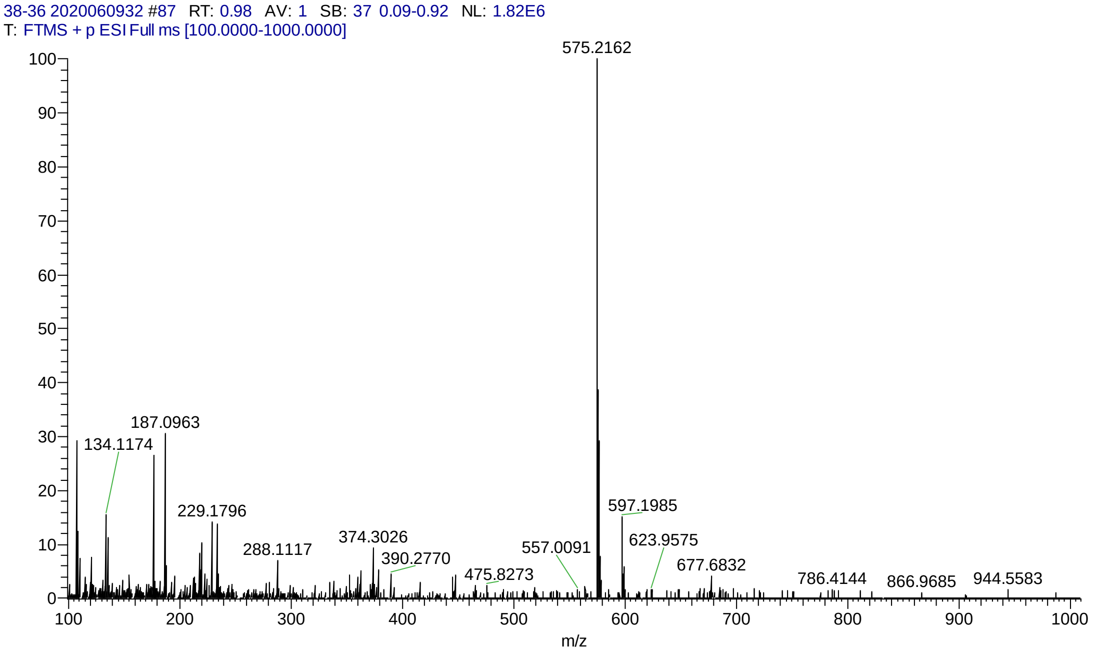

Figure S43. MS spectrum of **12m.**

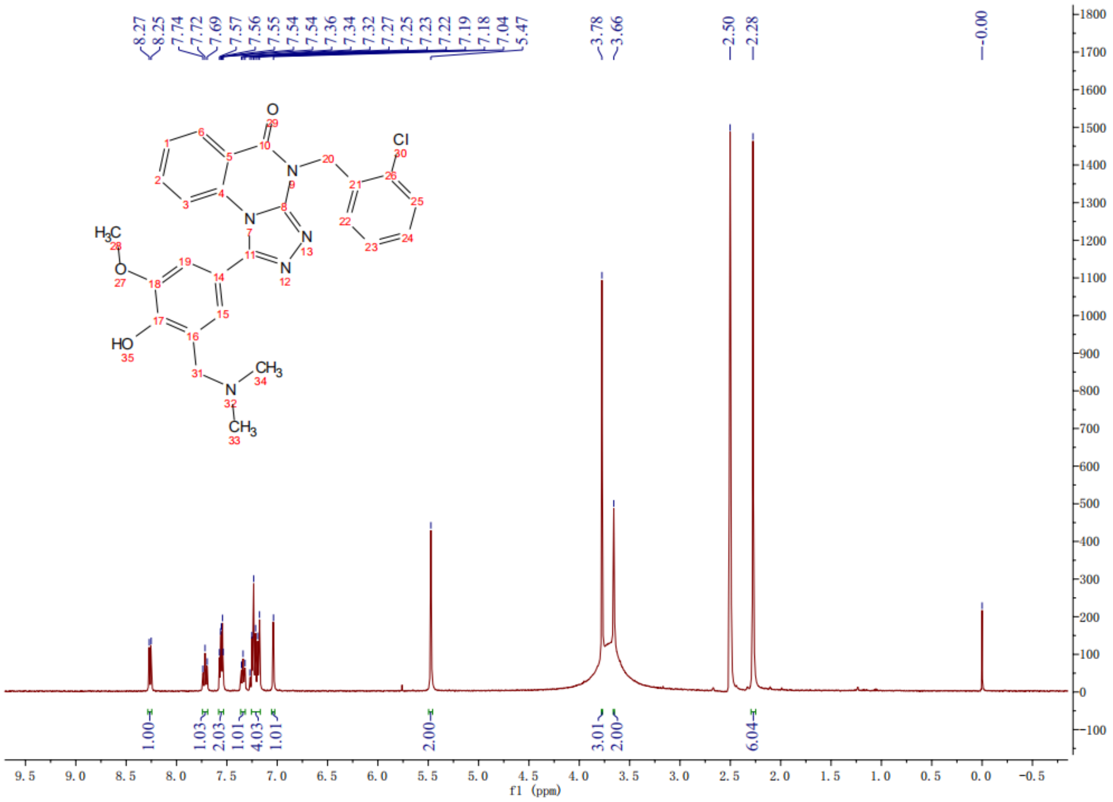

Figure S44. ^{1}H-NMR spectrum of **14a.**

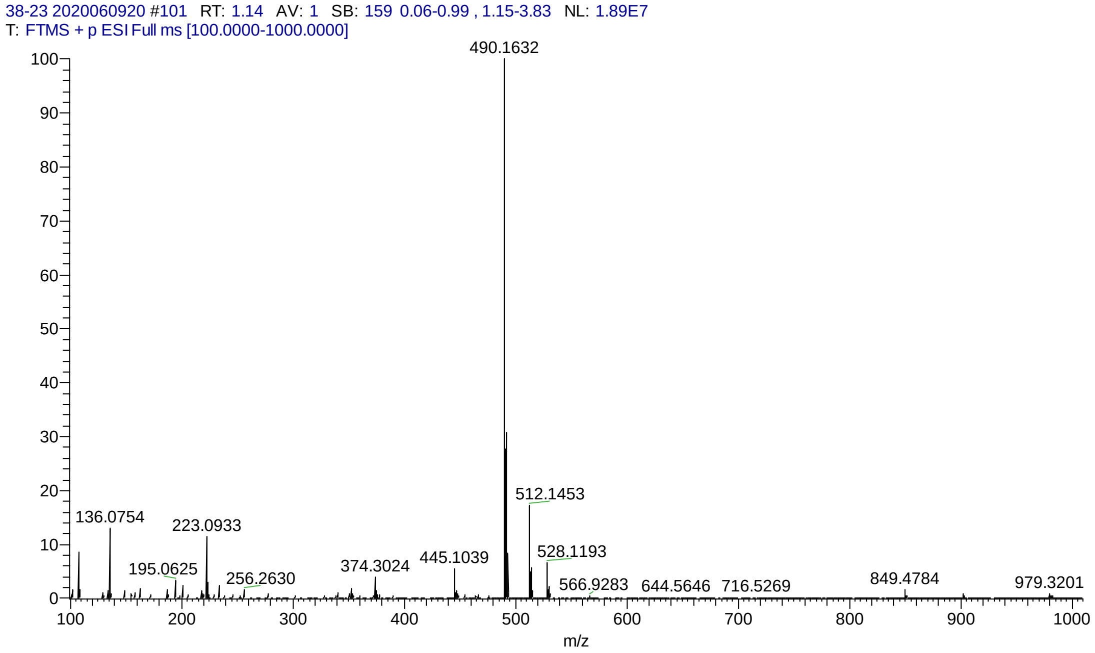

Figure S45. MS spectrum of **14a.**

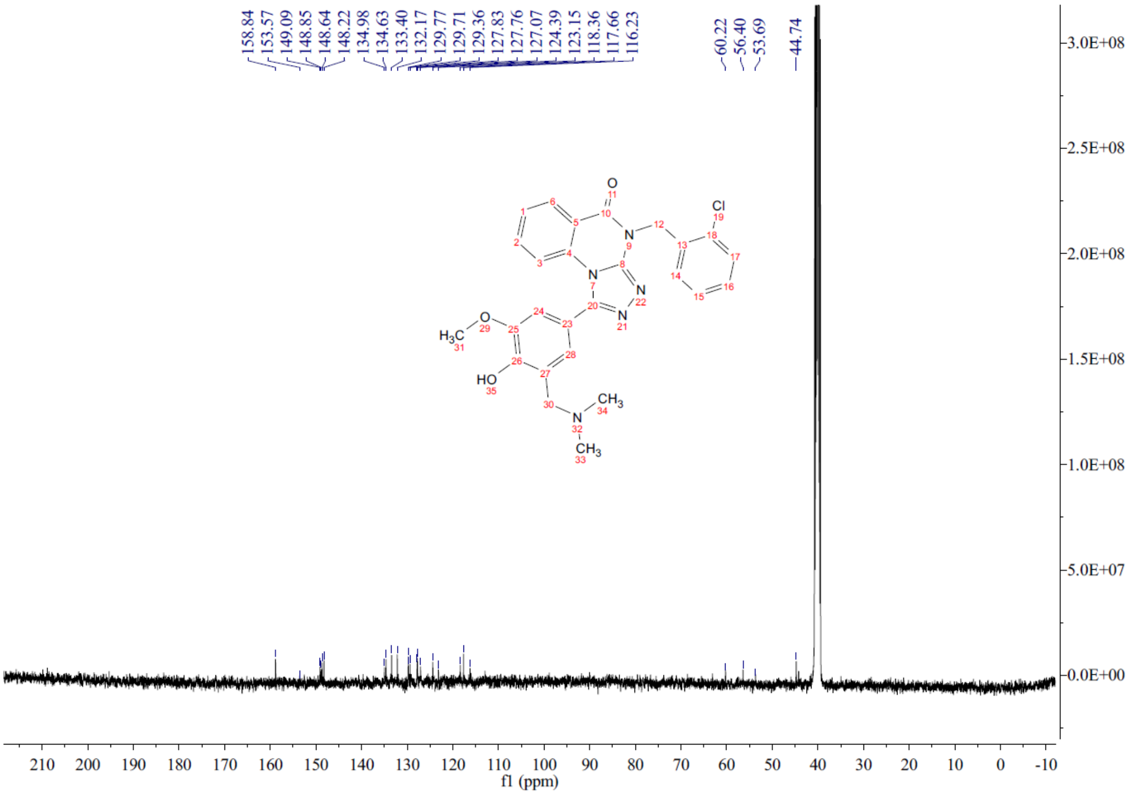

Figure S46.^{13}C-NMR spectrum of **14a**.

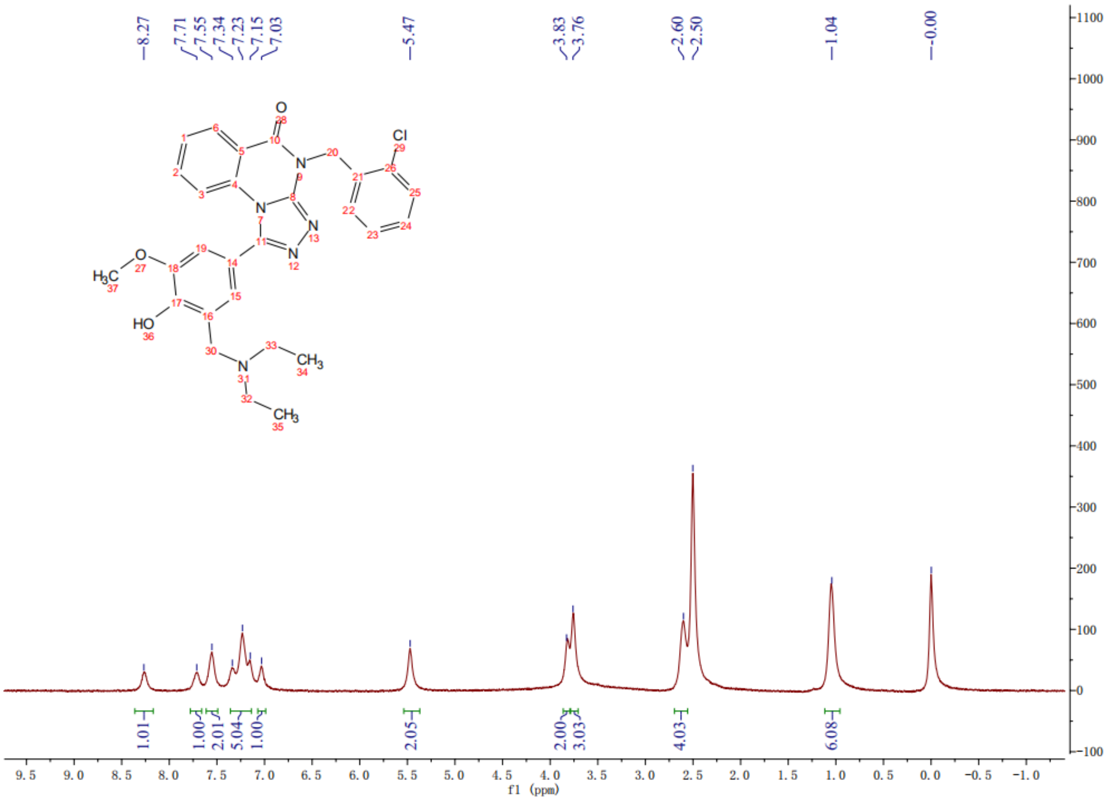

Figure S47. ^{1}H-NMR spectrum of **14b.**

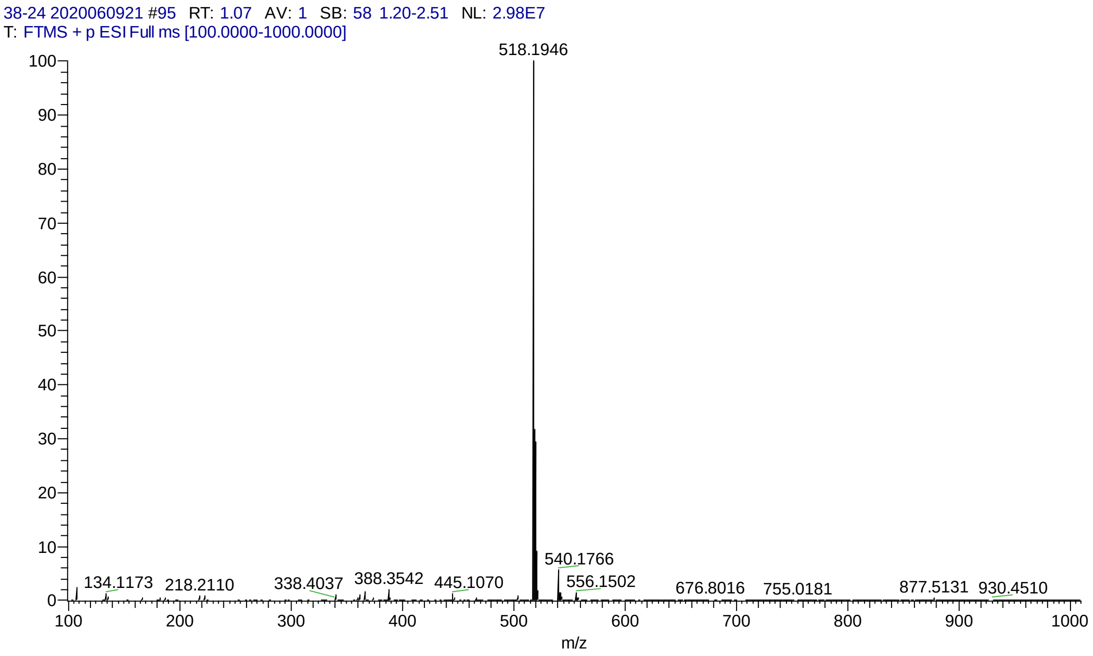

Figure S48. MS spectrum of **14b.**

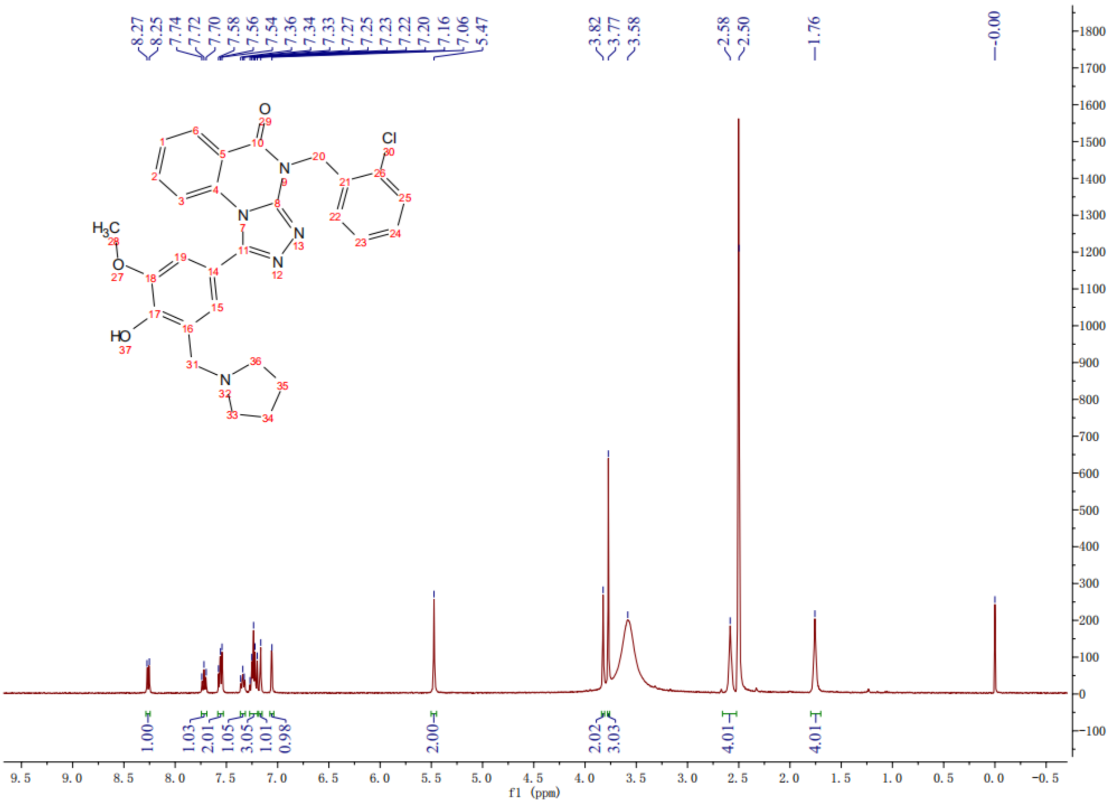

Figure S49. ^{1}H-NMR spectrum of **14c.**

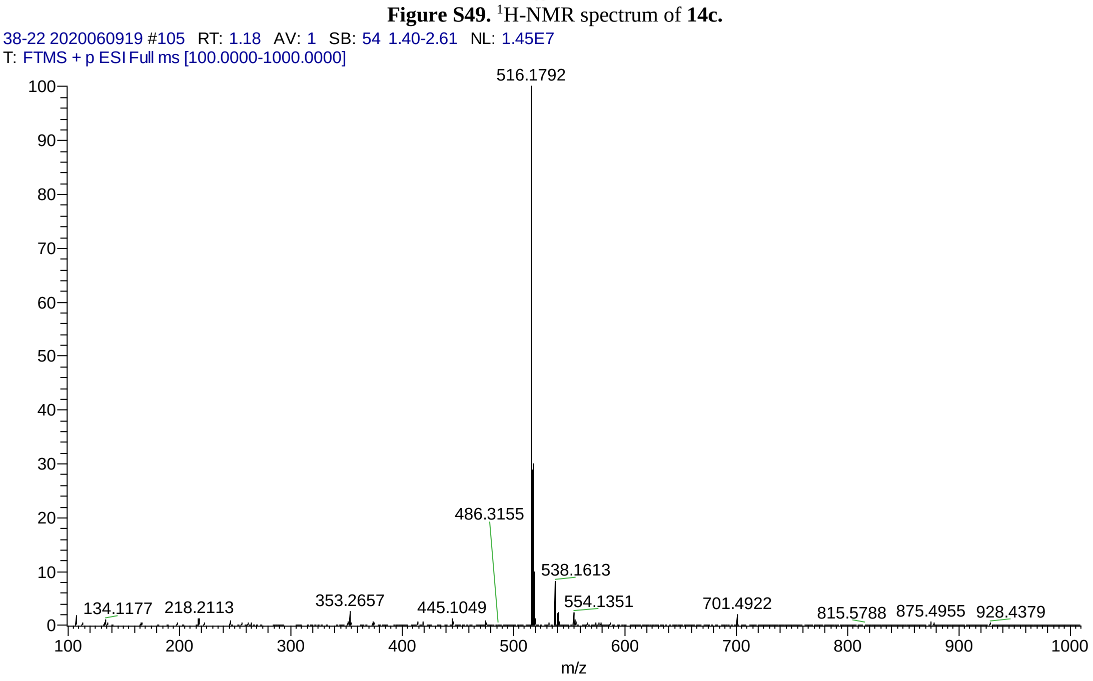

Figure S50. MS spectrum of **14c.**

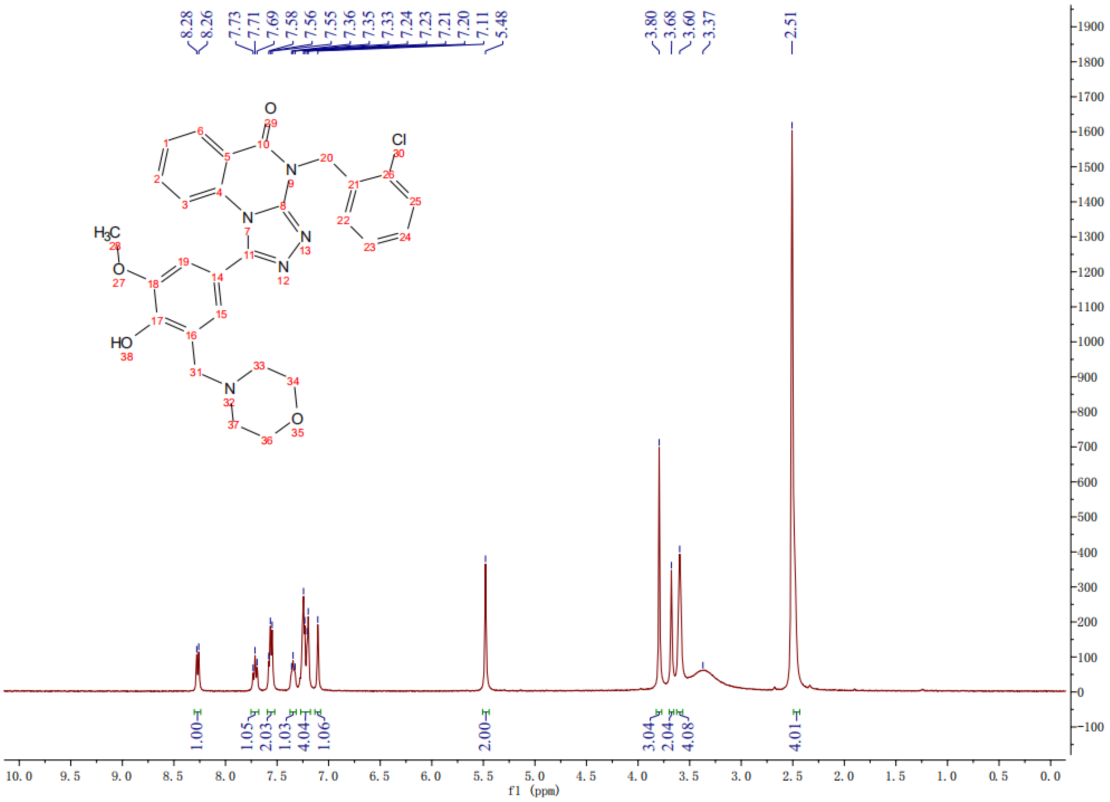

Figure S51. ^{1}H-NMR spectrum of **14d.**

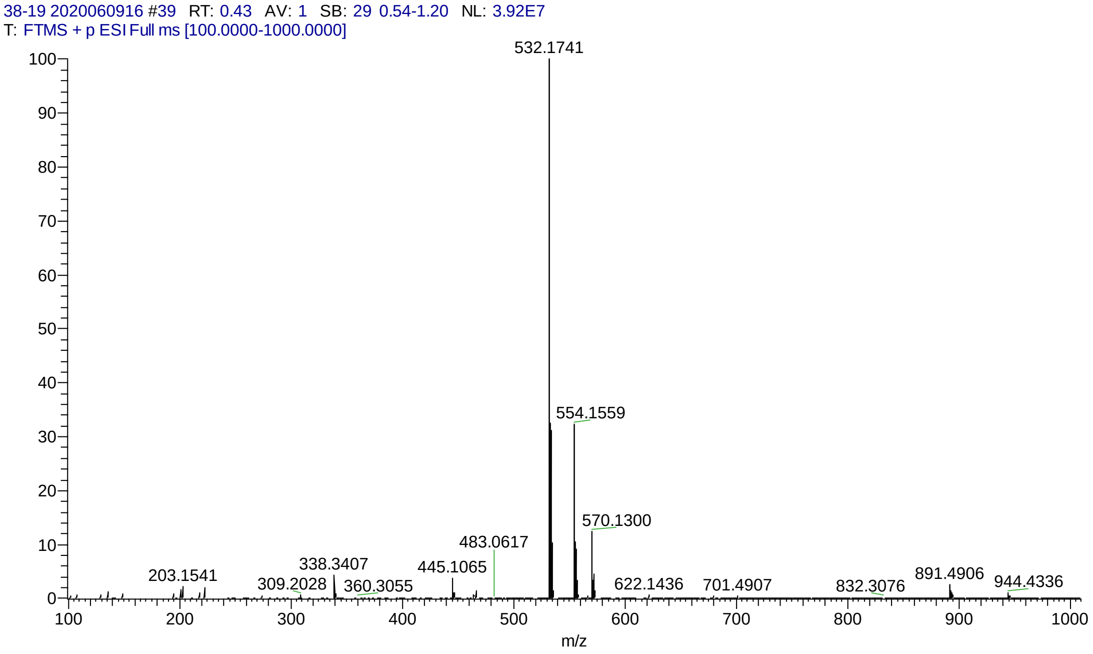

Figure S52. MS spectrum of **14d.**

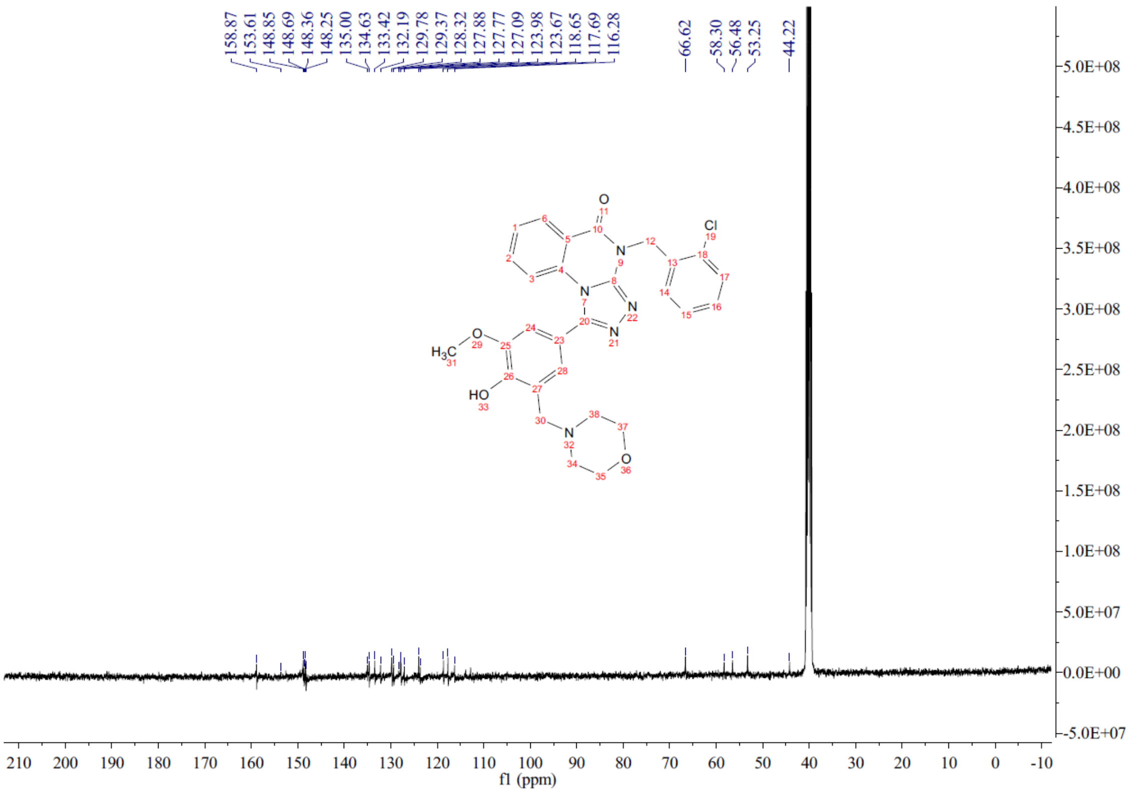

Figure S53.^{13}C-NMR spectrum of **14d**.

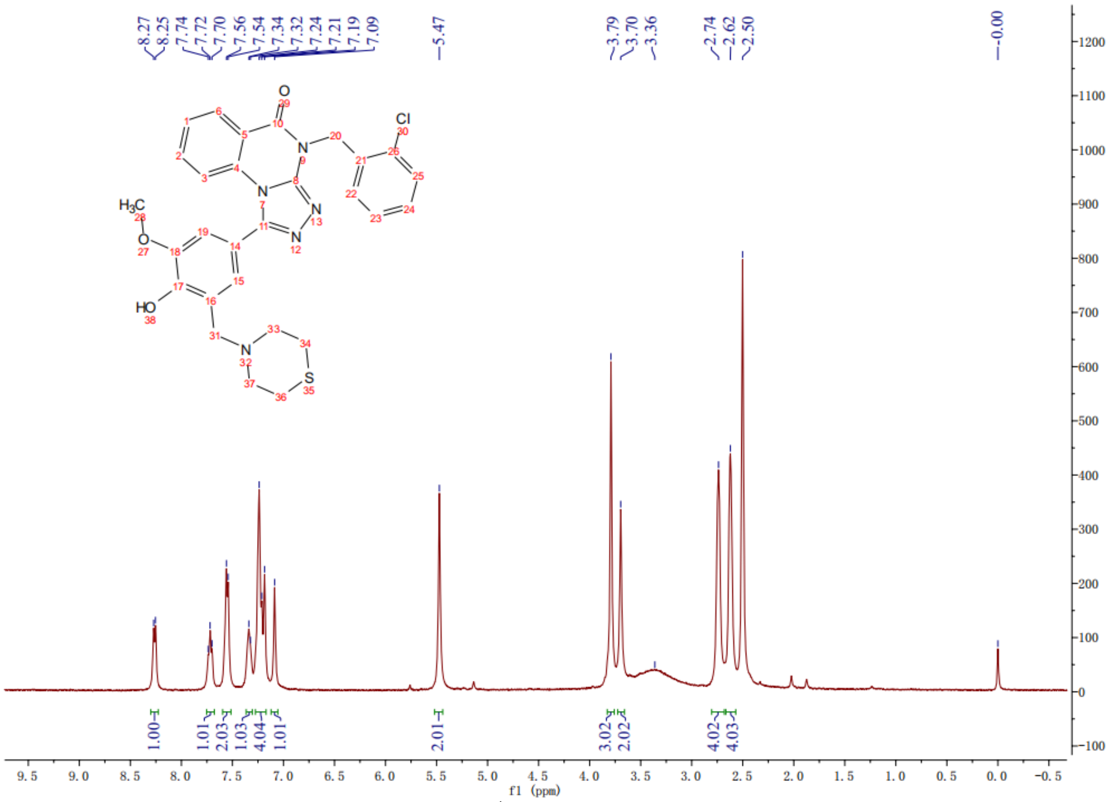

Figure S54. ^{1}H-NMR spectrum of **14e.**

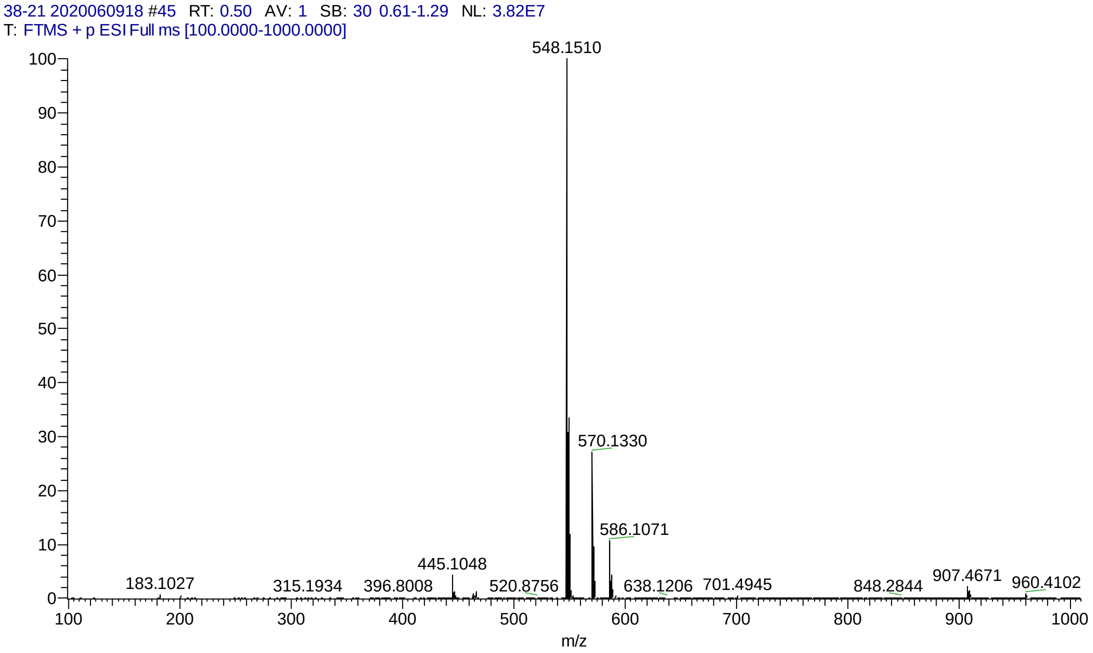

Figure S55. MS spectrum of **14e.**

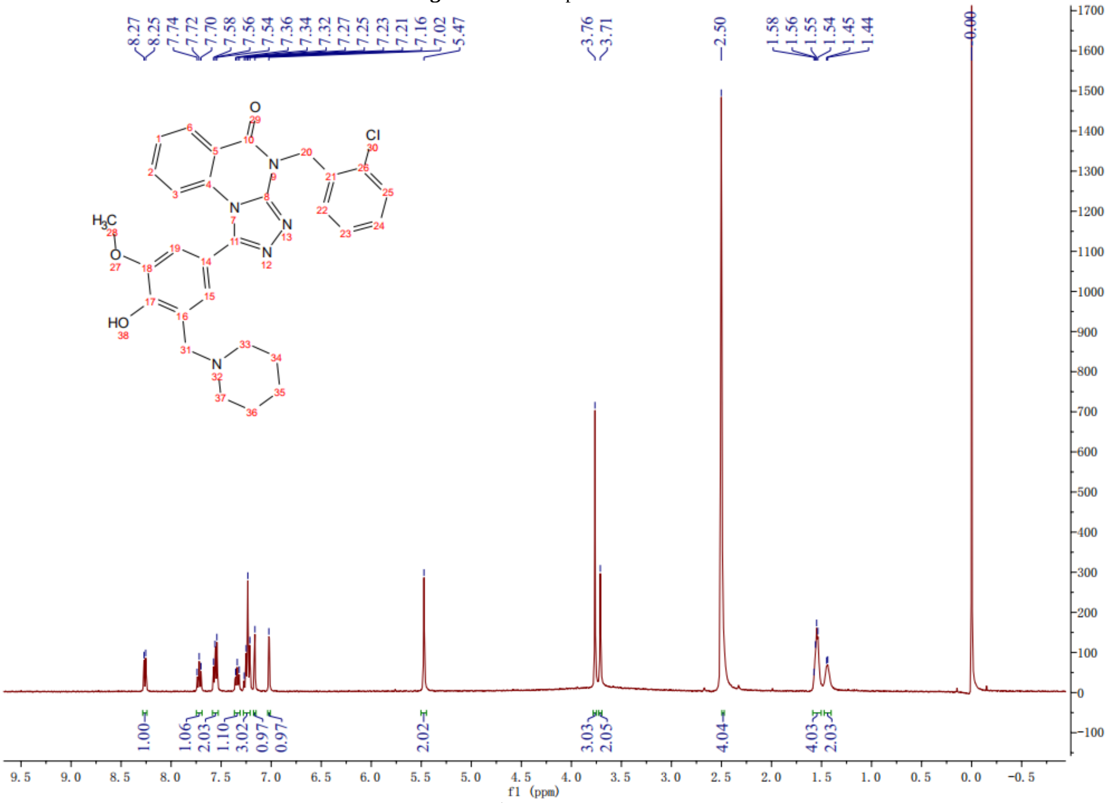

Figure S56. ^{1}H-NMR spectrum of **14f.**

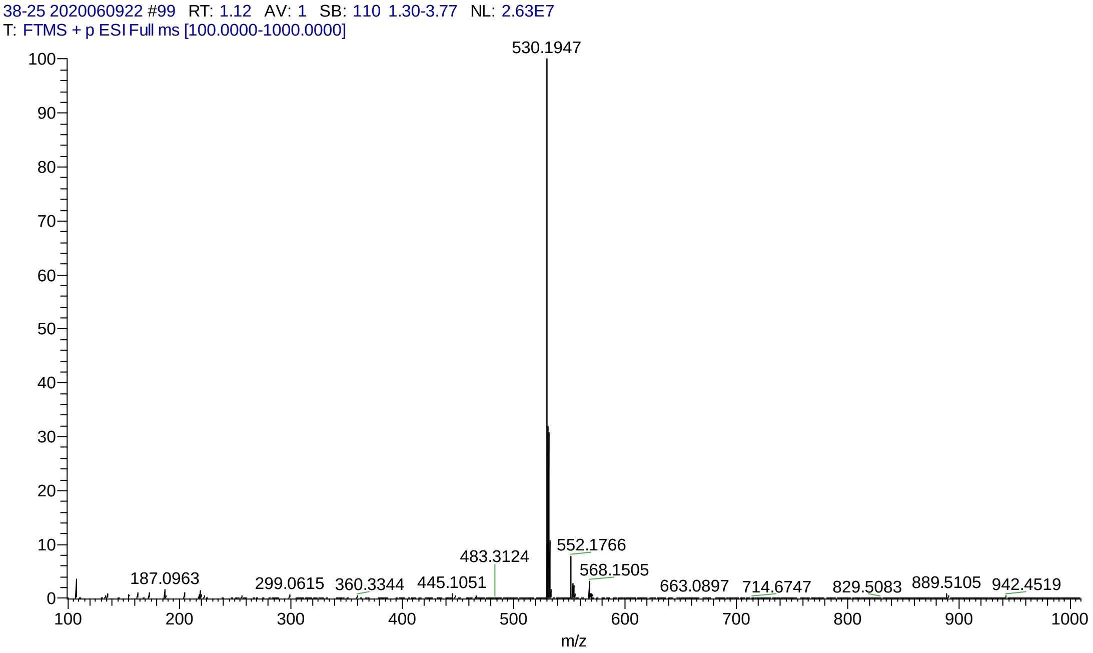

Figure S57. MS spectrum of **14f.**

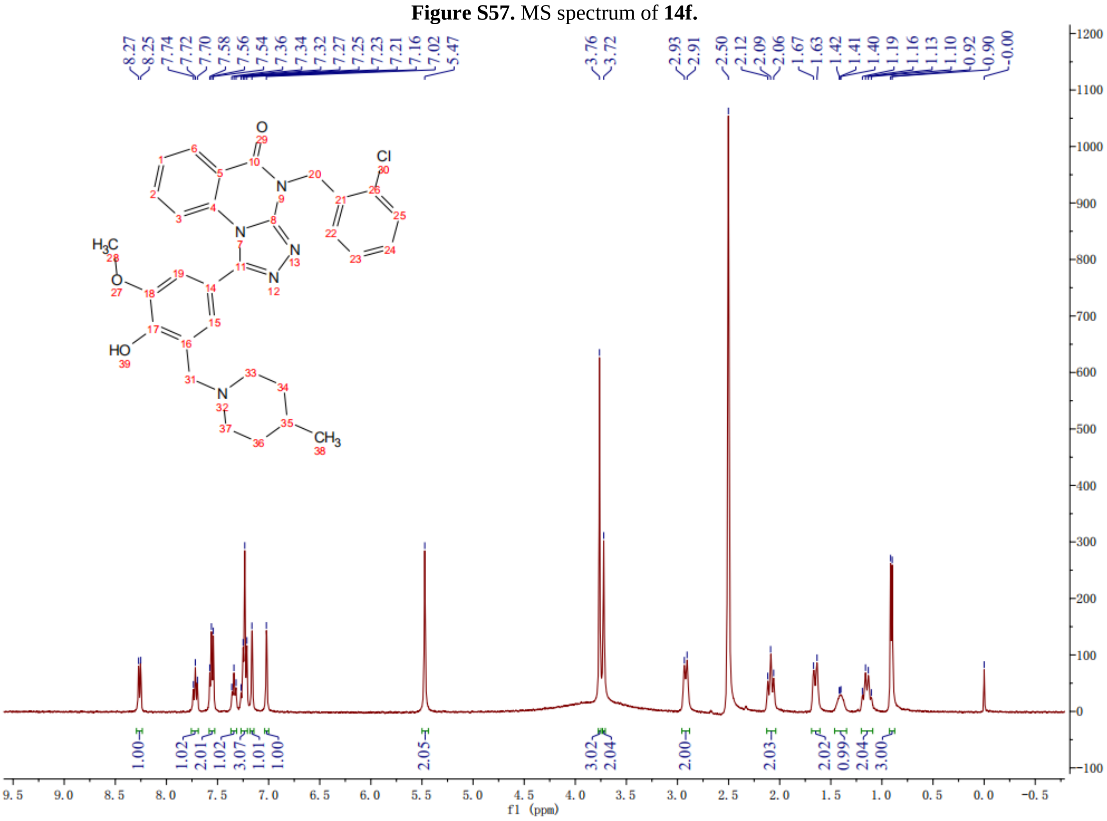

Figure S58. ^{1}H-NMR spectrum of **14g.**

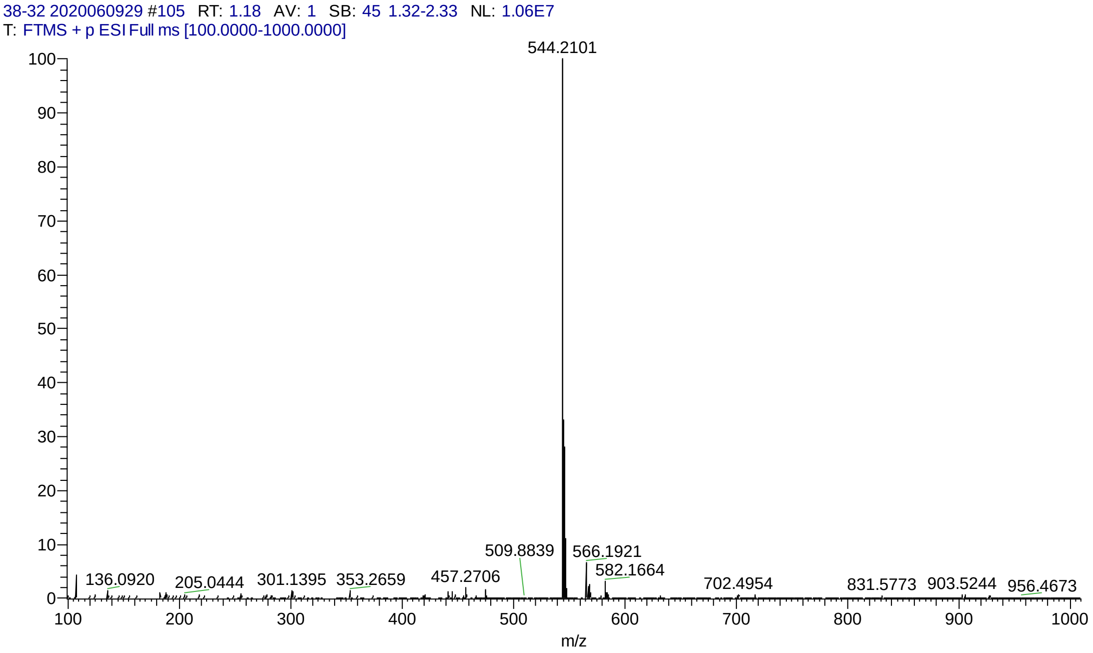

Figure S59. MS spectrum of **14g.**

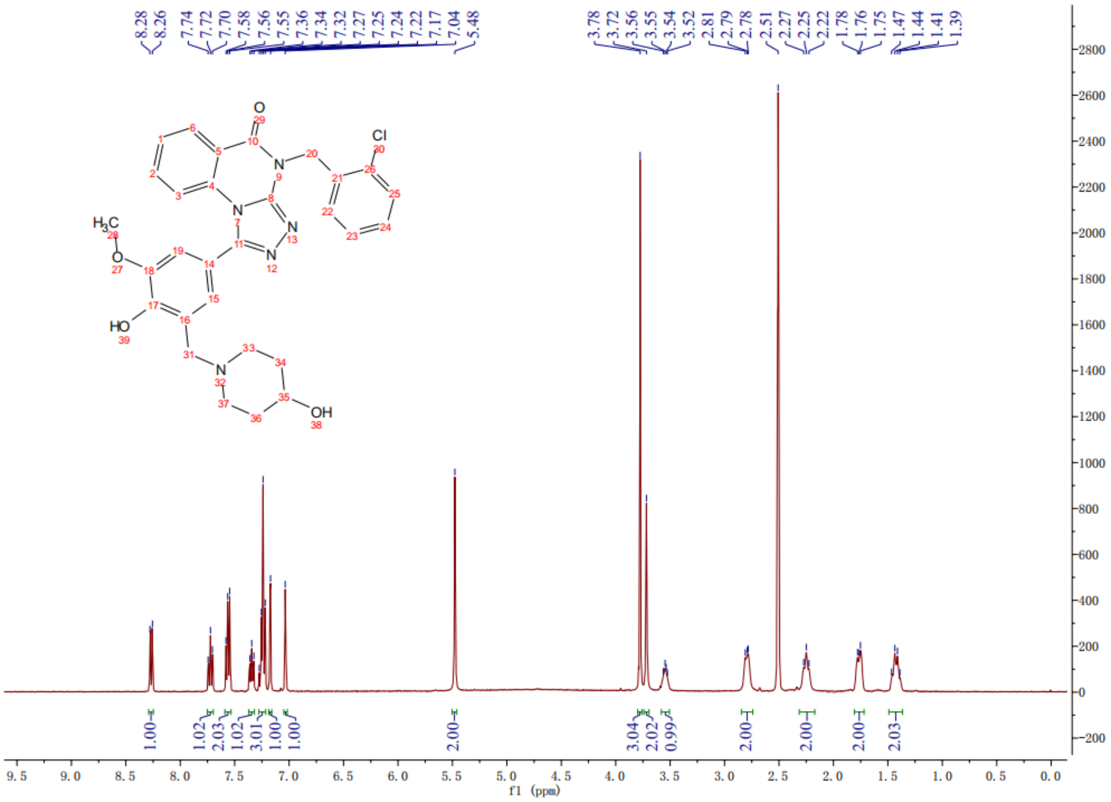

Figure S60. ^{1}H-NMR spectrum of **14h.**

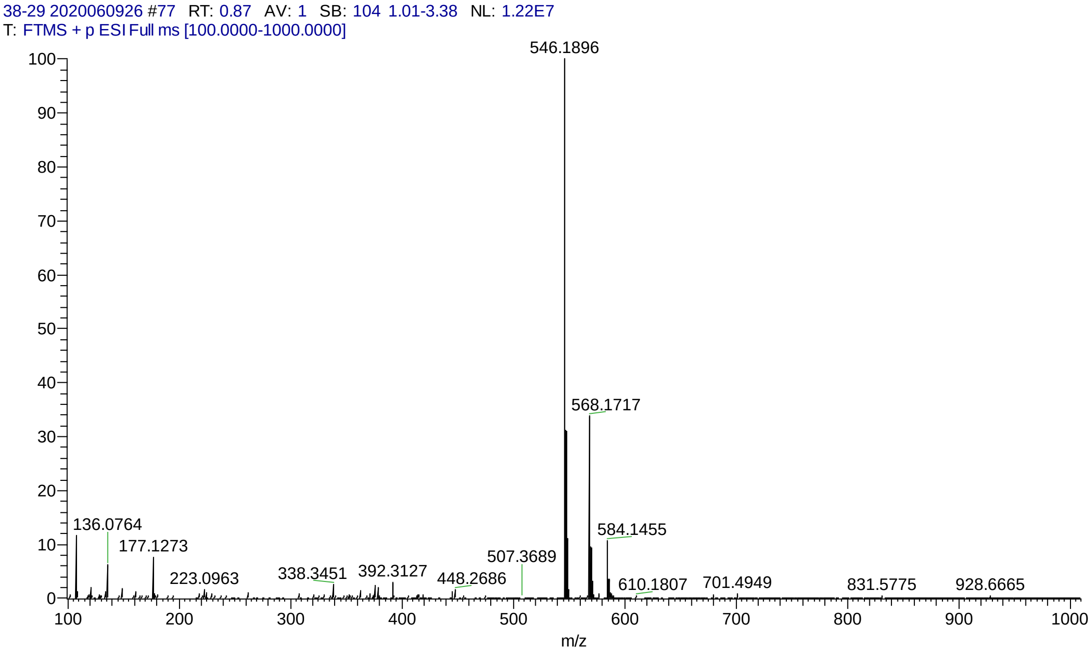

Figure S61. MS spectrum of **14h.**

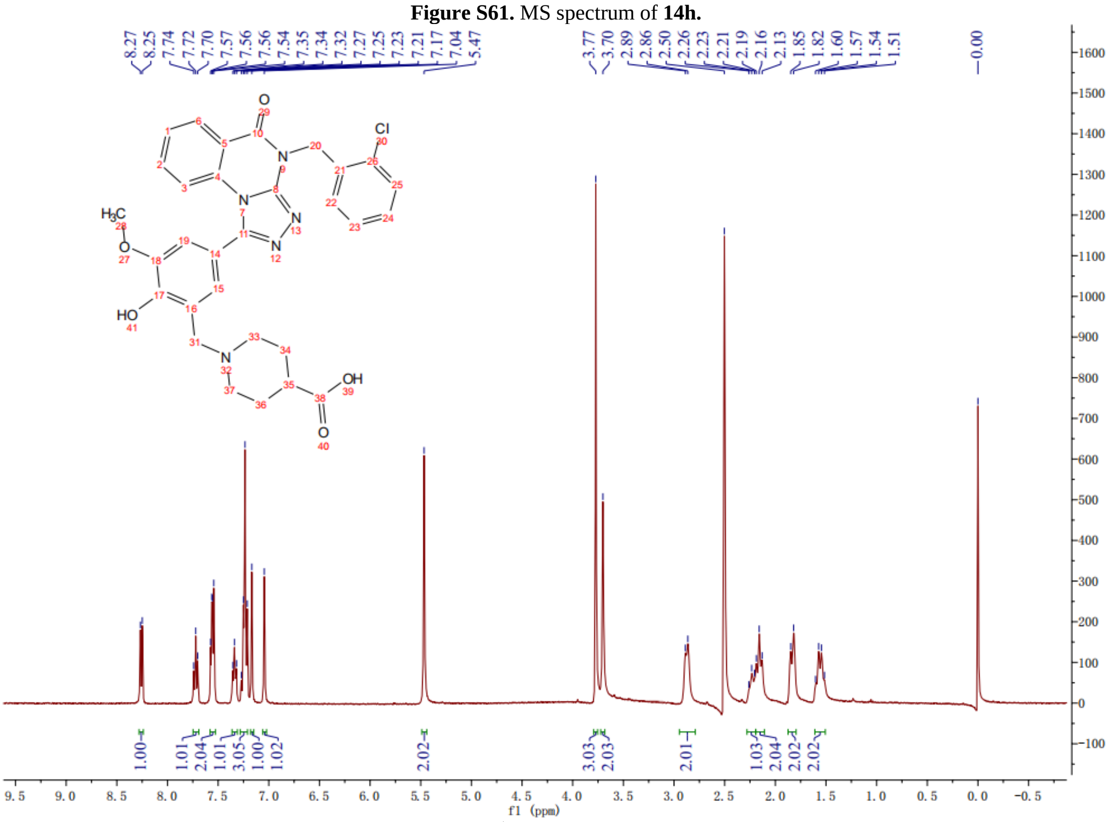

Figure S62. ^{1}H-NMR spectrum of **14i.**

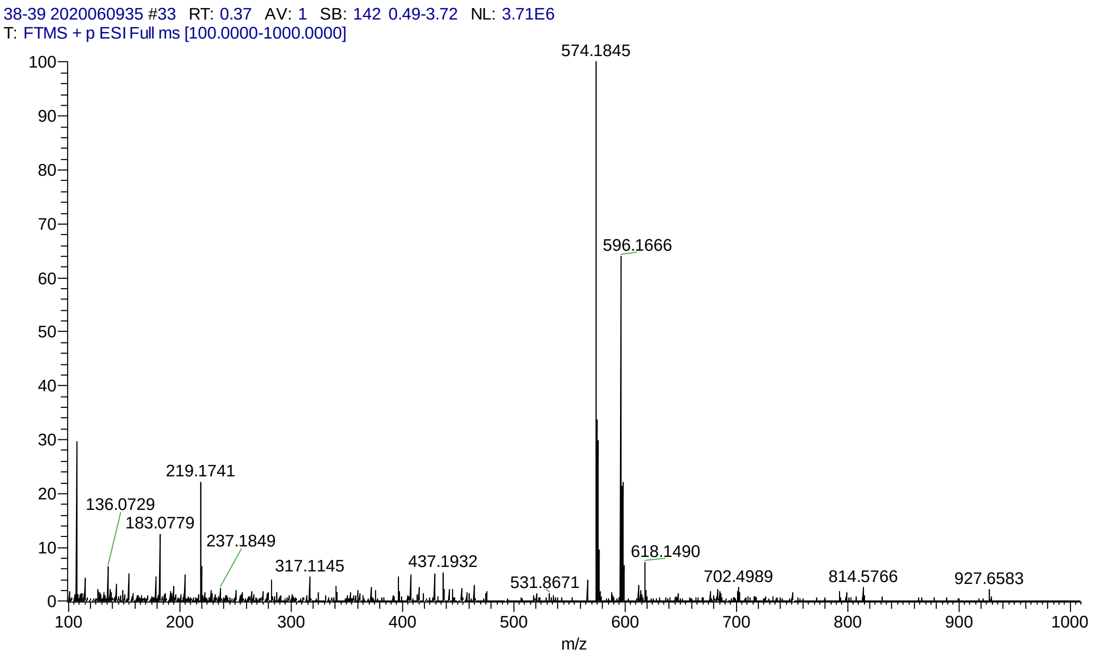

Figure S63. MS spectrum of **14i.**

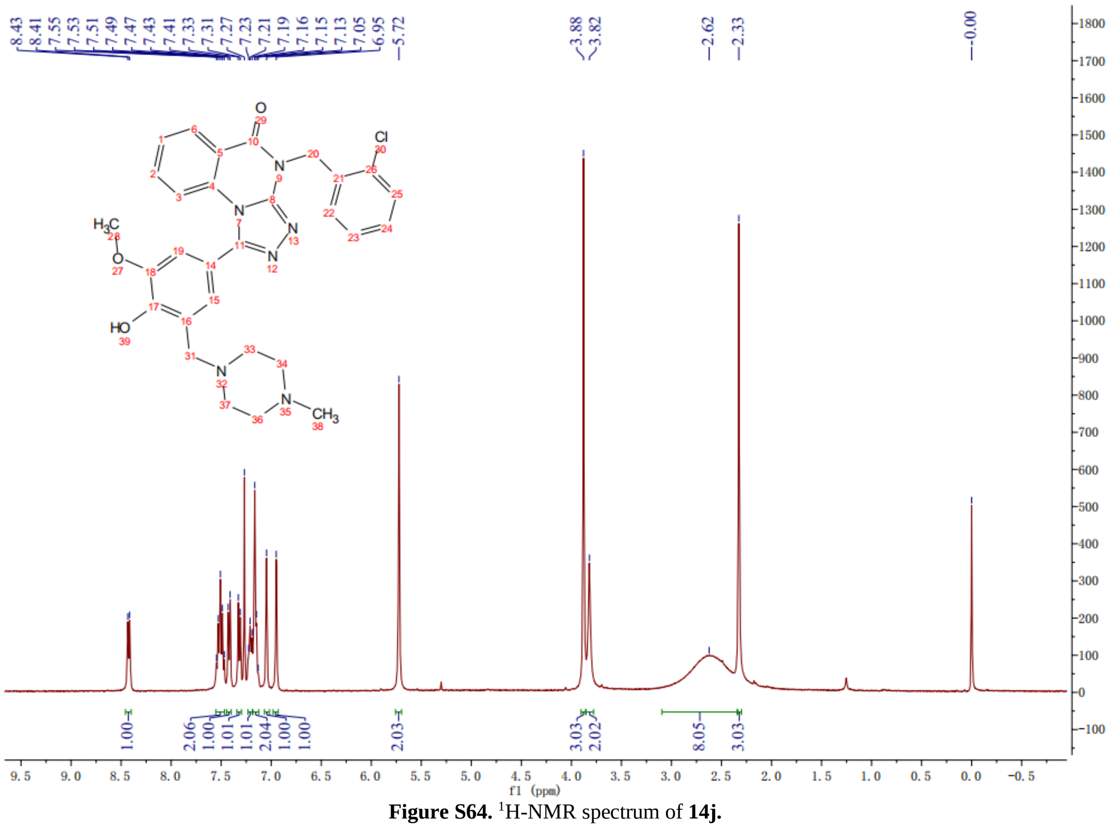

Figure S64. ^{1}H-NMR spectrum of **14j.**

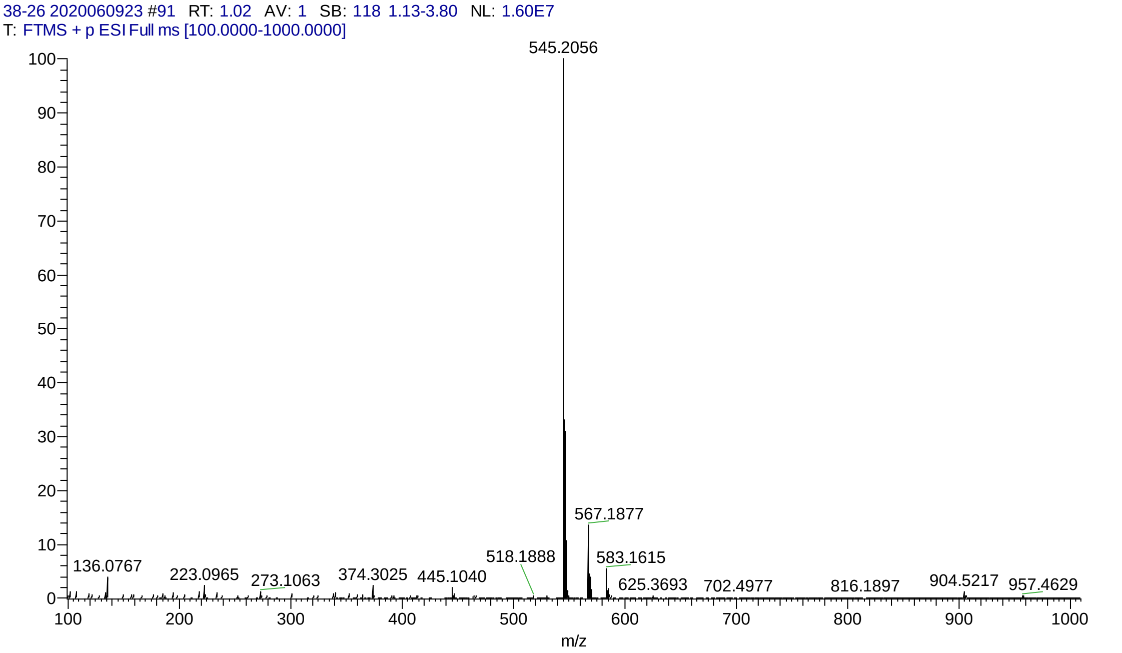

Figure S65. MS spectrum of **14j.**

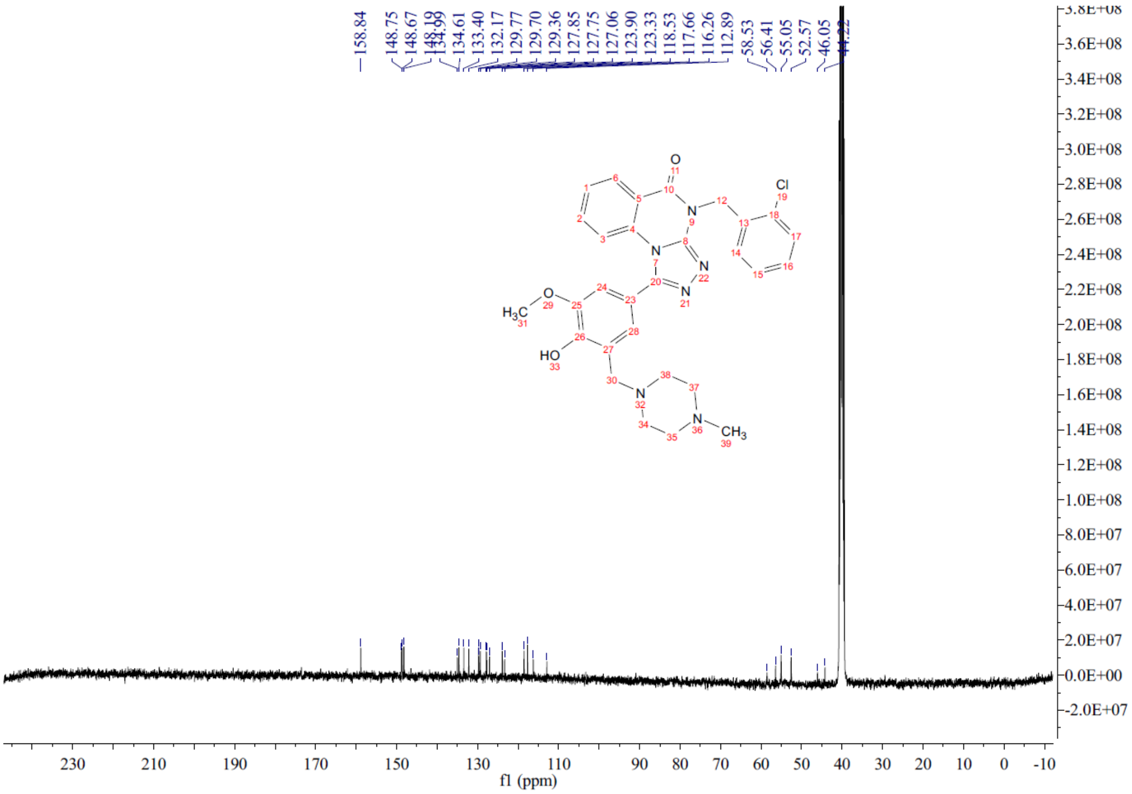

Figure S66.^{13}C-NMR spectrum of **14j**.

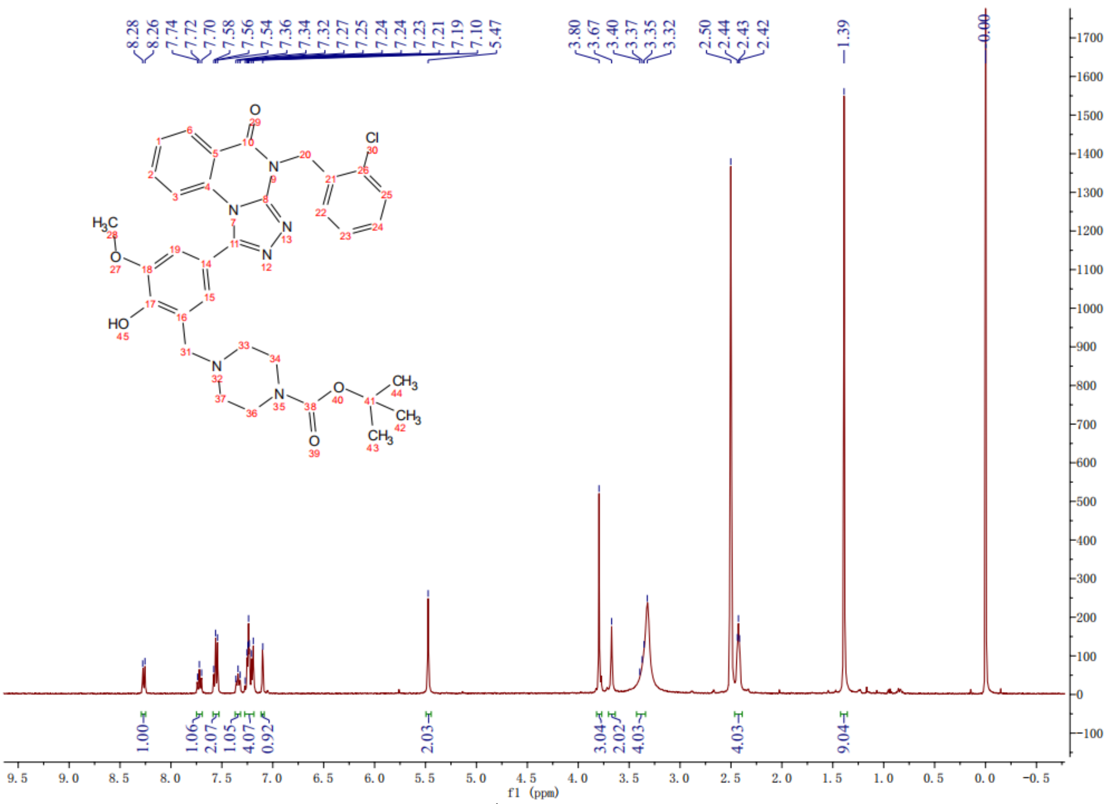

Figure S67. ^{1}H-NMR spectrum of **14k.**

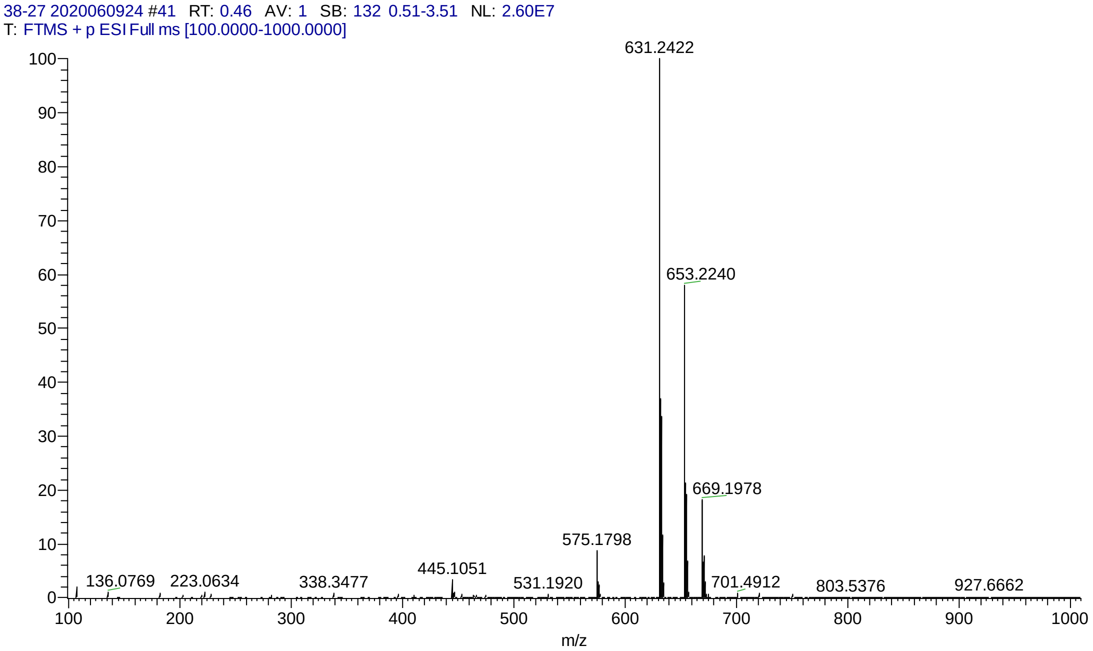

Figure S68. MS spectrum of **14k.**

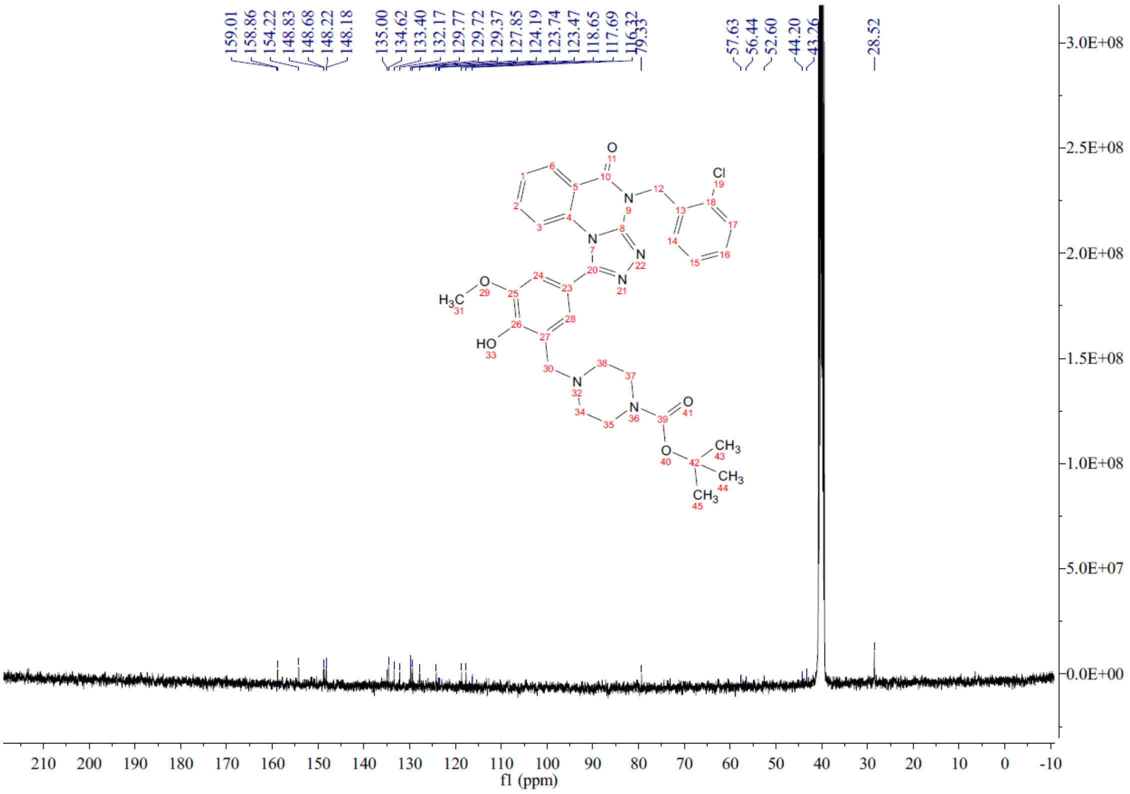

Figure S69.^{13}C-NMR spectrum of **14k**.

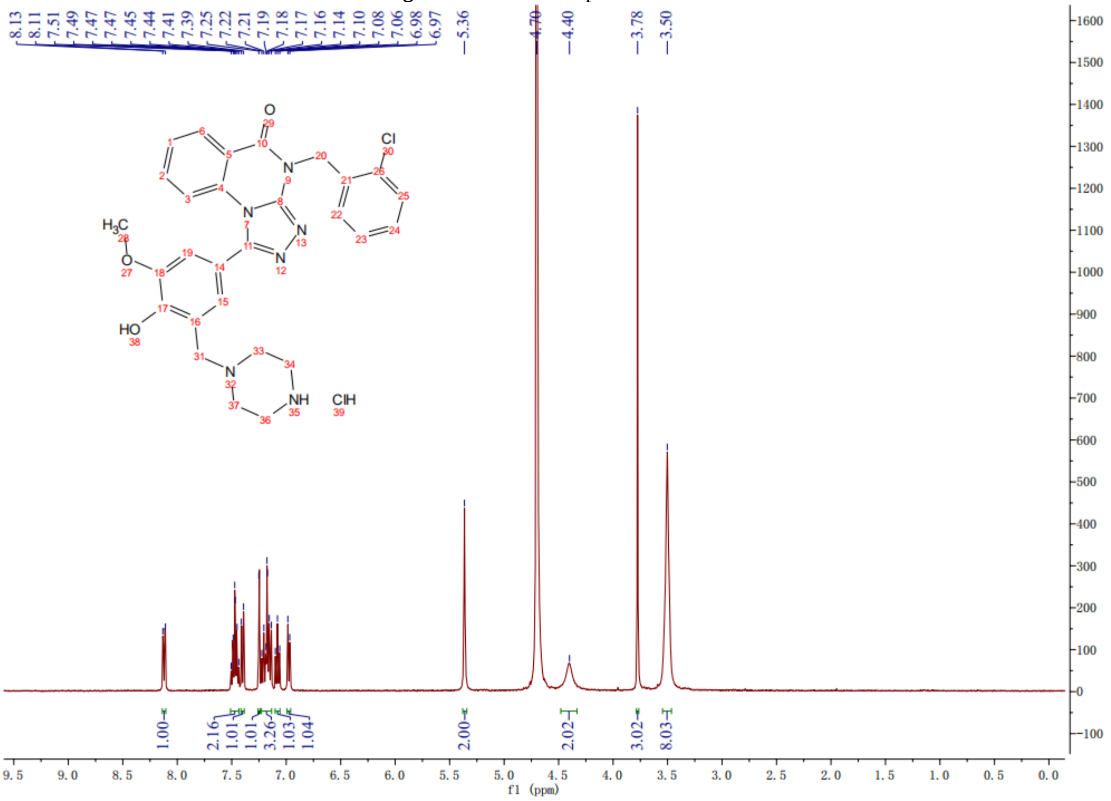

Figure S70. ^{1}H-NMR spectrum of **14l.**

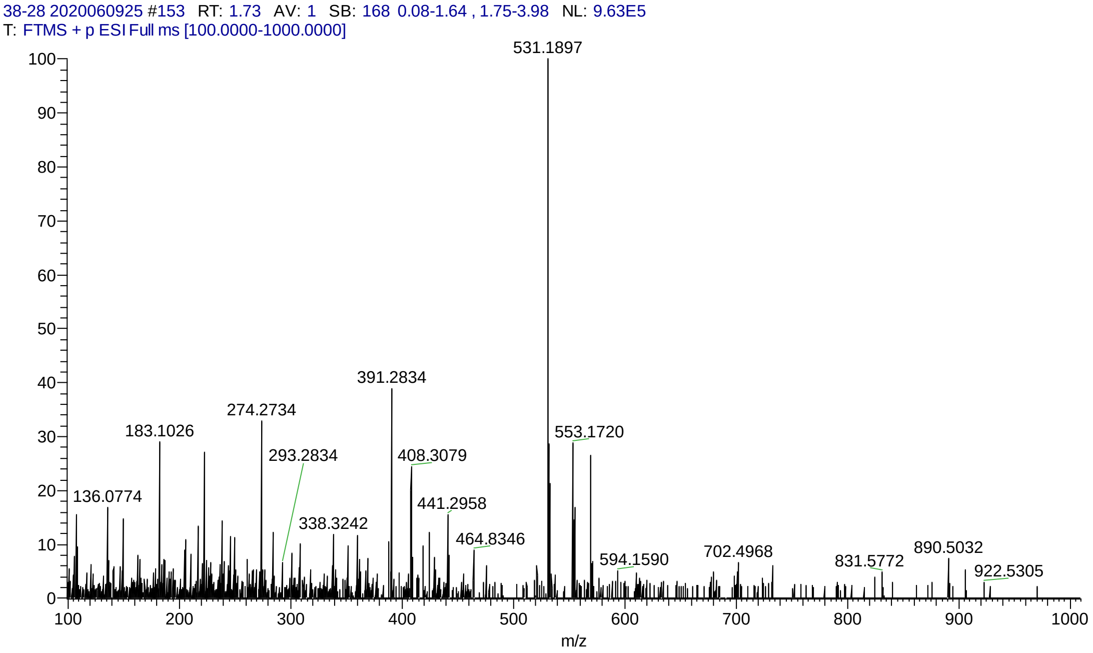

Figure S71. MS spectrum of **14l.**

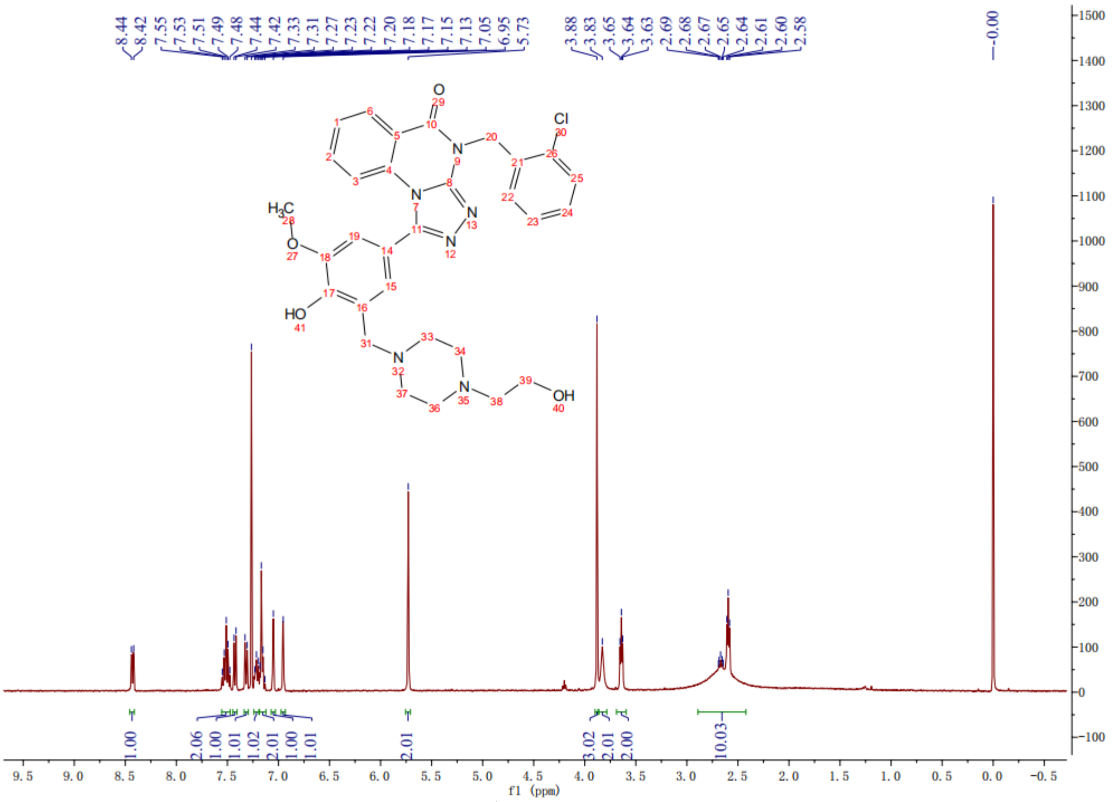

Figure S72. ^{1}H-NMR spectrum of **14m.**

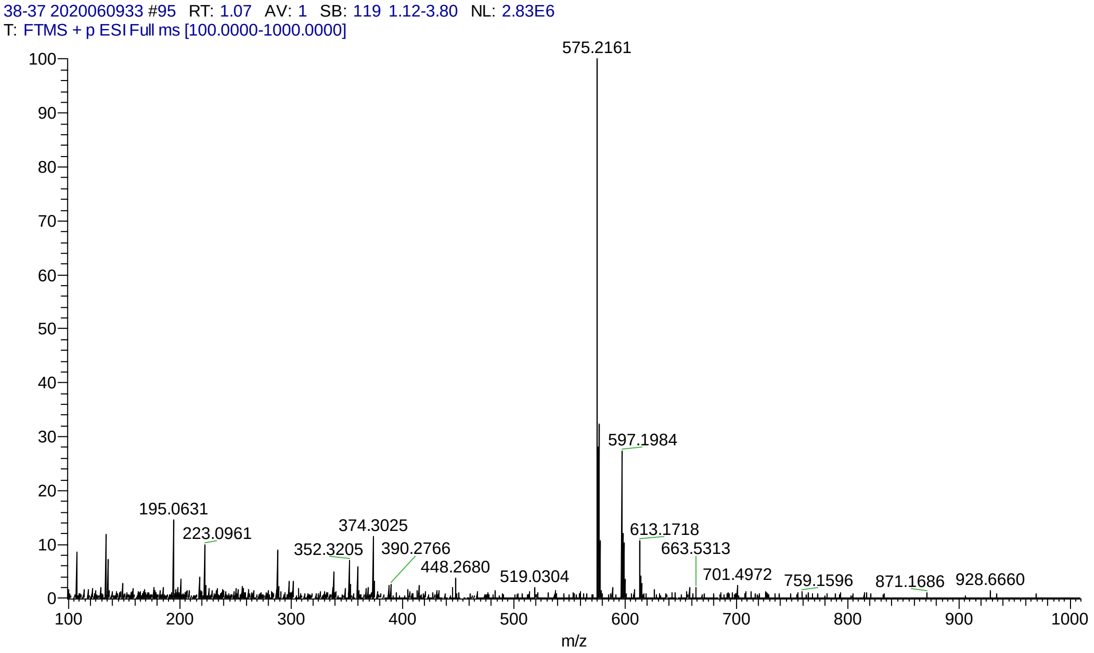

Figure S73. MS spectrum of **14m.**

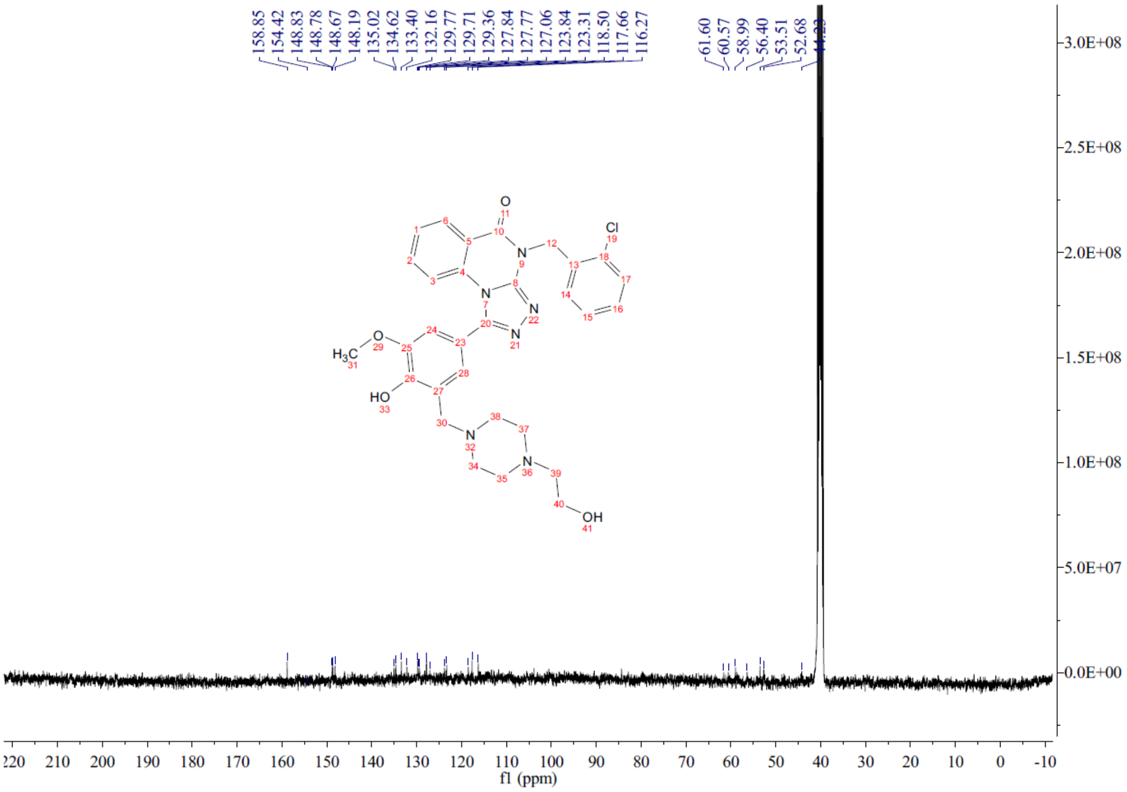

Figure S74.^{13}C-NMR spectrum of **14m**.

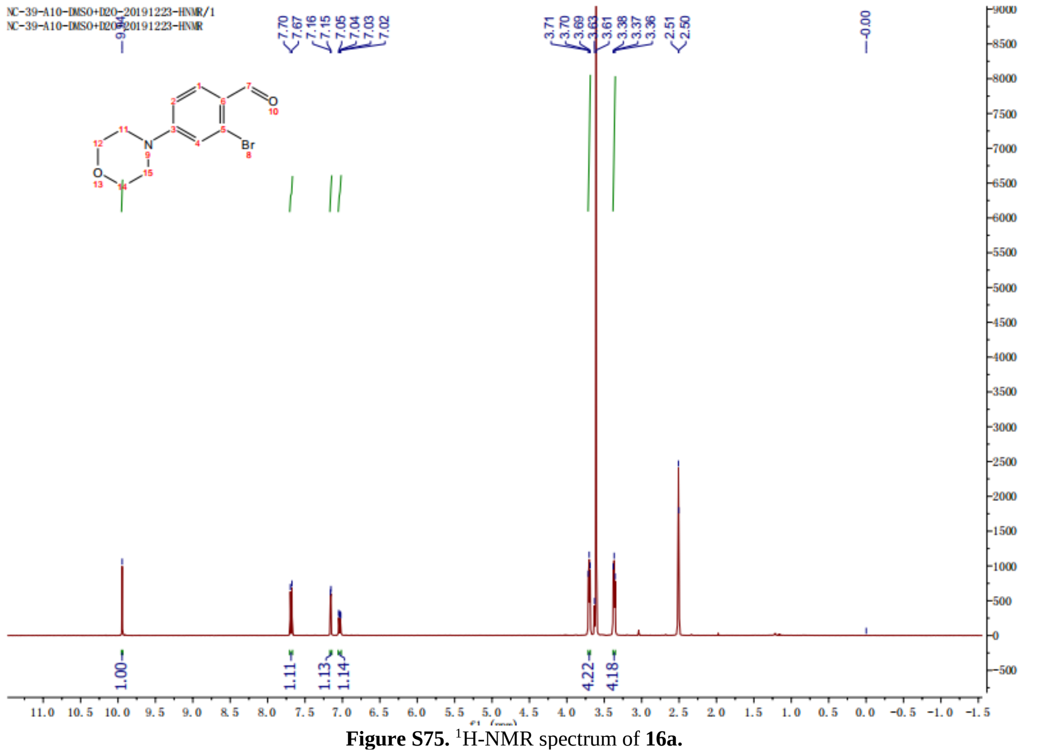

Figure S75. ¹H-NMR spectrum of **16a.**

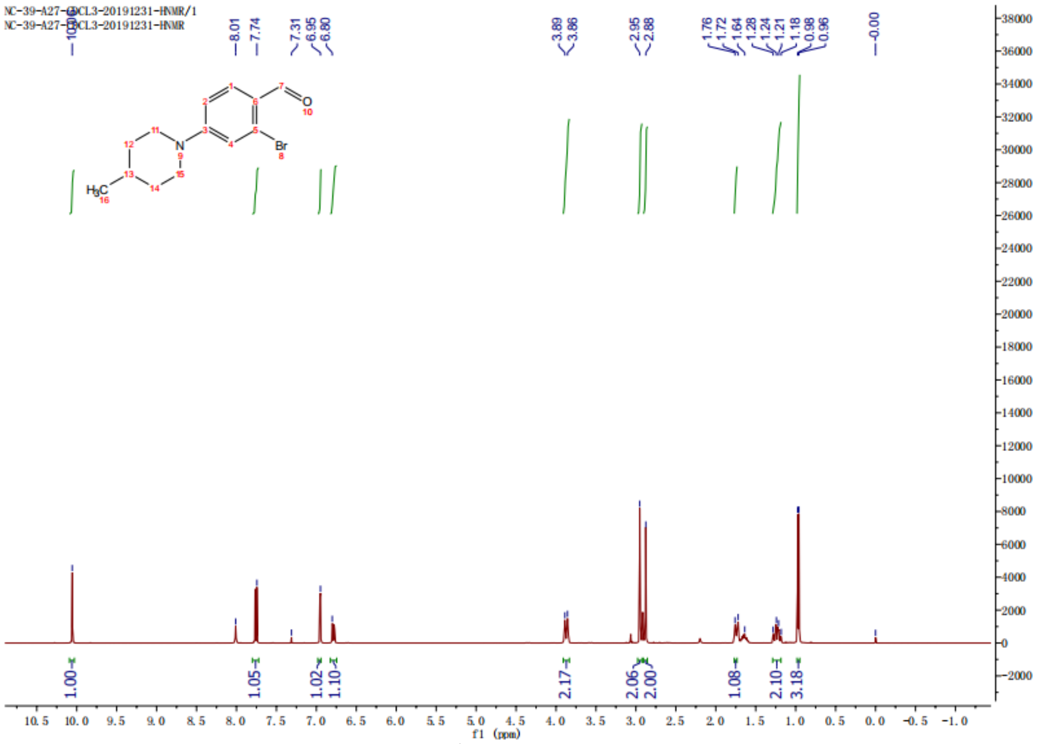

Figure S76. ¹H-NMR spectrum of **16b.**

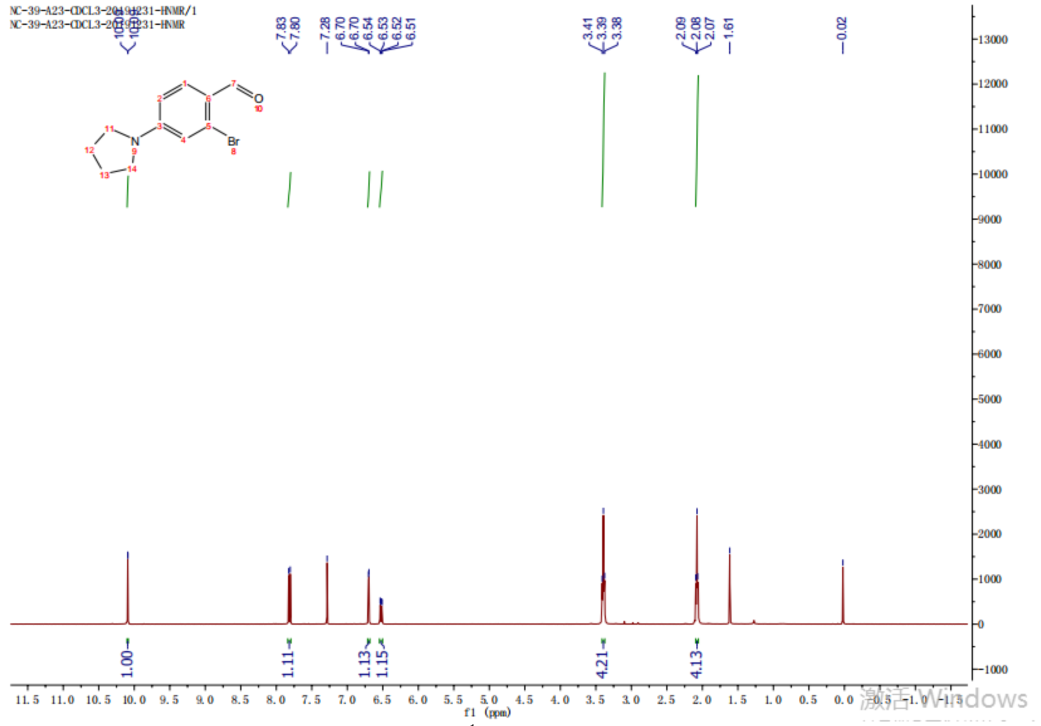

Figure S77. ^{1}H-NMR spectrum of **16c.**

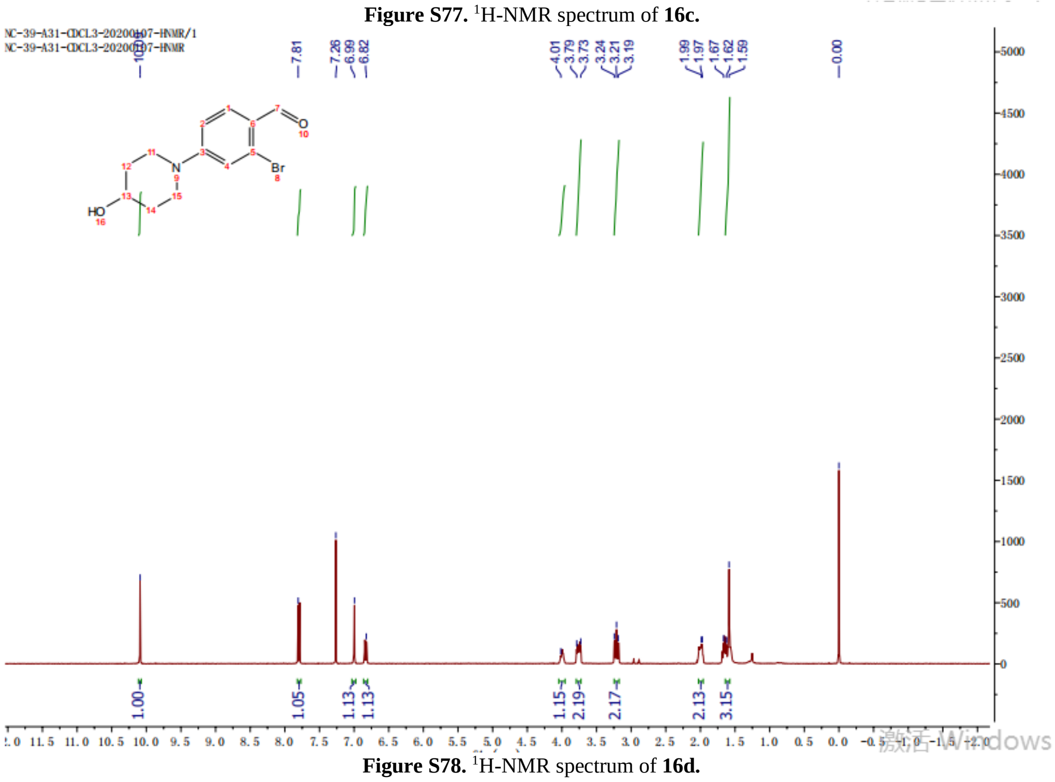

Figure S78. ^{1}H-NMR spectrum of **16d.**

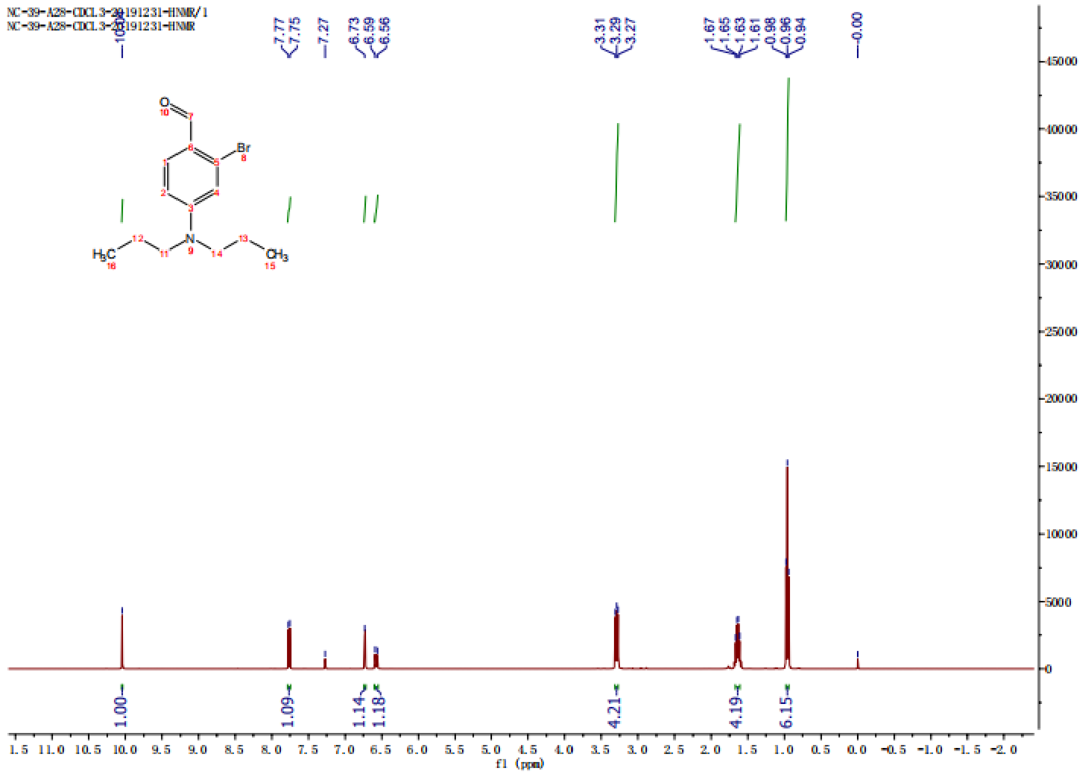

Figure S79. ^{1}H-NMR spectrum of **16e.**

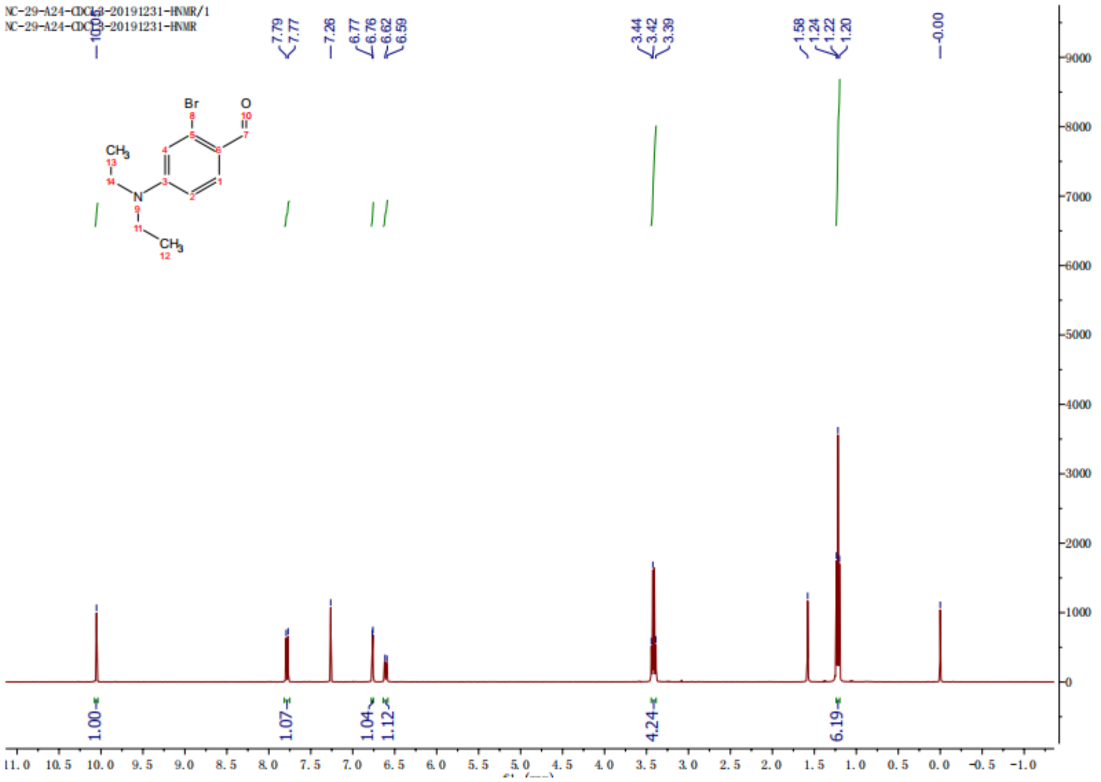

Figure S80. ^{1}H-NMR spectrum of **16f.**

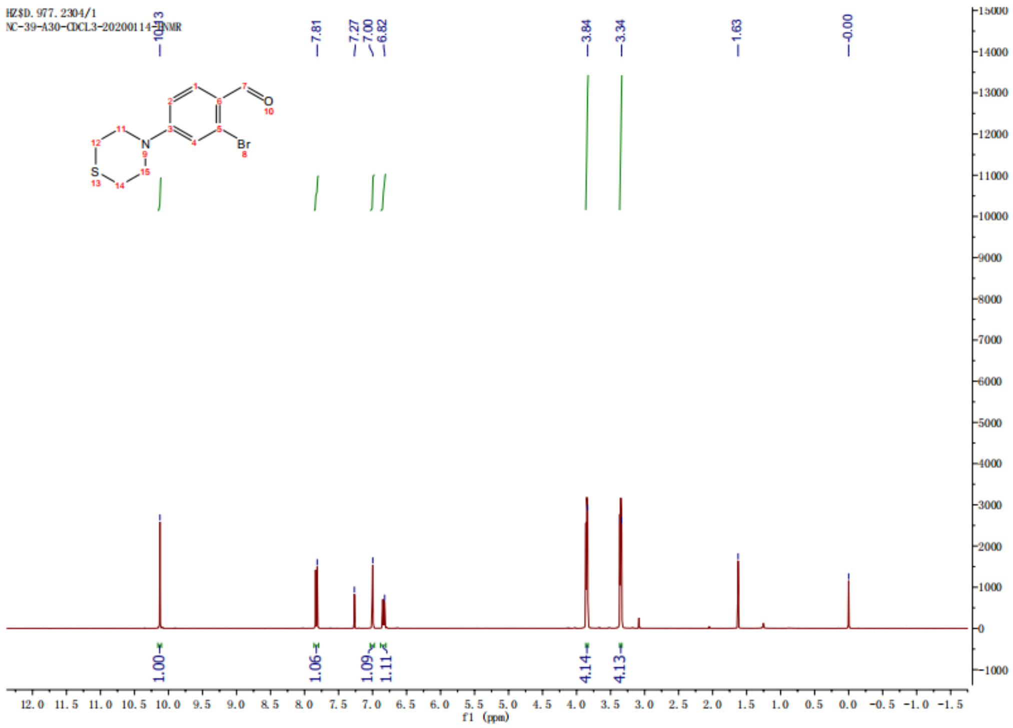

Figure S81. ^{1}H-NMR spectrum of **16g.**

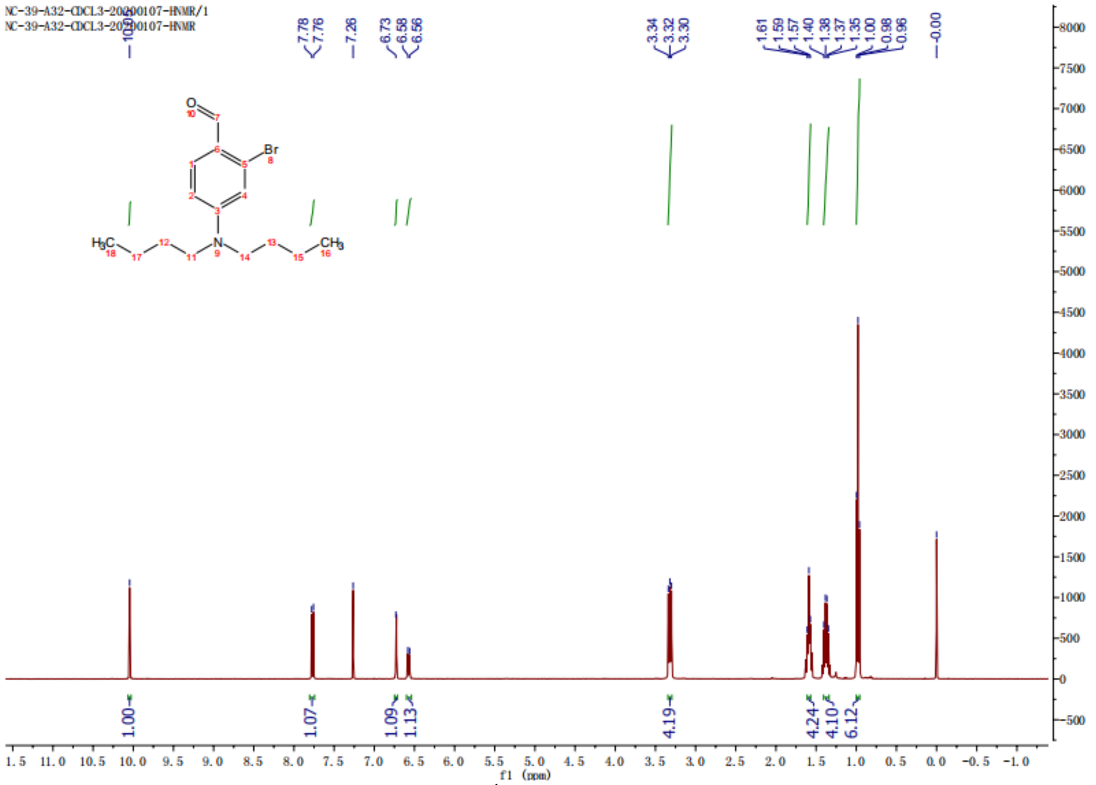

Figure S82. ^{1}H-NMR spectrum of **16h.**

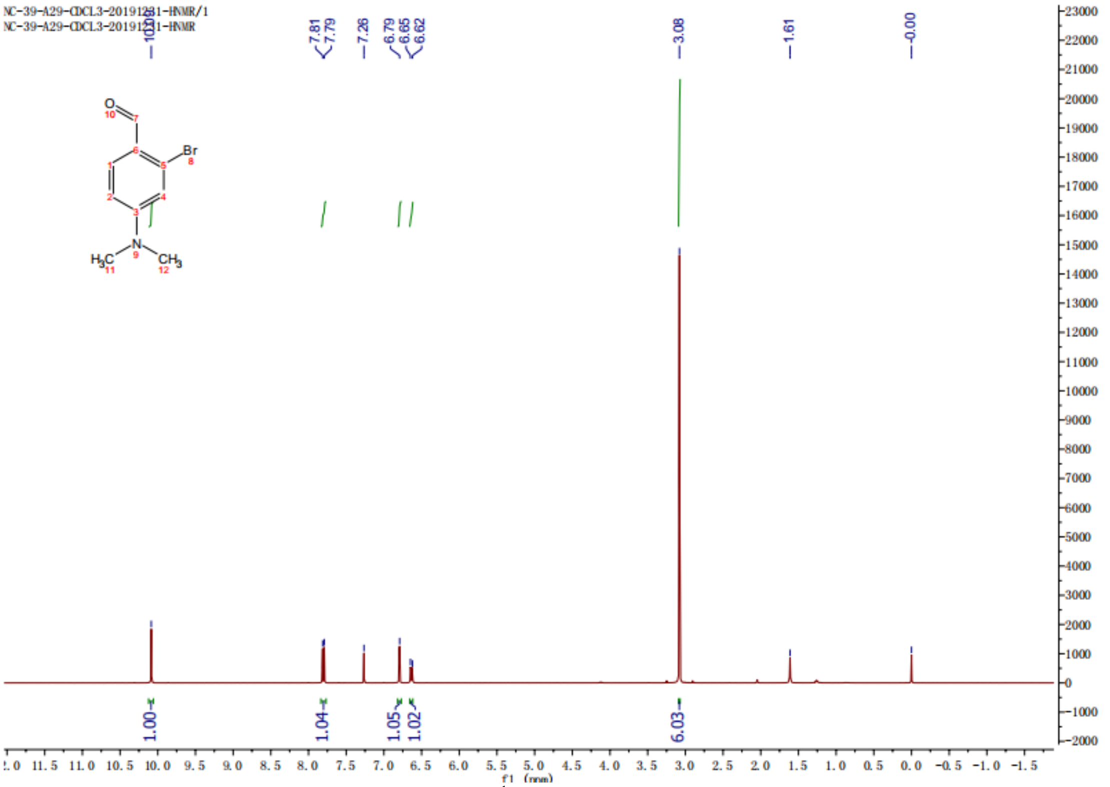

Figure S83. ^{1}H-NMR spectrum of **16i.**

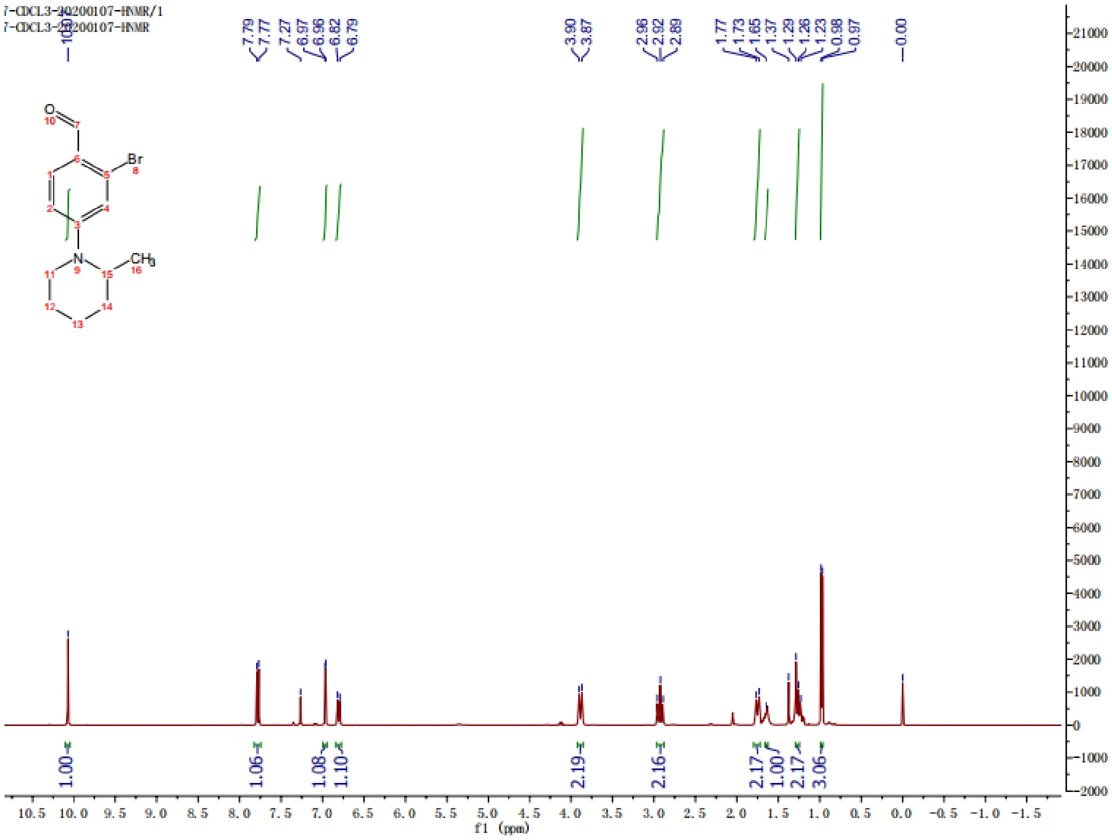

Figure S84. ^{1}H-NMR spectrum of **16j.**

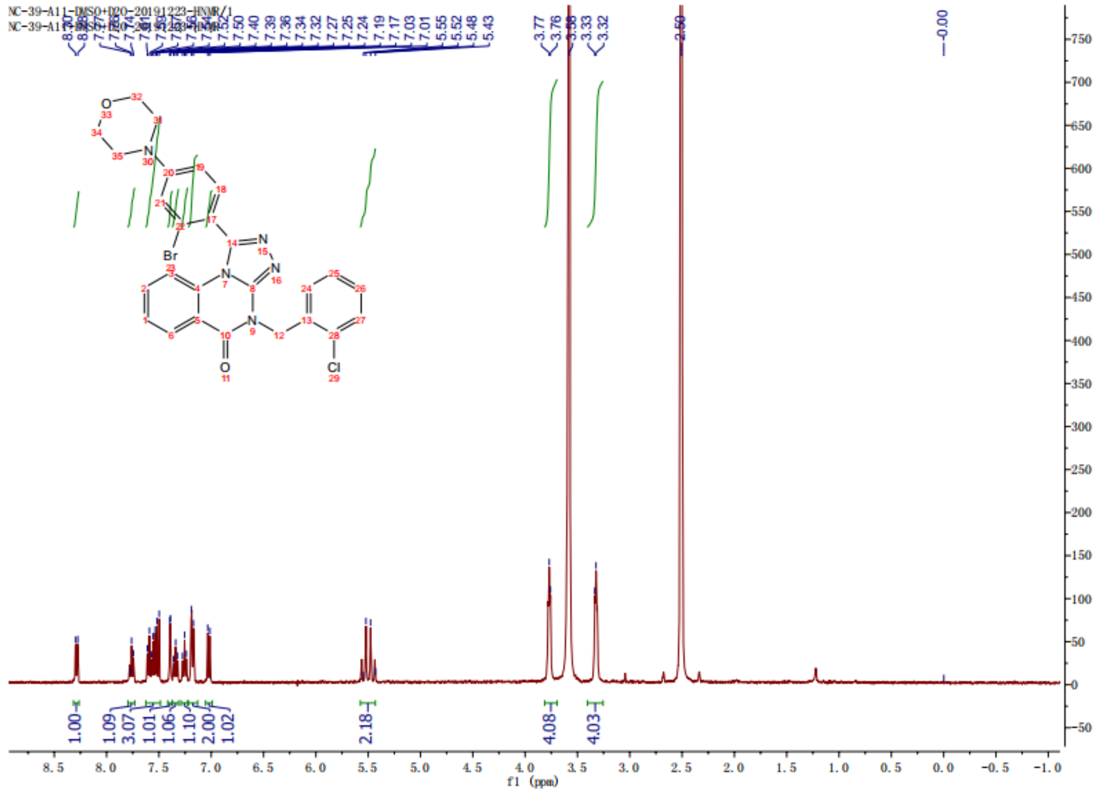

Figure S85. ^{1}H-NMR spectrum of **17a.**

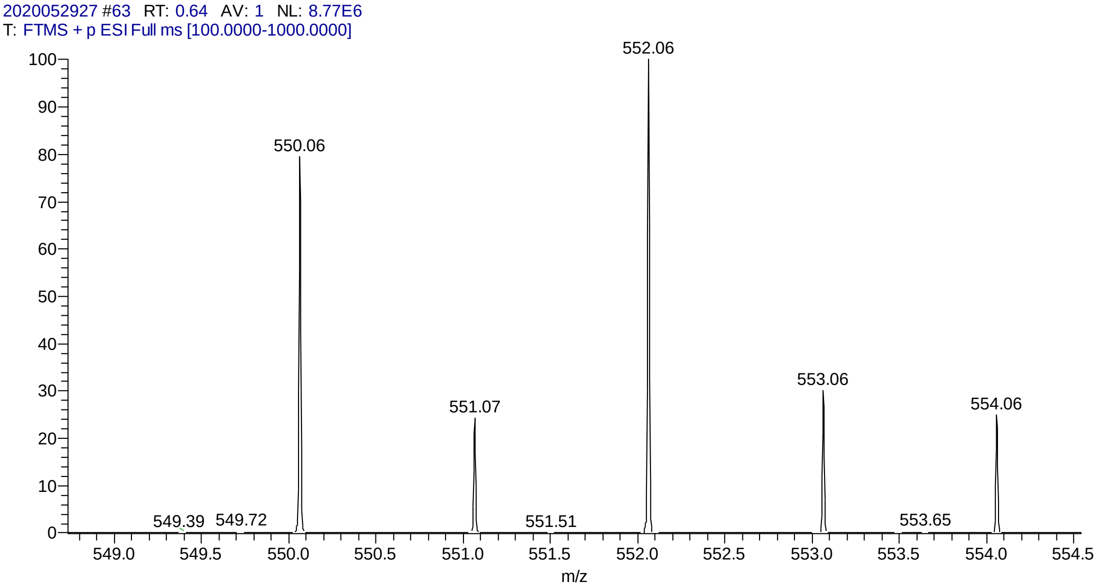

Figure S86. MS spectrum of **17a.**

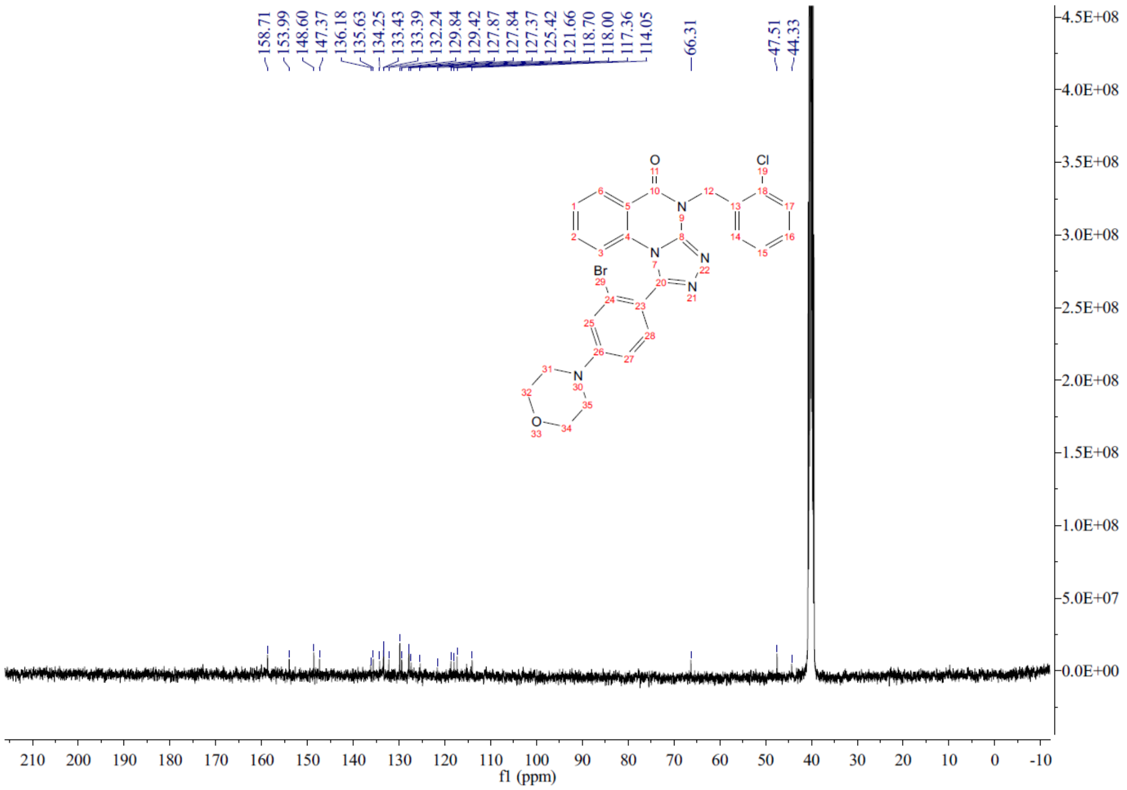

Figure S87.^{13}C-NMR spectrum of **17a**.

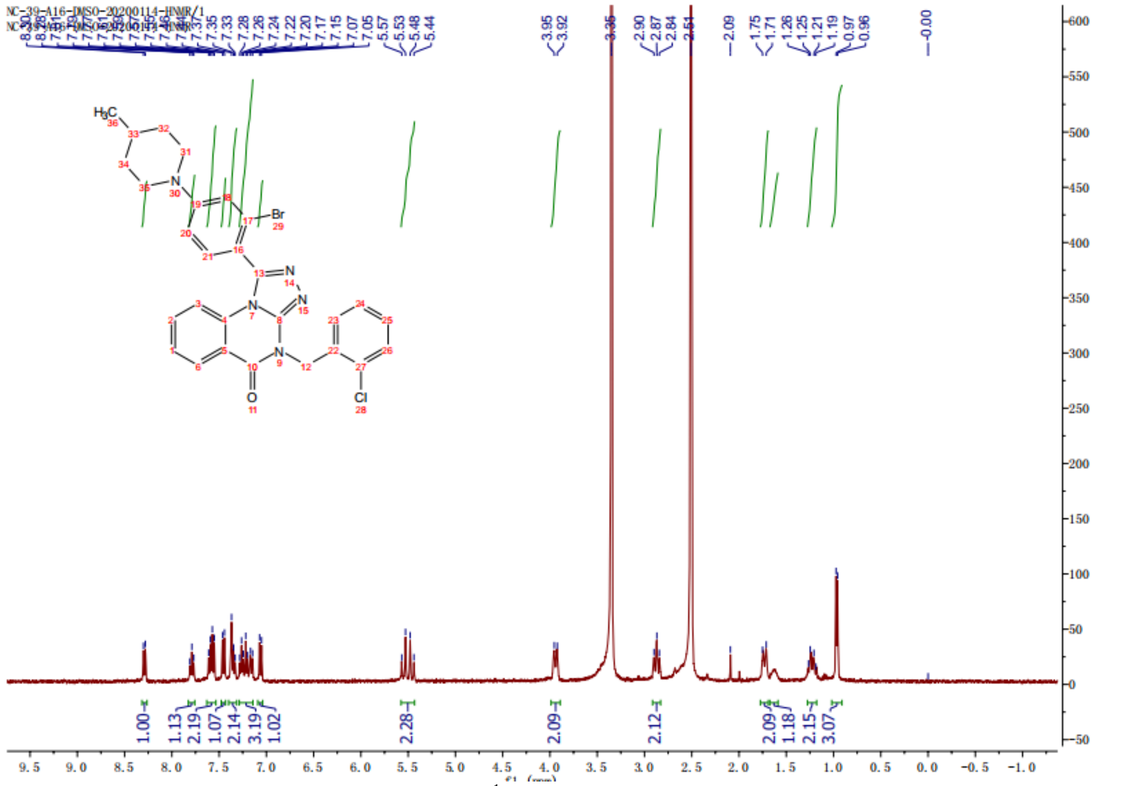

Figure S88. ^{1}H-NMR spectrum of **17b.**

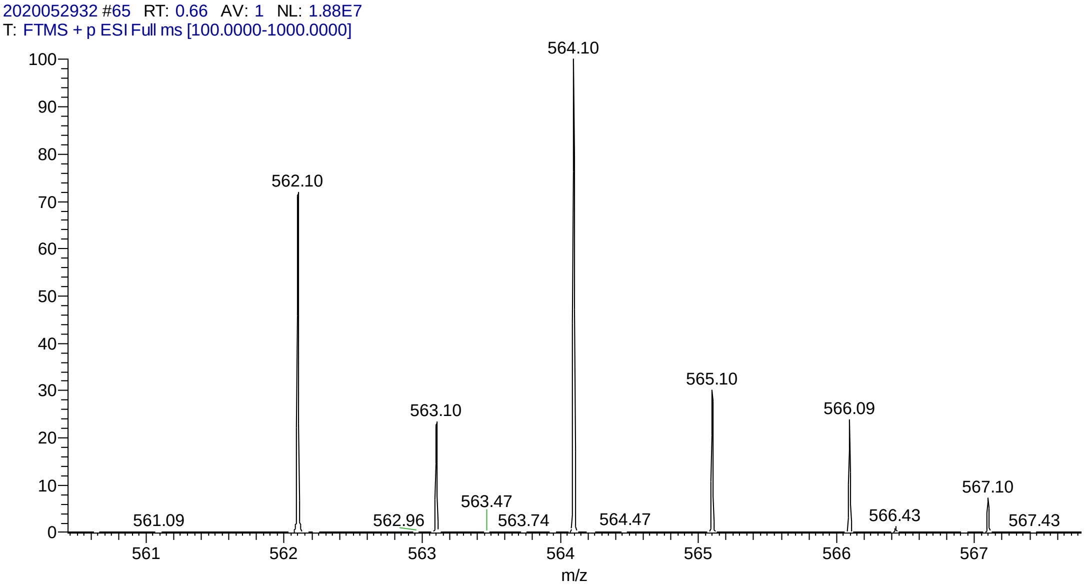

Figure S89. MS spectrum of **17b.**

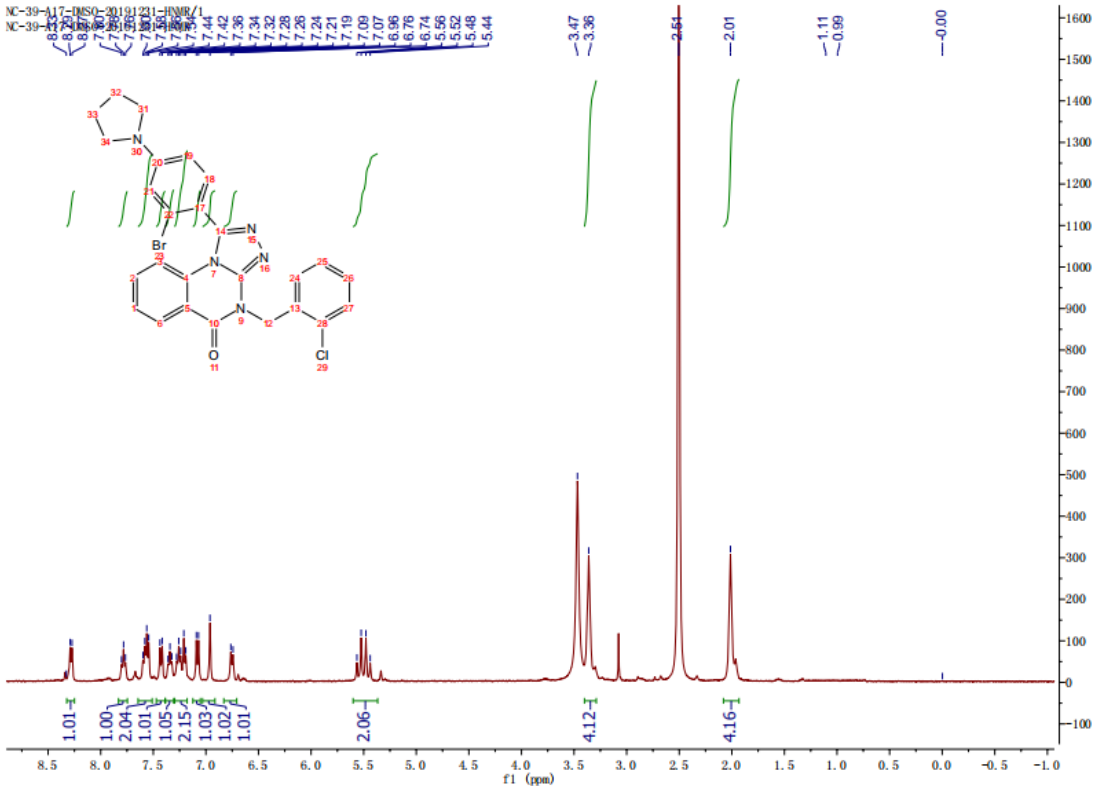

Figure S90. ^{1}H-NMR spectrum of **17c.**

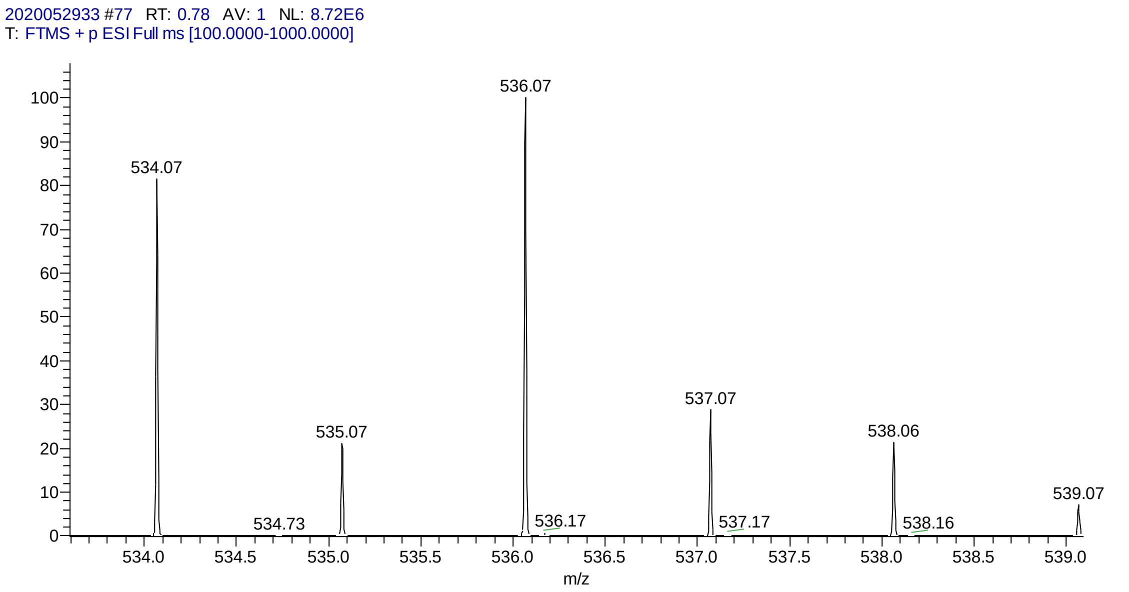

Figure S91. MS spectrum of **17c.**

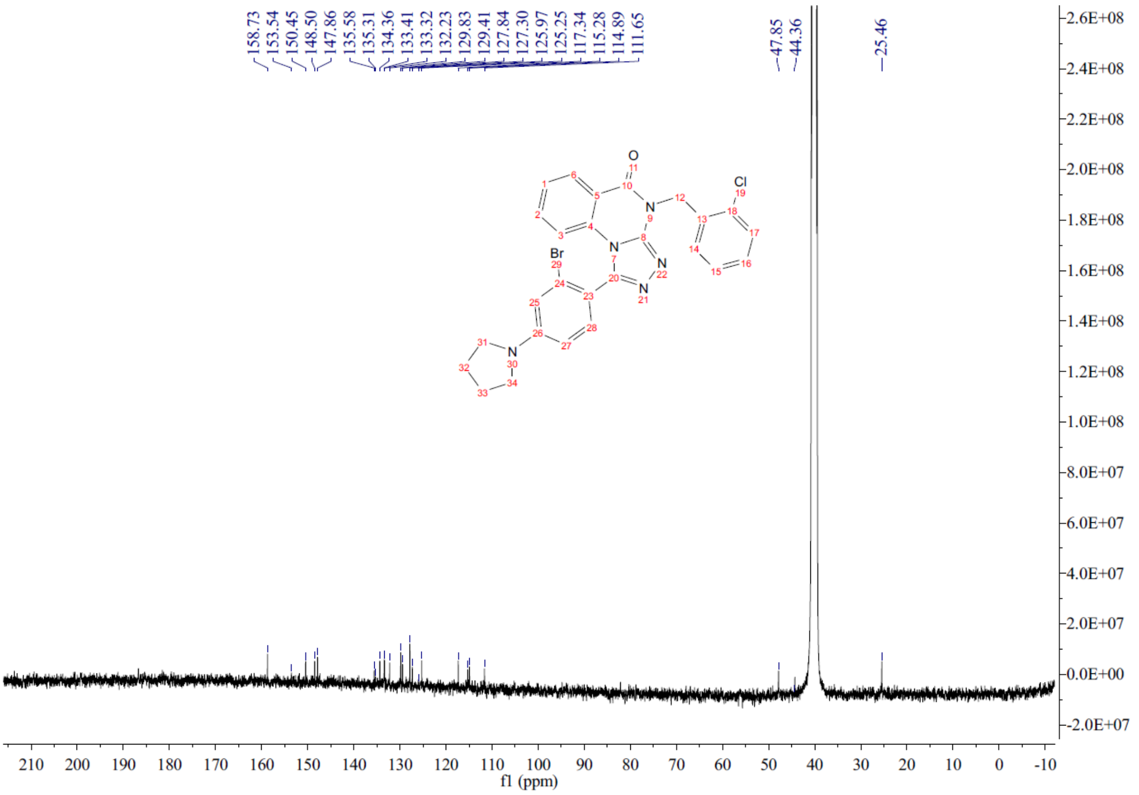

Figure S92.^{13}C-NMR spectrum of **17c**.

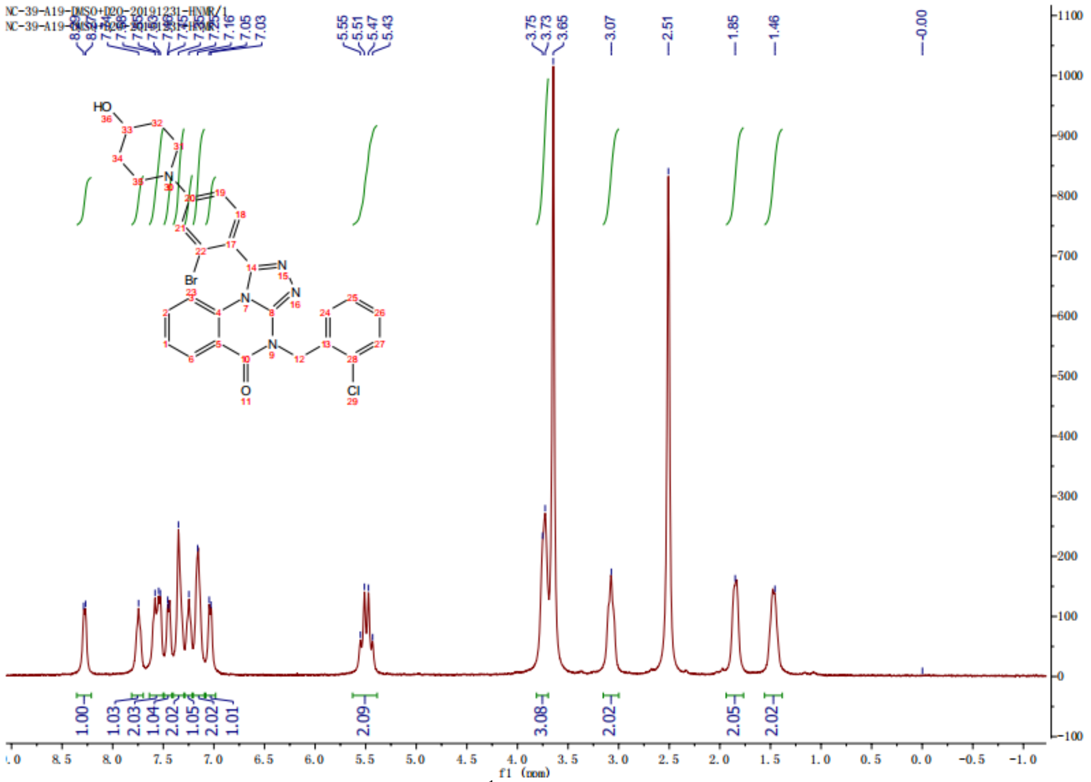

Figure S93. ^{1}H-NMR spectrum of **17d.**

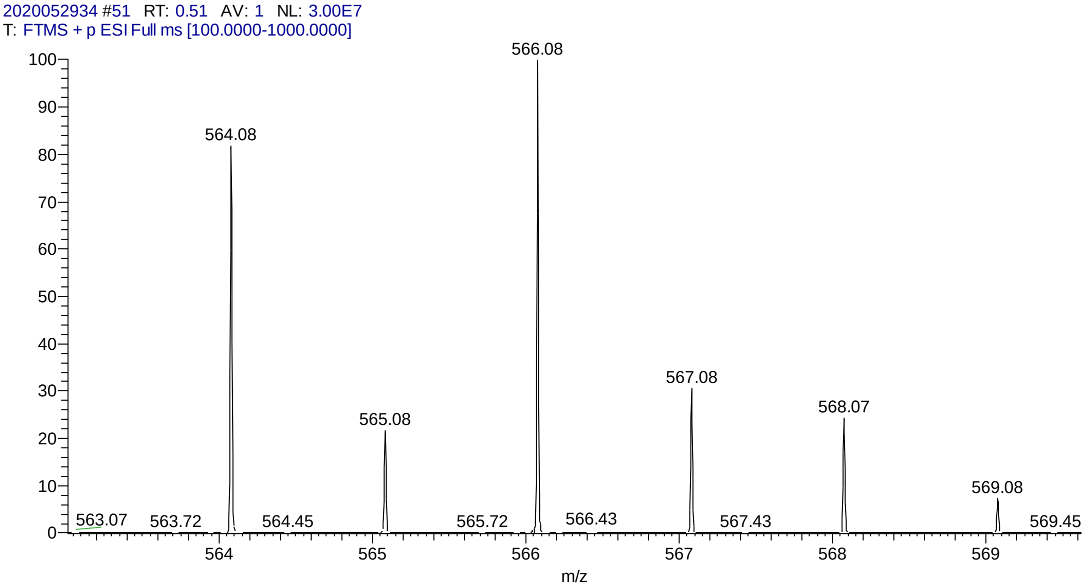

Figure S94. MS spectrum of **17d.**

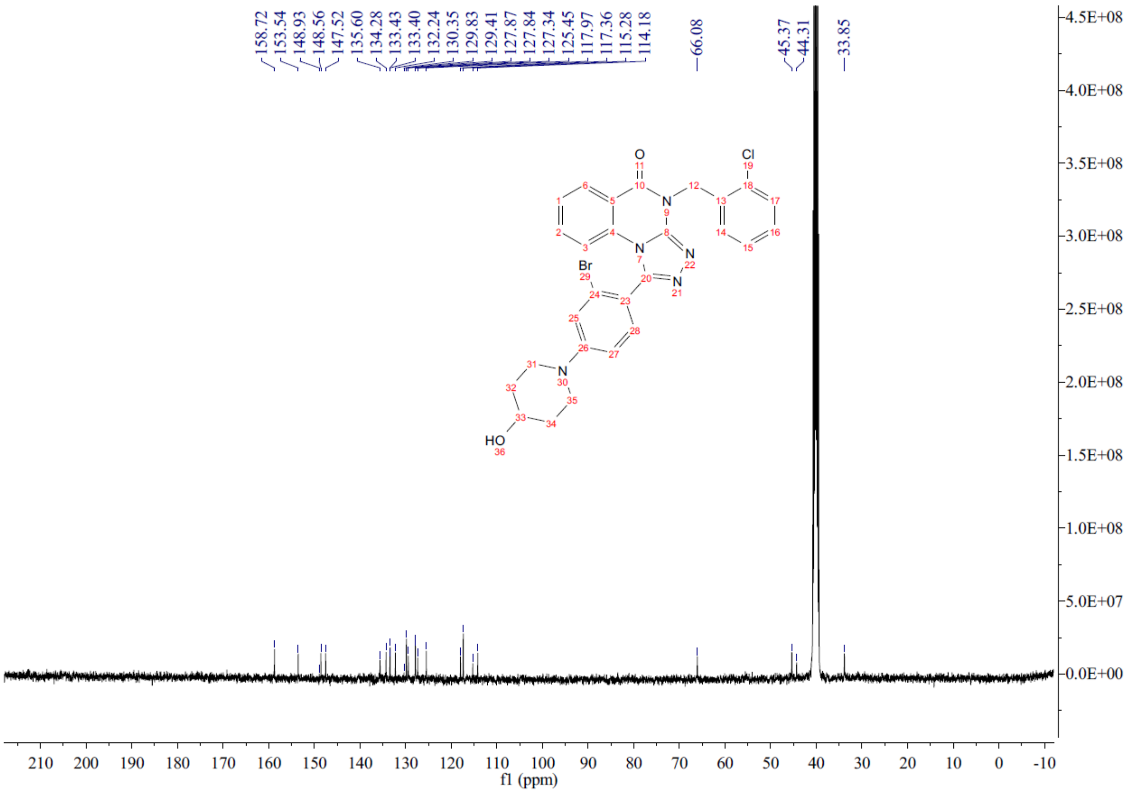

Figure S95. ^{13}C-NMR spectrum of **17d**.

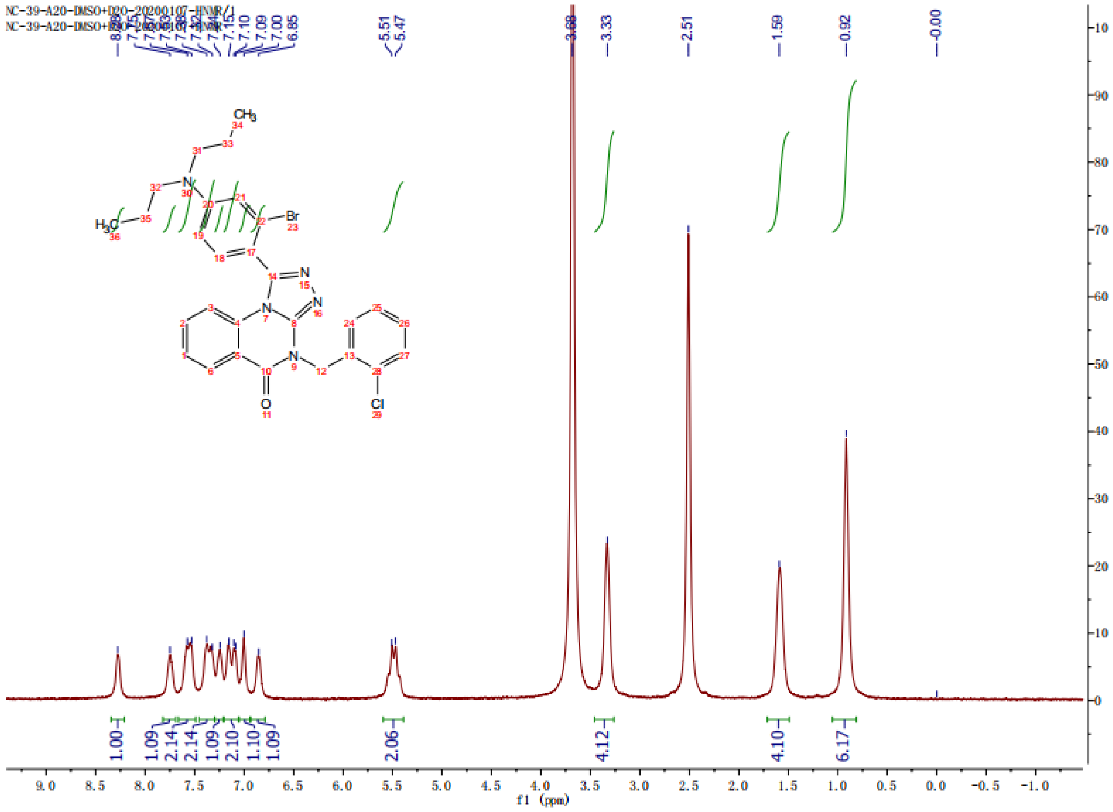

Figure S96. ^{1}H-NMR spectrum of **17e.**

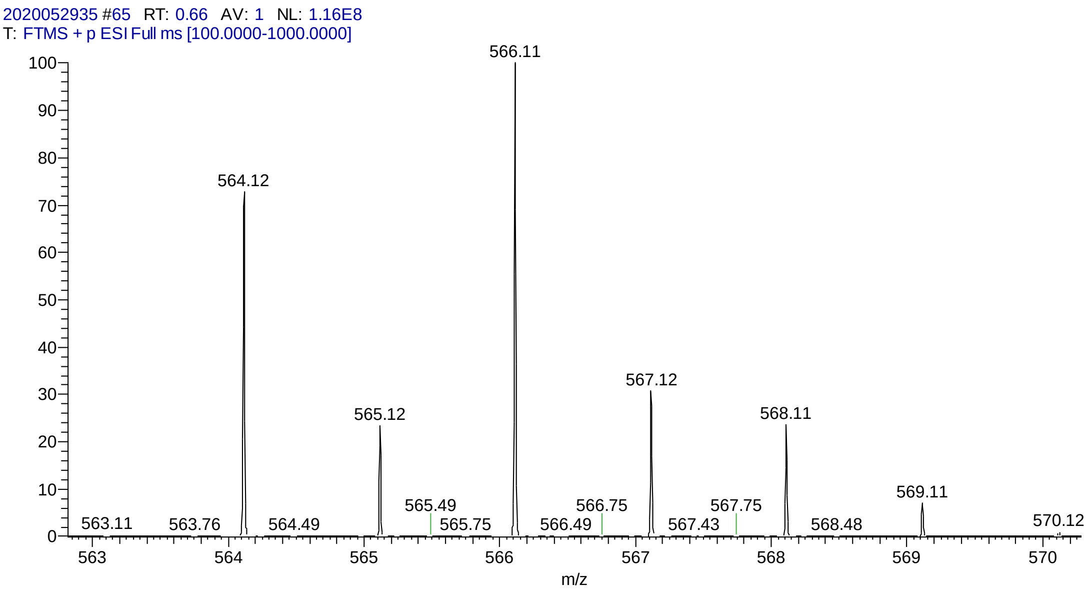

Figure S97. MS spectrum of **17e.**

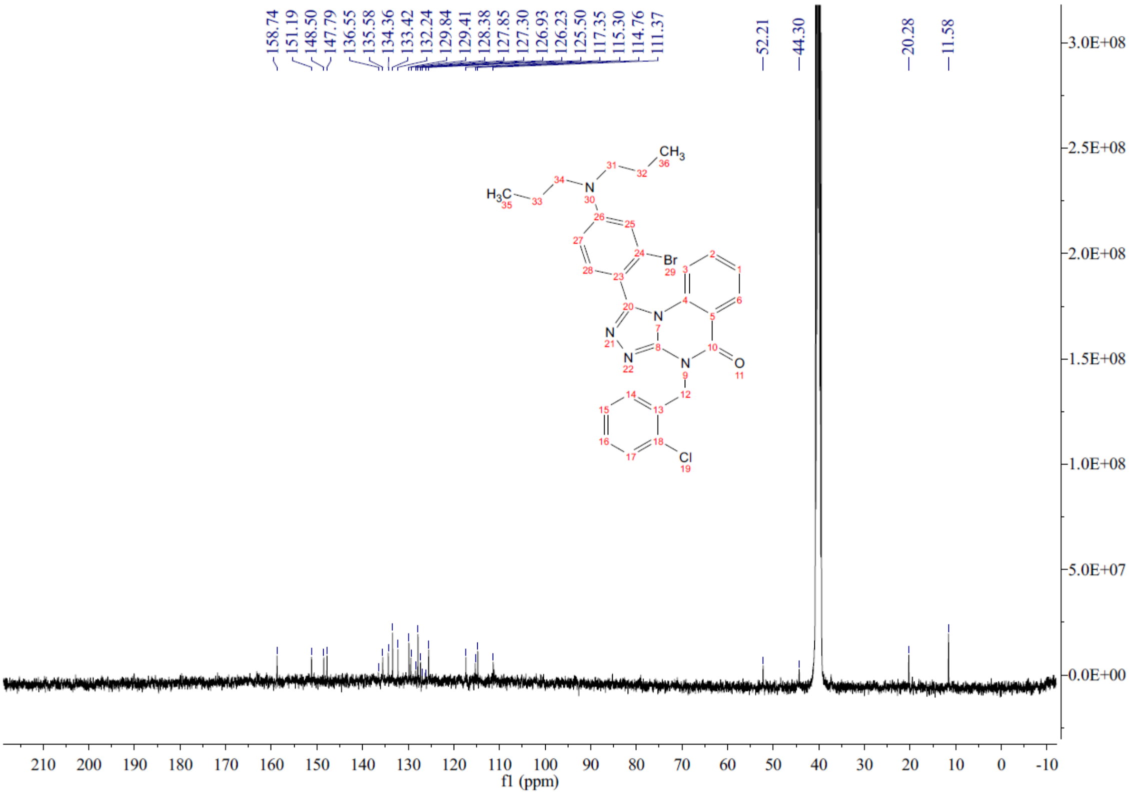

Figure S98.^{13}C-NMR spectrum of **17e**.

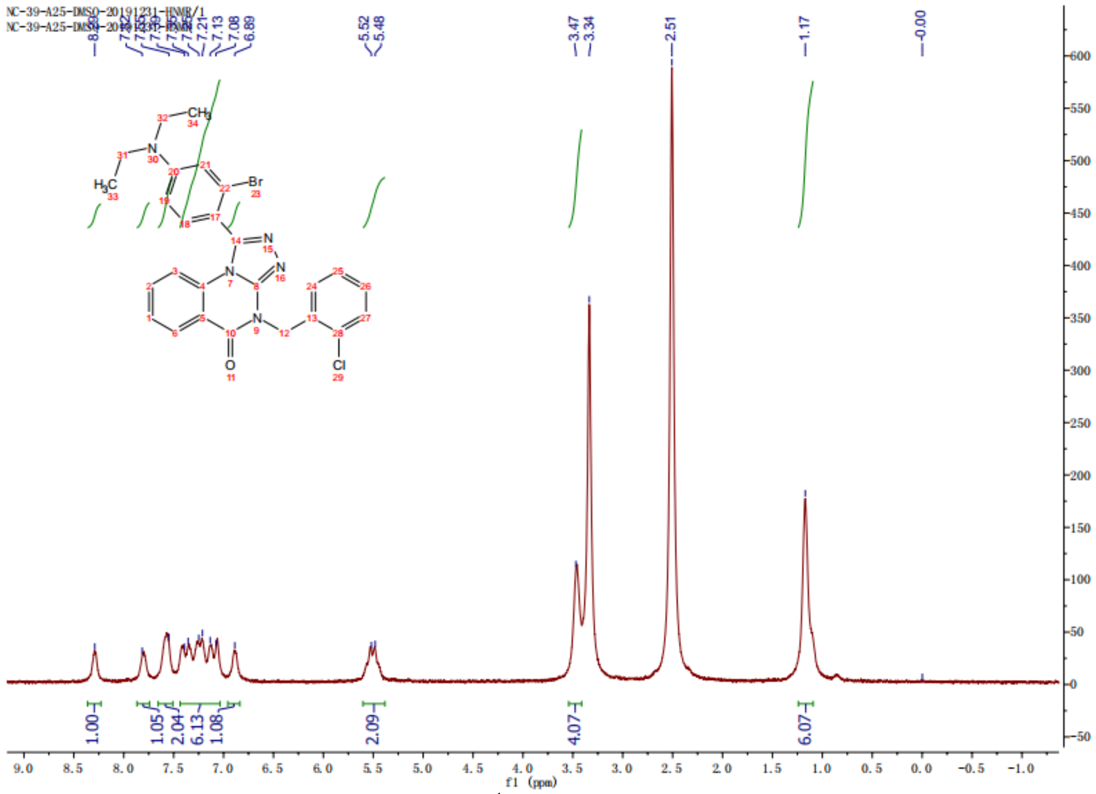

Figure S99. ^{1}H-NMR spectrum of **17f.**

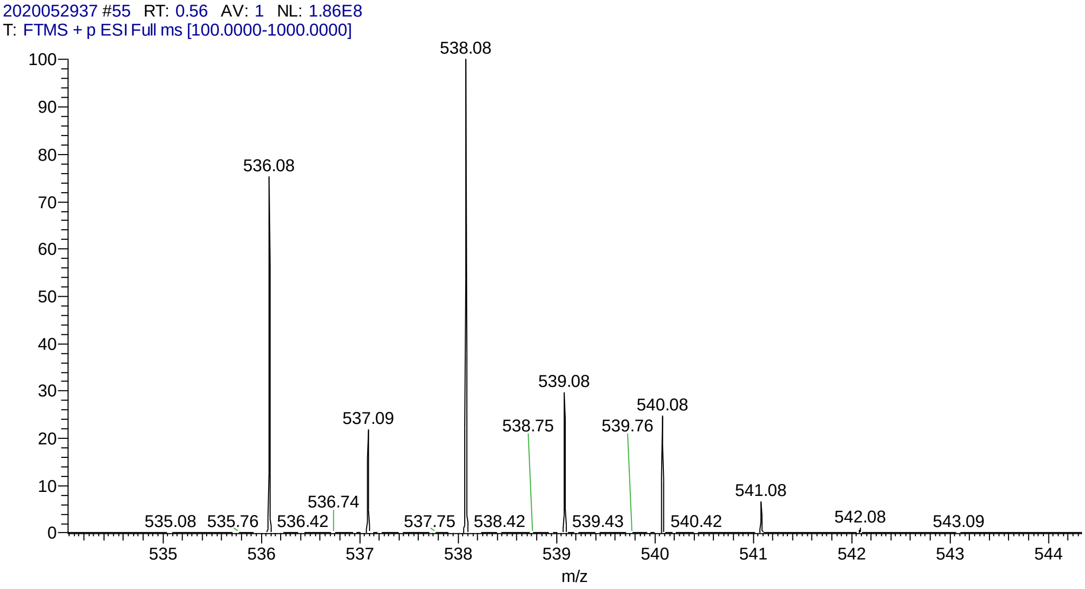

Figure S100. MS spectrum of **17f.**

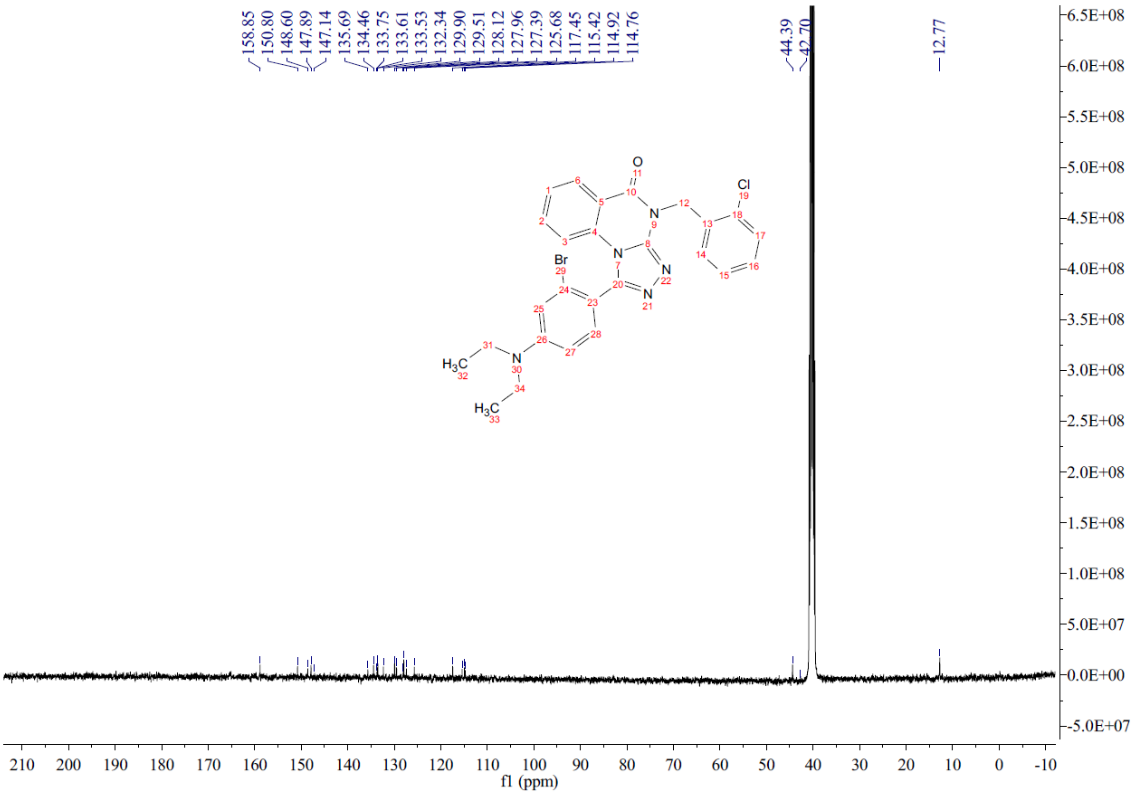

Figure S101.^{13}C-NMR spectrum of **17f**.

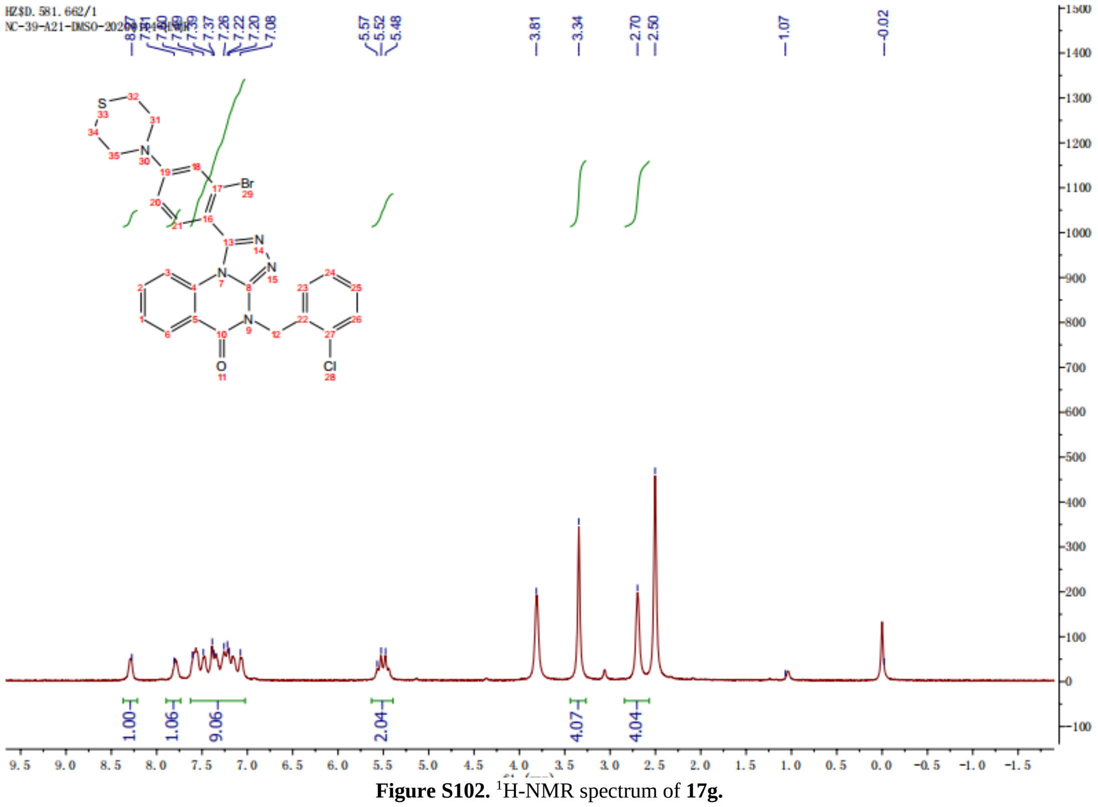

Figure S102. ^{1}H-NMR spectrum of **17g.**

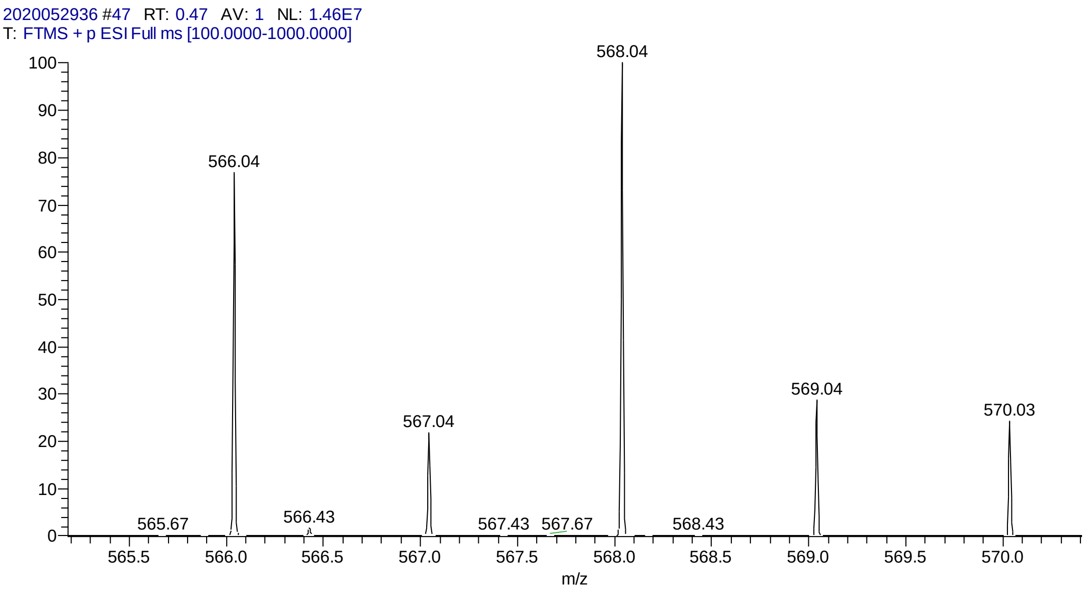

Figure S103. MS spectrum of **17g.**

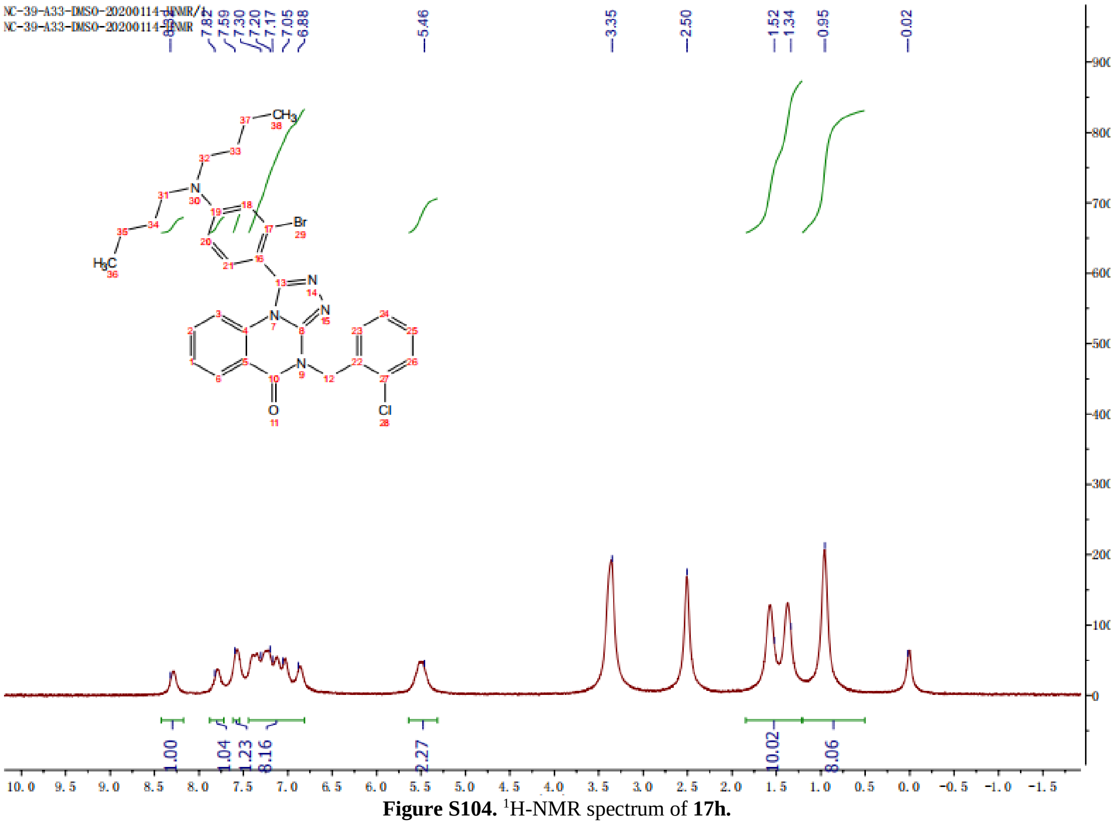

Figure S104. ^{1}H-NMR spectrum of **17h.**

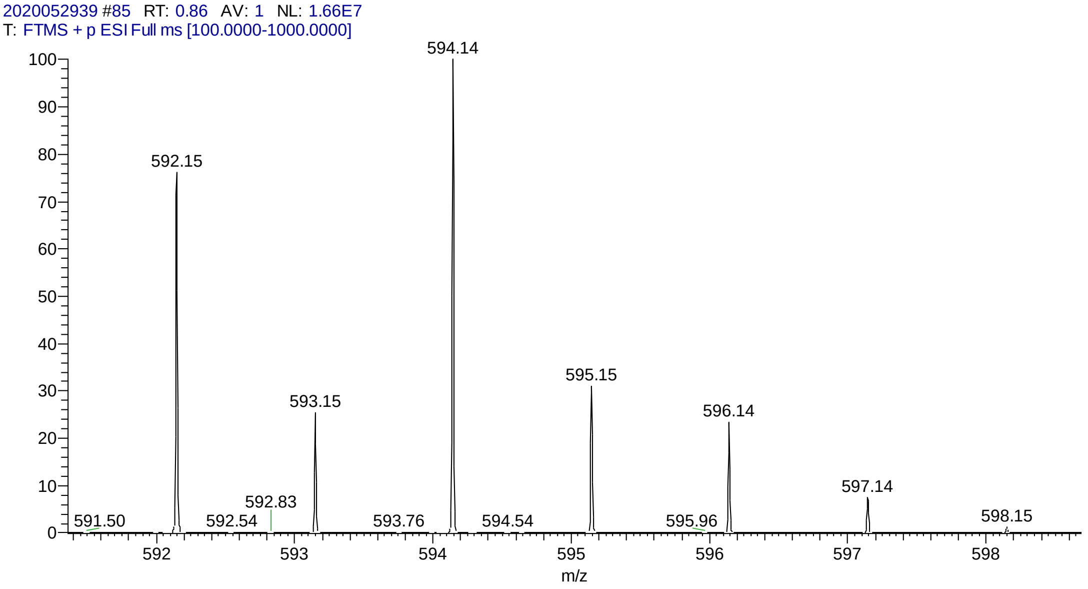

Figure S105. MS spectrum of **17h.**

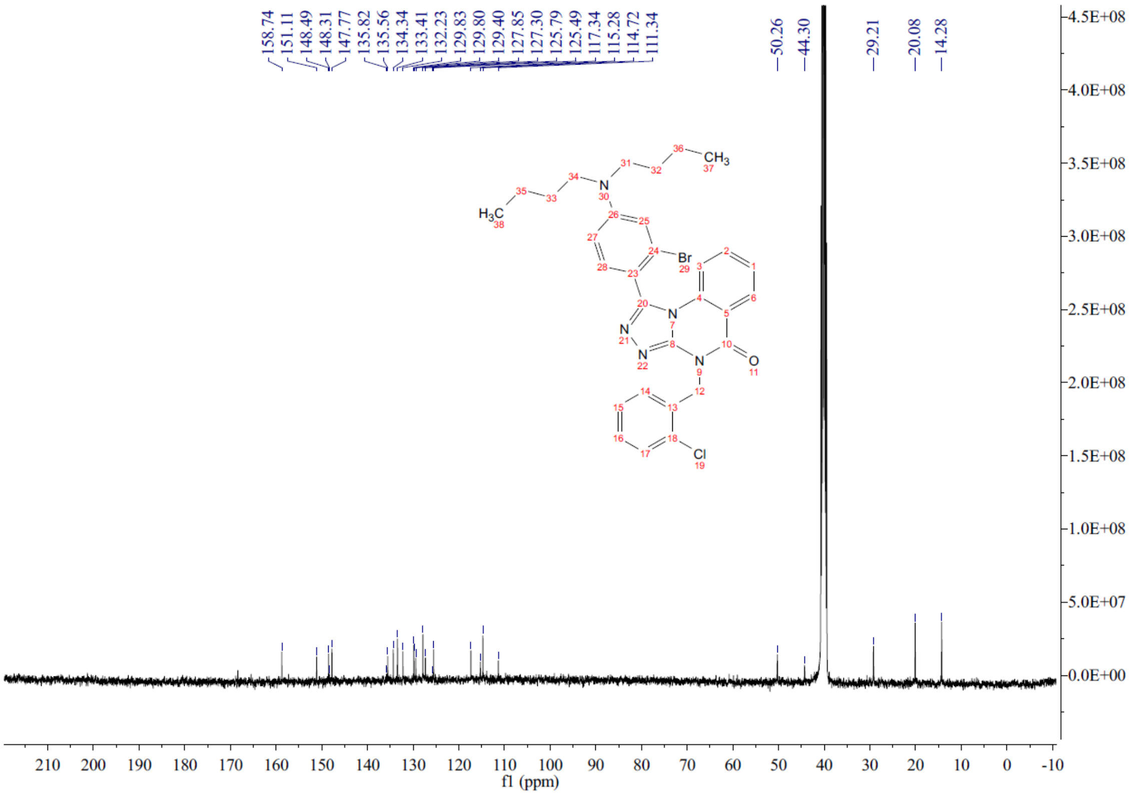

Figure S106.^{13}C-NMR spectrum of **17h**.

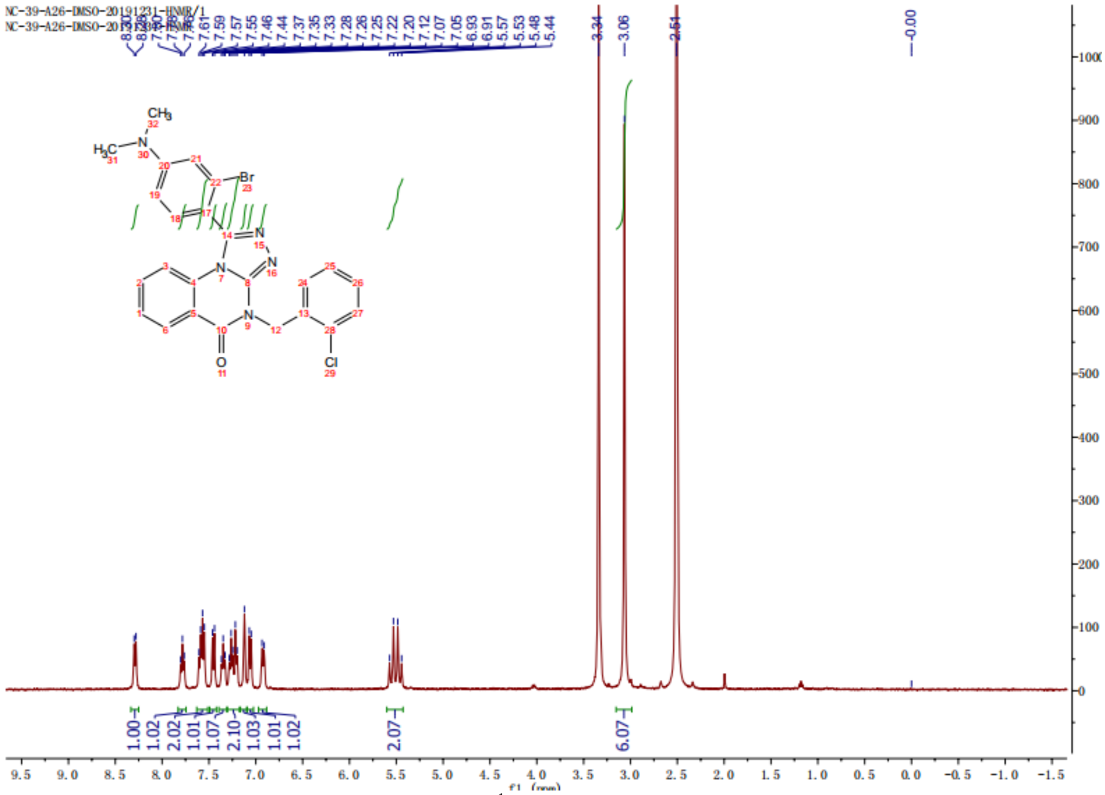

Figure S107. ^{1}H-NMR spectrum of **17i.**

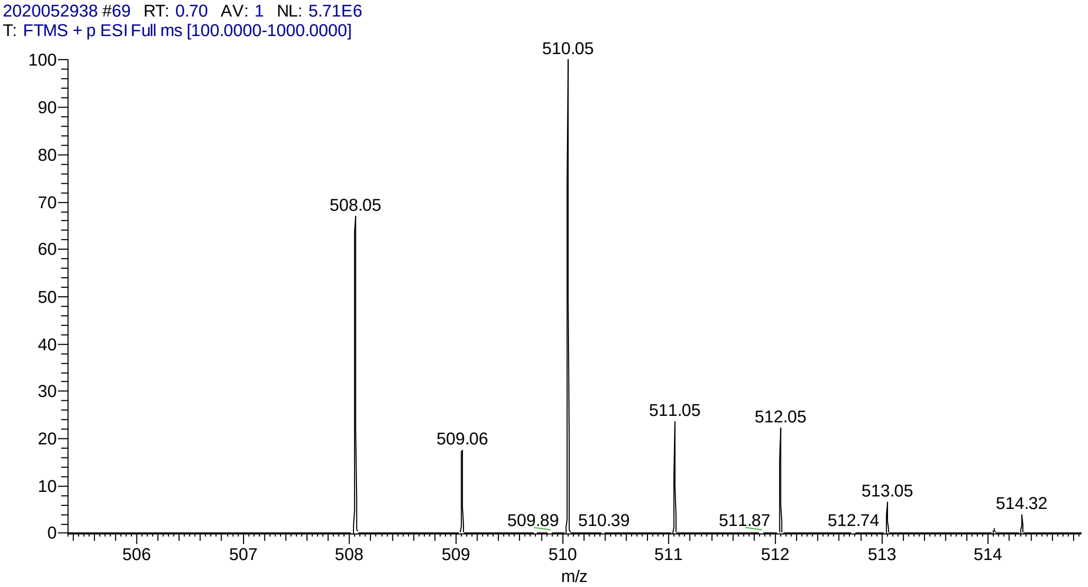

Figure S108. MS spectrum of **17i.**

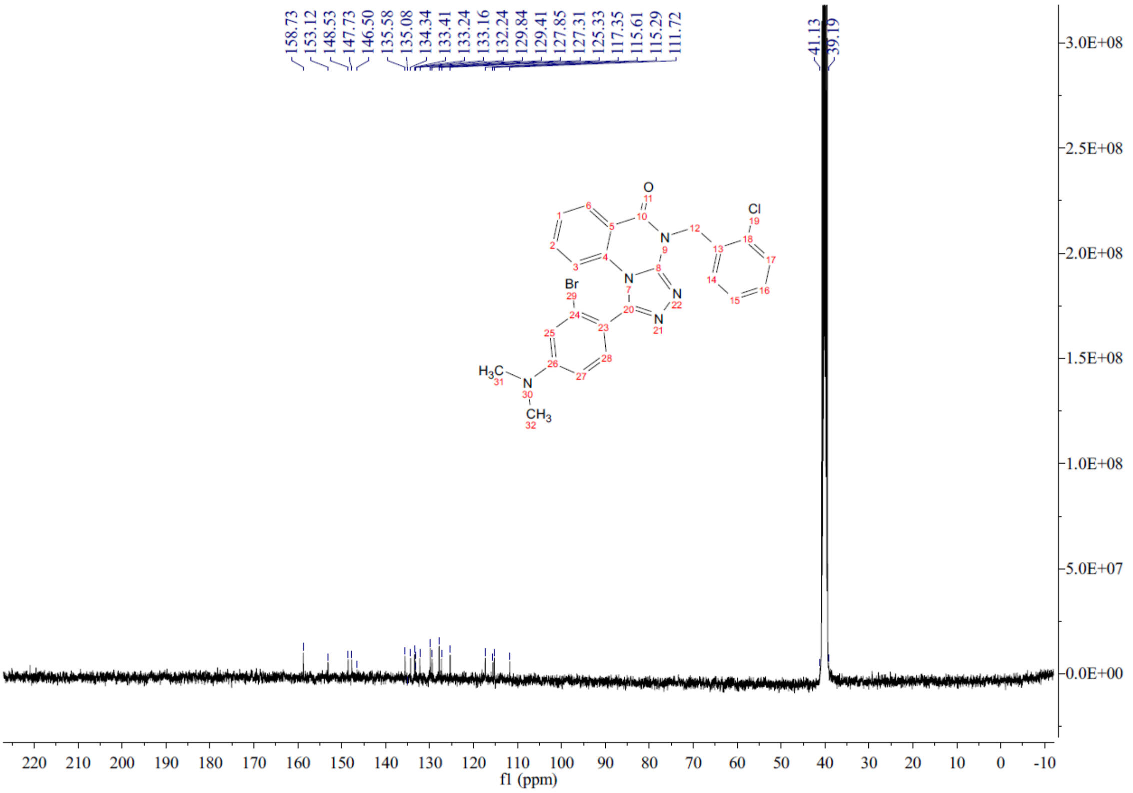

Figure S109.^{13}C-NMR spectrum of **17i**.

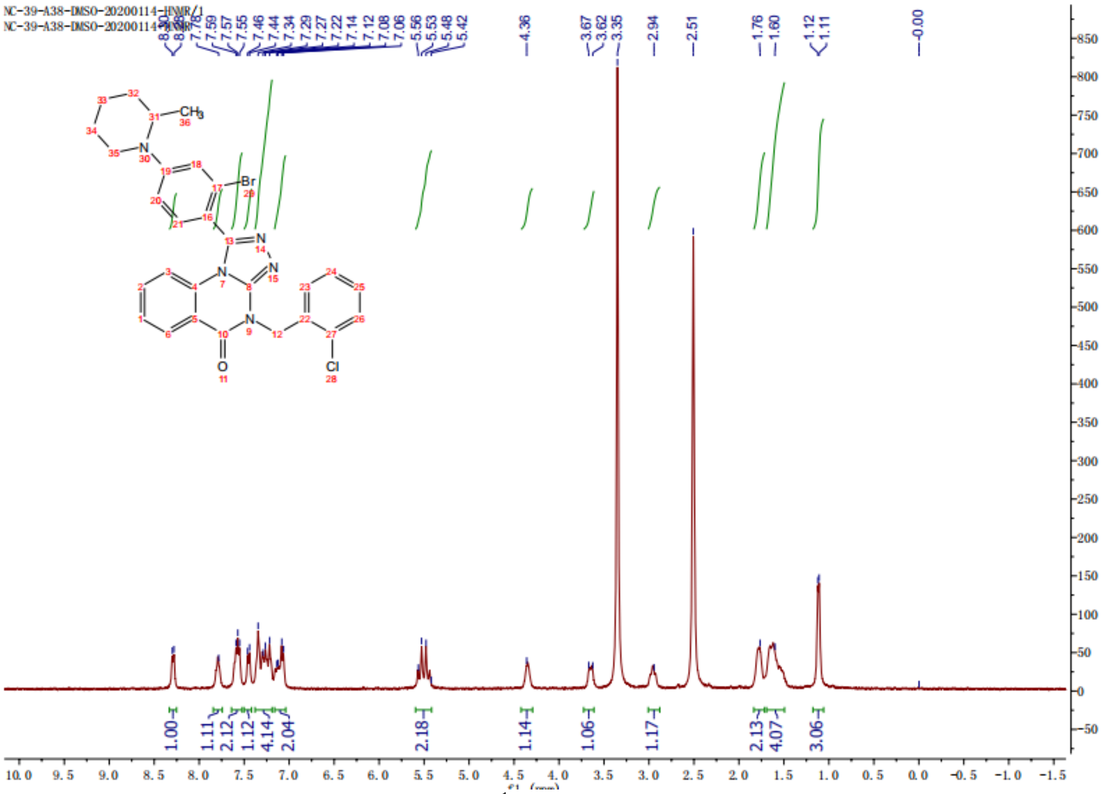

Figure S110. ^{1}H-NMR spectrum of **17j.**

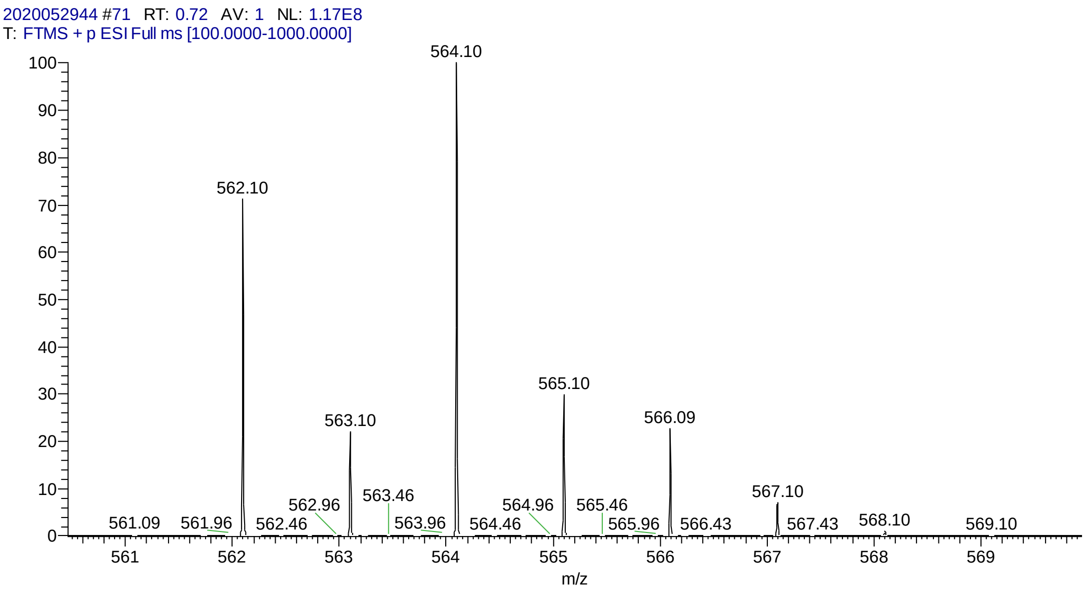

Figure S111. MS spectrum of **17j.**

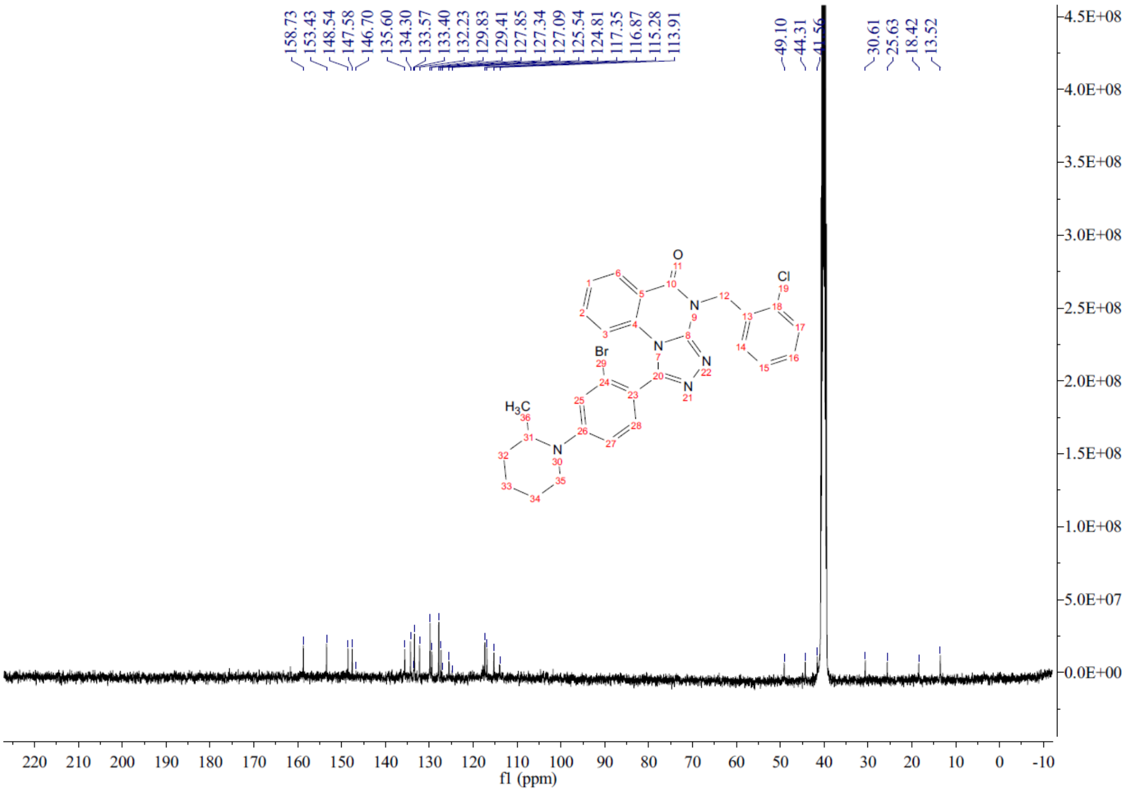

Figure S112.^{13}C-NMR spectrum of **17j**.
